# Dose-Dependent Efficacy of Aripiprazole in Treating Patients With Schizophrenia or Schizoaffective Disorder: A Systematic Review and Meta-Analysis of Randomized Controlled Trials

**DOI:** 10.3389/fpsyt.2021.717715

**Published:** 2021-08-11

**Authors:** Li Qian, Liao Xuemei, Li Jitao, Su Yun'Ai, Si Tianmei

**Affiliations:** Peking University Sixth Hospital, Peking University Institute of Mental Health, NHC Key Laboratory of Mental Health (Peking University), National Clinical Research Center for Mental Disorders (Peking University Sixth Hospital), Beijing, China

**Keywords:** schizophrenia, schizoaffective disorder, aripiprazole, efficacy, strategy, systematic review, meta-analysis

## Abstract

**Purpose:** To compare the efficacy and tolerability of different administration strategies of aripiprazole.

**Methods:** We searched MEDLINE, EMBASE, the Cochrane Central, Web of Science, China National Knowledge Infrastructure(CNKI), and Wanfang Data Knowledge Service Platform(Wanfang) for randomized controlled trials (RCTs) of aripiprazole, using the terms: (aripiprazole) AND (schizophr^*^ OR schizoaff^*^) AND (“syndrome scale” OR PANSS) AND (clini^*^ OR trial). We retrieved study design, participant characteristics, comparison groups, and outcomes from each study.

**Results:** In total, nine RCTs were selected for meta-analysis, which covered ~1,187 participants. We defined two treatment groups that represent different treatment strategies: (1) the high-dose group (the high-dose strategy) rapidly increased to doses higher than 15 mg/day in 2 weeks or began with doses higher than 15 mg/day, otherwise the group was defined as (2) the low-dose group (the low-dose strategy). If the initial or target doses of aripiprazole in a study were all higher than 15 mg/day, the high- and low-dose groups were created based on the relative level of the dose. The high-dose group showed significantly greater reductions in Positive and Negative Syndrome Scale (PANSS) total scores (standardized mean differences = −8.31, 95% confidence interval [CI] = −16.48, −0.13; *P* < 0.01; *I*^2^ = 96%) than the low-dose group. The high-dose group showed superior effects compared with the low-dose group in long-term studies (more than 8 weeks) (standardized mean differences = −13.81, 95% CI = −25.07, −2.55; *P* < 0.01; *I*^2^ = 96%). With exception of somnolence, we did not find significant differences in side effects or discontinuation due to adverse events. Sensitivity analyses produced similar results.

**Conclusion:** The high-dose treatment strategy of aripiprazole for patients with schizophrenia or schizoaffective disorder may bring more benefits without obvious side effects.

## Introduction

Aripiprazole is a second-generation antipsychotic with a unique pharmacological profile distinct from other available antipsychotics (1). Aripiprazole is known as a “dopamine system stabilizer” with a mechanism of action that exerts partial agonism with high affinity at dopamine D_2_ and serotonin-5-HT_1A_ receptors as well as antagonism at serotonin-5-HT_2A_ receptors (2) and shows evidence of good clinical efficacy with a favorable profile of safety and tolerability in patients with schizophrenia (3–6).

As a partial agonist antipsychotic, the clinical usage of aripiprazole is different from other existing antipsychotics. According to the drug label, when administered in the oral formulation for the treatment of schizophrenia, the recommended starting and target dose for aripiprazole is 10 or 15 mg/day, and the maximum dose is 30 mg/day (7). Dosage increases should generally not be made before 2 weeks (7). Premarket clinical studies showed that doses higher than 10 or 15 mg/day were not more effective than 10 or 15 mg/day (7). However, aripiprazole is sometimes administered at a higher dose or the dose is rapidly increased (rapid titration) in clinical practice. Furthermore, a series of recent studies have shown that doses higher than 15 mg/day or rapid dose escalation of aripiprazole may be more effective for treating schizophrenia (8–12). Currently, the dose dependency and benefits of different administration strategies of aripiprazole remain uncertain.

Therefore, we conducted a systematic review and meta-analysis of flexible- or fixed-dose studies of aripiprazole for the treatment of adults with schizophrenia or schizoaffective disorder, examining not only the efficacy of different treatment strategies but also the tolerability and acceptability to provide summative evidence to support better clinical decision-making.

On the basis of aripiprazole package inserts and clinical trials (7, 13), we defined two treatment groups that represent different treatment strategies: (1) the high-dose group (the high-dose strategy) were those that rapidly increased to doses higher than 15 mg/day in 2 weeks or began with doses higher than 15 mg/day; otherwise, the group was defined as (2) the low-dose group (the low-dose strategy). If the initial doses or target dose of aripiprazole in a study were all higher than 15 mg/day, the high- and low-dose groups were created based on the relative level of the dose. Our study followed the Preferred Reporting Items for Systematic Reviews and Meta Analyses (PRISMA) statement for reporting systematic reviews and meta-analyses (14).

## Methods

### Literature Search Strategy

We retrieved studies by systematically searching both English and Chinese medical databases. We searched MEDLINE, EMBASE, the Cochrane Central, and Web of Science, as well as the China National Knowledge Infrastructure (CNKI) and the Wanfang Data Knowledge Service Platform (Wanfang) from the date of inception to July 7th, 2021. We first generated English search terms, which were then independently translated into Chinese by two authors (LQ and LXM). The English search terms were (aripiprazole) AND (schizophr^*^ OR schizoaff^*^) AND (“syndrome scale” OR PANSS) AND (clini^*^ OR trial). We also conducted a manual search for references in selected articles.

### Study Selection

After excluding duplicate studies, the titles and abstracts of the remaining studies were independently reviewed by two investigators (LQ and LXM) to determine whether the studies met the eligibility criteria for inclusion. Disagreements regarding inclusion were resolved by another investigator (SYA).

Studies that met the following eligibility criteria were included: (1) randomized controlled trials (RCTs); (2) aripiprazole (oral formulation) as the intervention drug; (3) patients with any age diagnosed with schizophrenia or schizoaffective disorder (DSM-IV or ICD-10); (4) quantifiable outcomes on the Positive and Negative Syndrome Scale (PANSS) or adverse events; (5) multiple treatment strategies of dose were compared; and (6) published in national journals or core journals (for Chinese medical databases). Studies were excluded if patients had a primary diagnosis of a mental disorder other than schizophrenia or schizoaffective disorder. If more than one study used the same data source with a different follow-up time, we included the most recent publication.

### Data Extraction

All of the data were independently extracted by two investigators (LQ and LXM) using a predefined data extraction form. Disagreements regarding the extracted data were resolved by another investigator (SYA).

The following data were abstracted from individual studies: study details (e.g., first author, year of publication, study setting, and study type), participant characteristics (e.g., sample size, sex, and mean or median age), definition of the intervention and control groups, duration of follow-up, PANSS [total score, positive subscale score and negative subscale score mean changes from baseline with standard deviation (SD)] and treatment-emergent adverse events (TAEs) or discontinuation due to adverse events (DAEs).

### Statistical Analysis

For continuous outcomes [PANSS (15)], we calculated pooled estimates of mean differences (MDs) with two-sided 95% confidence intervals (CIs) using a random effects model if *I*^2^ ≥ 50. We extracted values before and after the intervention in both the study and control arms. We computed the changes in a given group by subtracting the value after the intervention by the corresponding value before the intervention. We used the changes in different groups for the final comparison. For dichotomous outcomes (discontinuation and adverse events), we calculated pooled estimates of risk ratios (RRs). We used an empirical cutoff of 8 weeks (16–18) to define short- or long-term treatment and explored the impacts of treatment length based on subgroup analysis.

We performed a corresponding sensitivity analysis to assess the influence of a single study on the overall pooled estimate. We assessed publication bias in selected studies by funnel plots (19). Additionally, we performed Egger's regression asymmetry tests (20) and considered a *p*-value of < 0.10 to be an indication of statistically significant publication bias. Two investigators (LQ and LJT) independently evaluated the quality of each included study using the Risk of Bias Tool (RoB 2) developed by the Cochrane Collaboration (21). We performed meta-analyses using R (version 3.6.3).

## Results

### Search Results

**Figure 1:**
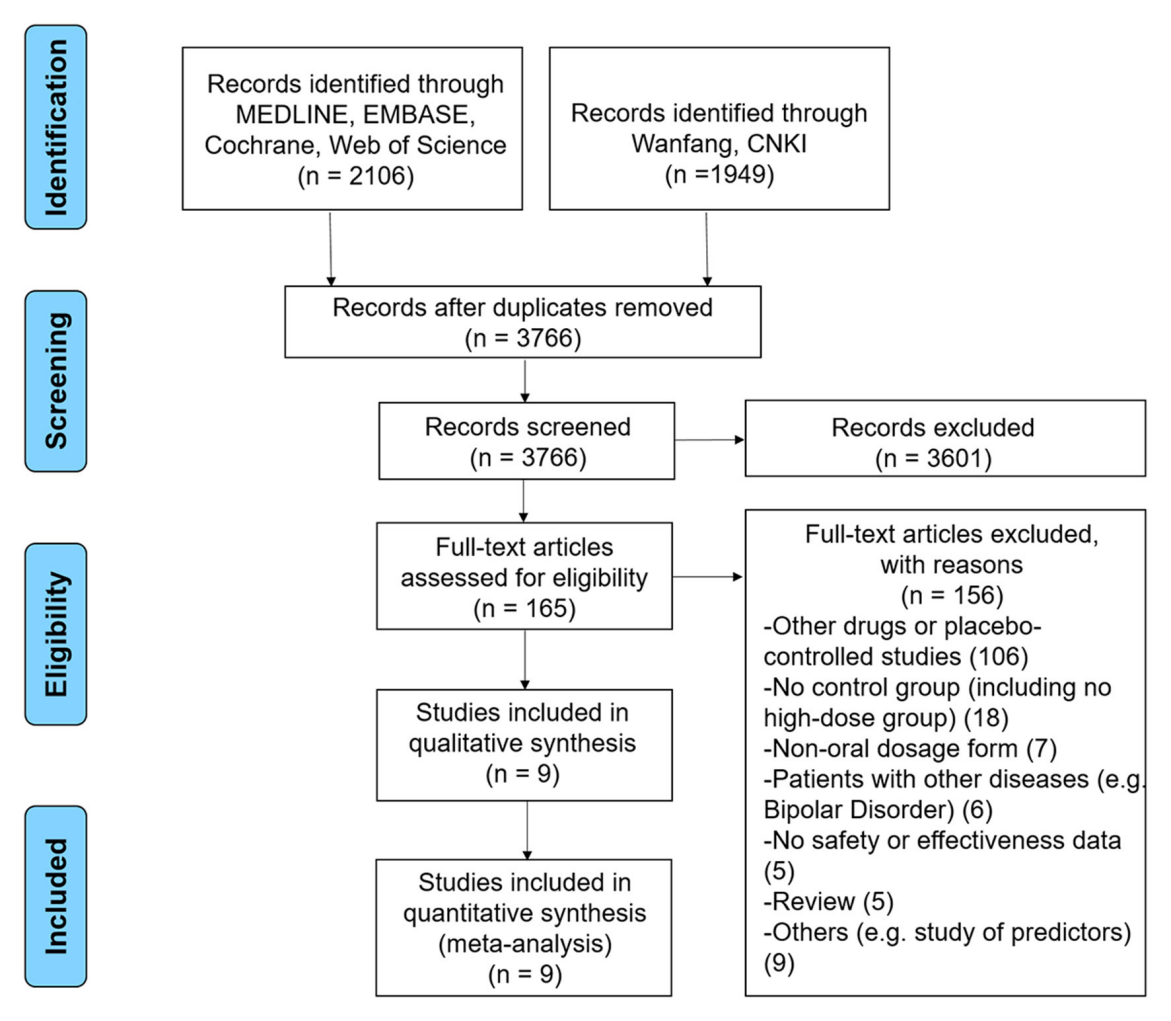
PRISMA flow diagram.

We initially identified 3,766 potentially relevant publications after removing duplicates (Figure 1). We excluded 3,601 articles after screening the titles and abstracts. We excluded another 156 publications following full-text review. Finally, we identified nine RCTs that met our criteria for inclusion in our meta-analysis.

### Study Characteristics

The total sample size was 1,187 patients who were confirmed to have a diagnosis of schizophrenia or schizoaffective disorder. Patient characteristics were similar across studies (Table 1), with the exception of Chen et al. (24), which focused only on female patients. Generally, the study and control arms had a similar proportion of males and females, and most participants were ~40 years old. Two of the nine studies reported PANSS scores without standard deviations and were used only in the safety analysis (3, 4). Four of the nine studies reported both positive and negative PANSS in their outcomes (10, 11, 22, 23), and three studies reported only total PANSS (8, 9, 24). The follow-up time ranged from 4 weeks to 6 months.

**Table 1:** Characteristics of randomized controlled trials.

	**Study design**	***N***	**Age (mean ± SD)**	**Sex (M/F)**	**Diagnosis**	**Treatment**	**Strategy**	**Outcome: Efficacy (mean ± SD)**	**Outcome: Safety *n* (%)**
Kane et al. (3)	Study arm	102	39.3 ± 1.0	70/32	Schizophrenia or schizoaffective disorder	30 mg/d, 4 weeks	Start: 30 mg/day Target: 30 mg/day Time to target dose: Immediate	-	DAE: 8 (7.84)
	Control arm	102	37.8 ± 1.0	76/26		15 mg/d, 4 weeks	Start: 15 mg/day Target: 15 mg/day Time to target dose: Immediate	-	DAE: 9 (8.82)
Casey et al. (22)	Study arm	104	37.1 ± 9.5	72/32	Schizophrenia or schizoaffective disorder	30 mg/d, 8 weeks	Start: 30 mg/day Target: 30 mg/day Time to target dose: Immediate	PANSS-T: −8.18 ± 17.23 PANSS-P: −2.00 ± 5.09 PANSS-N: –2.37 ± 6.13	TAE: 93 (89.42) DAE: 10 (9.62)
	Control arm	103	39.3 ± 10.9	78/25		10 mg, 1 week 20 mg, 1 week 30 mg, 6 weeks	Start: 10 mg/day Target: 30 mg/day Time to target dose: 2 weeks	PANSS-T: −10.11 ± 15.74 PANSS-P: −2.75 ± 5.49 PANSS-N: −2.81 ± 5.53	TAE: 83 (80.58) DAE: 6 (5.83)
Potkin et al. (4)	Study arm	101	40.2	66/35	Schizophrenia or schizoaffective disorder	30 mg/d, 4 weeks	Start: 30 mg/day Target: 30 mg/day Time to target dose: Immediate	-	TAE: 91 (90.10) DAE: 8 (7.92)
	Control arm	101	38.1	73/28		20 mg/d, 4 weeks	Start: 20 mg/day Target: 20 mg/day Time to target dose: Immediate	-	TAE: 92 (91.10) DAE: 11 (10.89)
Findling et al. (11)	Study arm	102	15.4 ± 1.4	65/37	Schizophrenia	2 mg, 2days; 5 mg, 2days; 10 mg, 2days; 15 mg, 2days; 20 mg, 2days; 30 mg, up to 6 weeks	Start: 2 mg/day Target: 30 mg/day Time to target dose: 11 days	PANSS-T: −28.60 ± 8.86 PANSS-P: −8.10 ± 5.91 PANSS-N: −6.60 ± 5.91	DAE: 4 (3.92)
	Control arm	100	15.6 ± 1.3	45/55		2 mg, 2 days; 5 mg, 2 days; 10 mg, up to 6 weeks	Start: 2 mg/day Target: 10 mg/day Time to target dose: 5 days	PANSS-T: −26.70 ± 18.90 PANSS-P: −7.60 ± 5.97 PANSS-N: −6.90 ± 5.97	DAE: 7 (7.00)
Wen and Liu (23)	Study arm	22	34.7 ± 10.0	14/8	Schizophrenia	20-30 mg/d, 12 weeks	Start: 20–30 mg/day Target: 20–30 mg/day Time to target dose: Immediate	PANSS-T: −1.70 ± 9.35 PANSS-P: 0.90 ± 3.75 PANSS-N: −3.20 ± 5.86	TAE: 8 (36.36)
	Control arm	21	35.6 ± 10.2	11/10		5–15 mg/d, 12 weeks	Start: 5–15 mg/day Target: 5–15 mg/day Time to target dose: Immediate	PANSS-T: −2.50 ± 9.56 PANSS-P: 0.50 ± 3.86 PANSS-N: −4.20 ± 5.27	TAE: 10 (47.62)
Chen et al. (24)	Study arm	15	37.29 ± 9.41	0/15	Female schizophrenia with hyperprolactinemia	20 mg/d, 8 weeks	Start: - Target: 20 mg/day Time to target dose: 10 days	PANSS-T: −5.58 ± 12.37	TAE: 5 (33.33) DAE: 1 (6.67)
	Control arm	15	33.94 ± 7.70	0/15		10 mg/d, 8 weeks	Start: - Target: 10 mg/day Time to target dose: 10 days	PANSS-T: −3.69 ± 15.12	TAE: 3 (20.00) DAE: 0
Xu (10)	Study arm	62	42.6 ± 3.8	30/32	Schizophrenia	30 mg/d, 6 months	Start: 30 mg/day Target: 30 mg/day Time to target dose: Immediate	PANSS-T: −40.38 ± 10.41 PANSS-P: −8.76 ± 3.41 PANSS-N: −11.05 ± 5.42	TAE: 6 (9.68)
	Control arm	62	42.3 ± 2.2	28/34		20 mg/d, 6 months	Start: 20 mg/day Target: 20 mg/day Time to target dose: Immediate	PANSS-T: −19.76 ± 9.77 PANSS-P: −4.37 ± 4.15 PANSS-N: −6.42 ± 5.73	TAE: 3 (4.84)
Cheng et al. (9)	Study arm	39	43.5 ± 4.1	27/12	Schizophrenia	30 mg/d, 6 months	Start: 30 mg/day Target: 30 mg/day Time to target dose: Immediate	PANSS-T: −40.56 ± 8.04	TAE: 5 (12.82)
	Control arm	39	42.4 ± 3.9	26/13		20 mg/d, 6 months	Start: 20 mg/day Target: 20 mg/day Time to target dose: Immediate	PANSS-T: −15.45 ± 8.32	TAE: 4 (10.26)
Zhu (8)	Study arm	50	28.5 ± 9.5	20/30	Schizophrenia	30 mg/d, 4 months	Start: 30 mg/day Target: 30 mg/day Time to target dose: Immediate	PANSS-T: −28.50 ± 9.97	–
	Control arm	50	29.0 ± 12.0	19/31		20 mg/d, 4 months	Start: 20 mg/day Target: 20 mg/day Time to target dose: Immediate	PANSS-T: −18.74 ± 9.39	–

*M, male; F, female; PANSS-T, PANSS total score; PANSS-P, PANSS positive subscale score; PANSS-N, PANSS negative subscale score; DAE, discontinuation due to adverse events; TAE, treatment-emergent adverse events*.

### Efficacy

**Figure 2:**
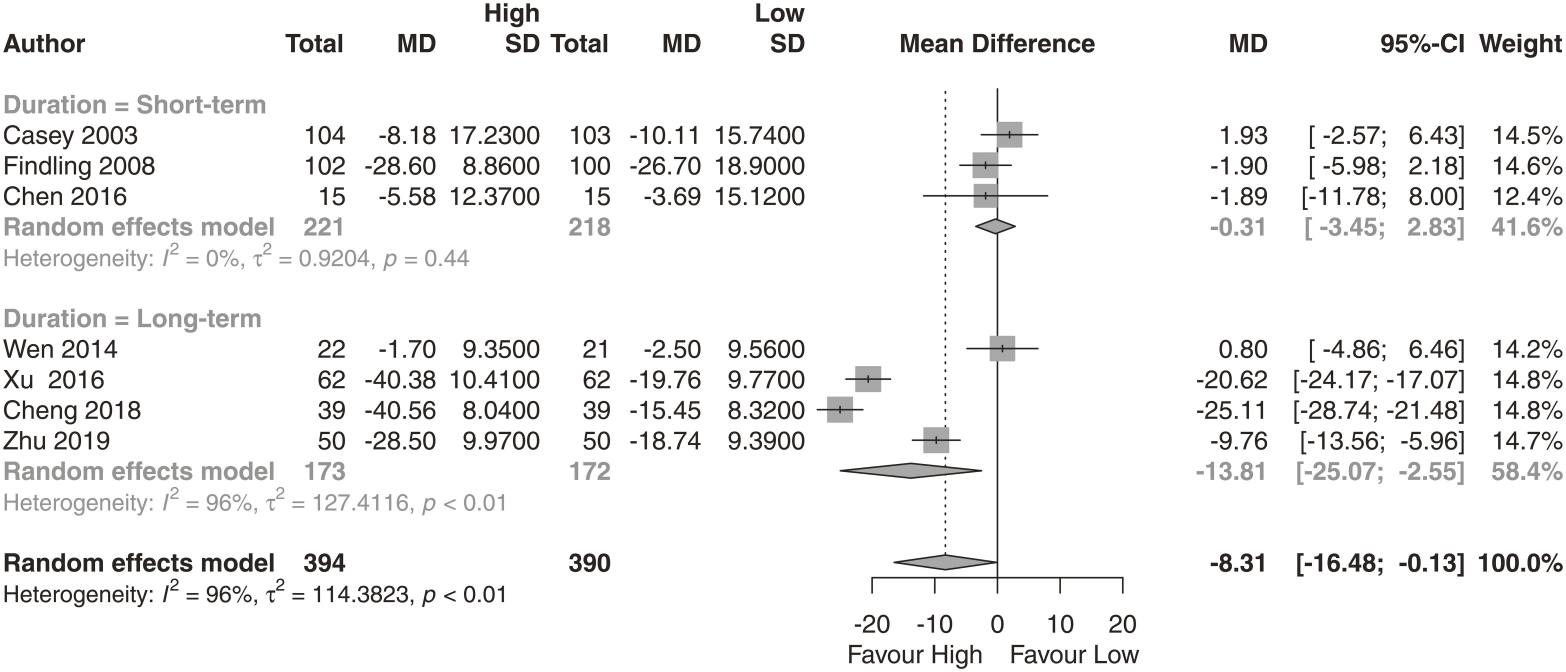
PANSS total score. High, high-dose strategy; Low, low-dose strategy.

**Figure 3:**
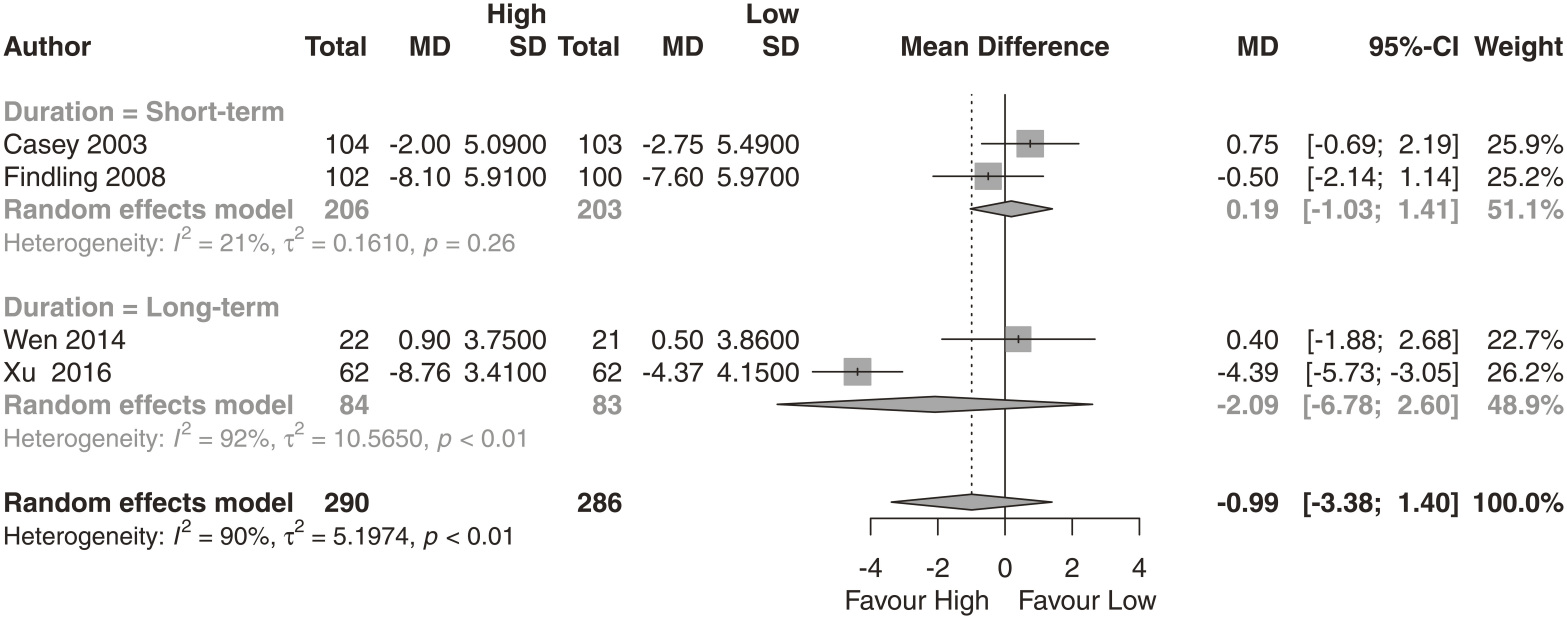
PANSS positive score. High, high-dose strategy; Low, low-dose strategy.

**Figure 4:**
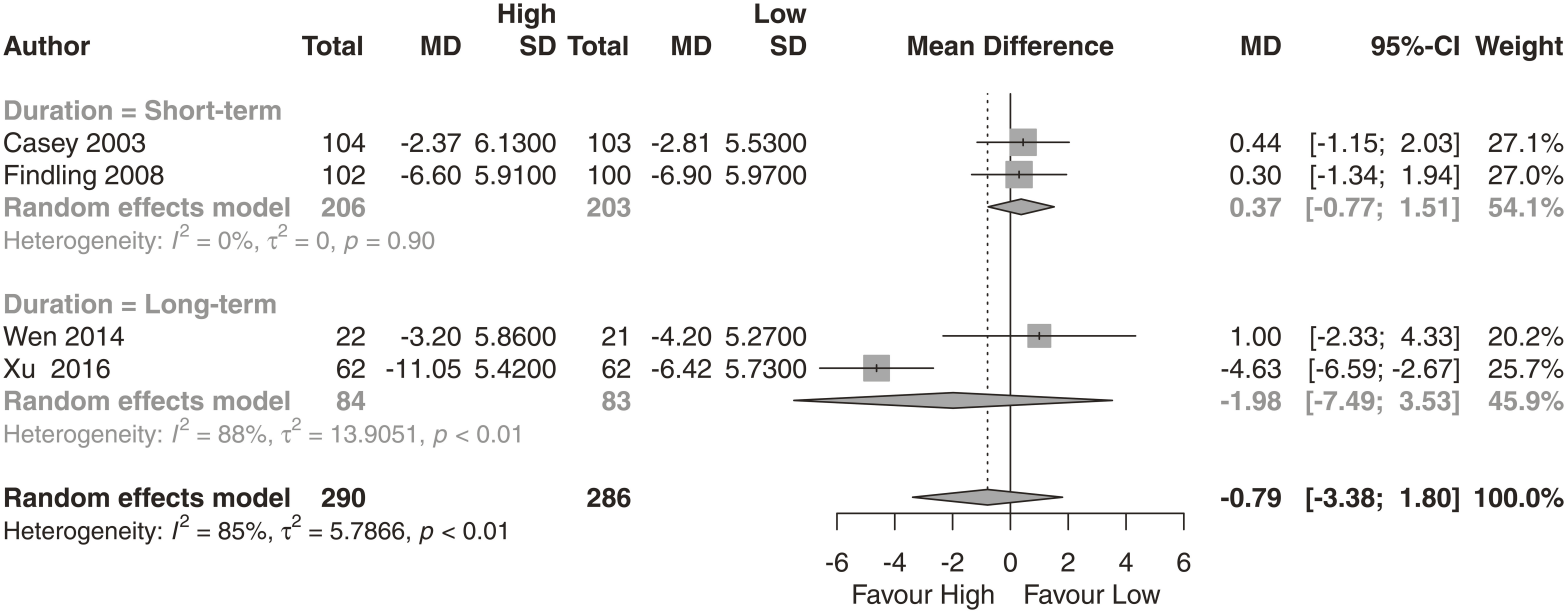
PANSS negative score. High, high-dose strategy; Low, low-dose strategy.

We observed that the high-dose group showed a significantly greater reduction in PANSS total scores (Figure 2; *N* = 7; *MD* = −8.31, 95% CI = −16.48, −0.13) than the low-dose group. No significant difference was found in reductions from baseline for PANSS positive subscale scores (Figure 3; *N* = 4; *MD* = −0.99, 95% CI = −3.38, 1.40) or PANSS negative subscale scores (Figure 4; *N* = 4; *MD* = −0.79, 95% CI = −3.38, 1.80) between the high-dose group and the low-dose group.

Regarding the reductions in PANSS total, positive and negative scores, we conducted an additional subgroup analysis on the length of the clinical trial. We found that the high-dose group had a superior effect compared with the low-dose group in long-term studies (Figure 2; *N* = 4; *MD* = −13.81, 95% CI = −25.07, −2.55).

### Treatment-Emergent Adverse Events

**Figure 5:**
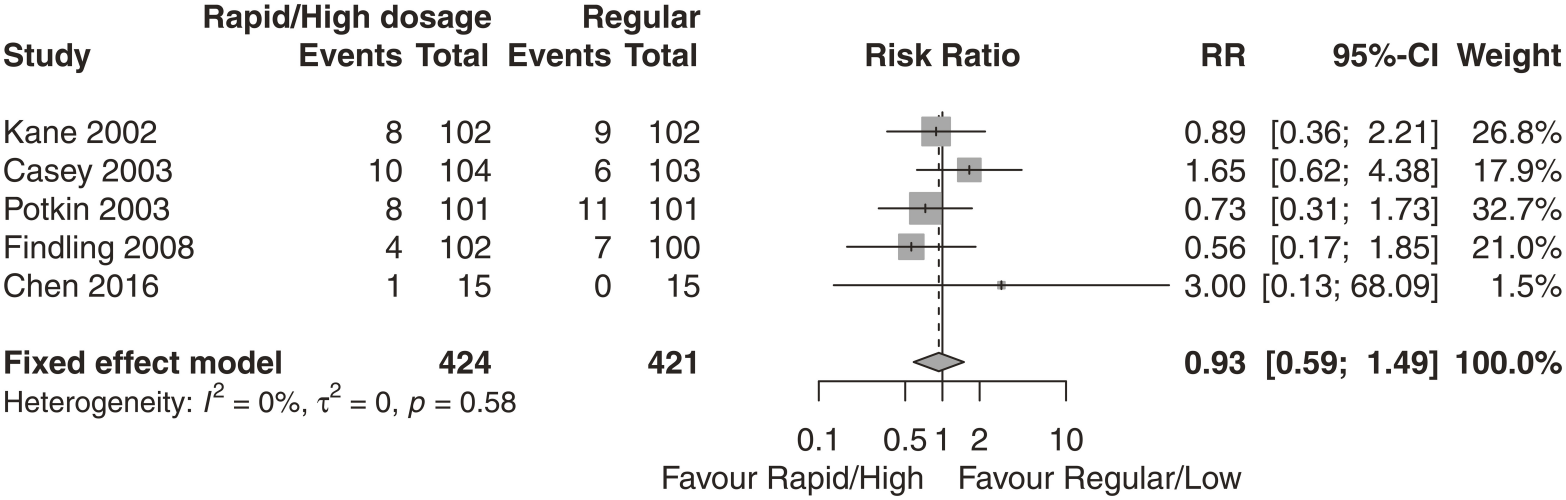
Discontinuation due to adverse events (DAEs).

We did not observe any significant differences in the proportion of patients who discontinued the treatment between groups (Figure 5; *N* = 5, *n* = 424; *RR* = 0.93, 95% CI = 0.59, 1.49; *P* = 0.58, *I*^2^ = 0%). The proportion of patients who experienced somnolence was significantly higher in the high-dose group than in low-dose group (Table 2; *N* = 4, *n* = 812; *RR* = 1.98, 95% CI = 1.13, 2.98; *P* = 0.001, *I*^2^ = 48.4%). We did not observe any significant differences in the proportion of patients who experienced headache, anxiety, insomnia, nausea, akathisia, agitation, blurred vision, dizziness, vomiting, asthenia, or diarrhea.

**Table 2:** Treatment-emergent adverse events (high- vs. low-dose group).

**AE**	**No. of studies**	**Treatment group**	**Control group**	**RR [95% CI]**	***P***	***I*** ^**2**^ **(%)**
Headache	7	91/558	94/556	0.97 [0.75, 1.25]	0.81	28.0
Insomnia	6	91/477	105/475	0.86 [0.68, 1.10]	0.24	0
Anxiety	5	58/375	71/375	0.82 [0.60, 1.12]	0.21	0
Nausea	5	54/427	51/425	1.05 [0.74, 1.51]	0.78	0
Akathisia	5	59/427	59/425	1.00 [0.71, 1.39]	0.99	47.1
Blurred vision	4	9/302	4/304	2.01 [0.70, 5.81]	0.19	0
Somnolence	4	62/407	31/405	1.98 [1.13, 2.98]	0.001	48.4
Vomiting	4	40/407	30/405	1.34 [0.48, 3.77]	0.58	73.6
Agitation	3	40/306	39/303	1.03 [0.70, 1.52]	0.89	0
Dizziness	3	22/253	27/252	0.64 [0.21, 1.96]	0.44	61.0
Asthenia	2	14/201	11/203	1.28 [0.60, 2.76]	0.52	0
Diarrhea	2	14/204	12/203	1.16 [0.55, 2.44]	0.70	0

**Figure 6:**
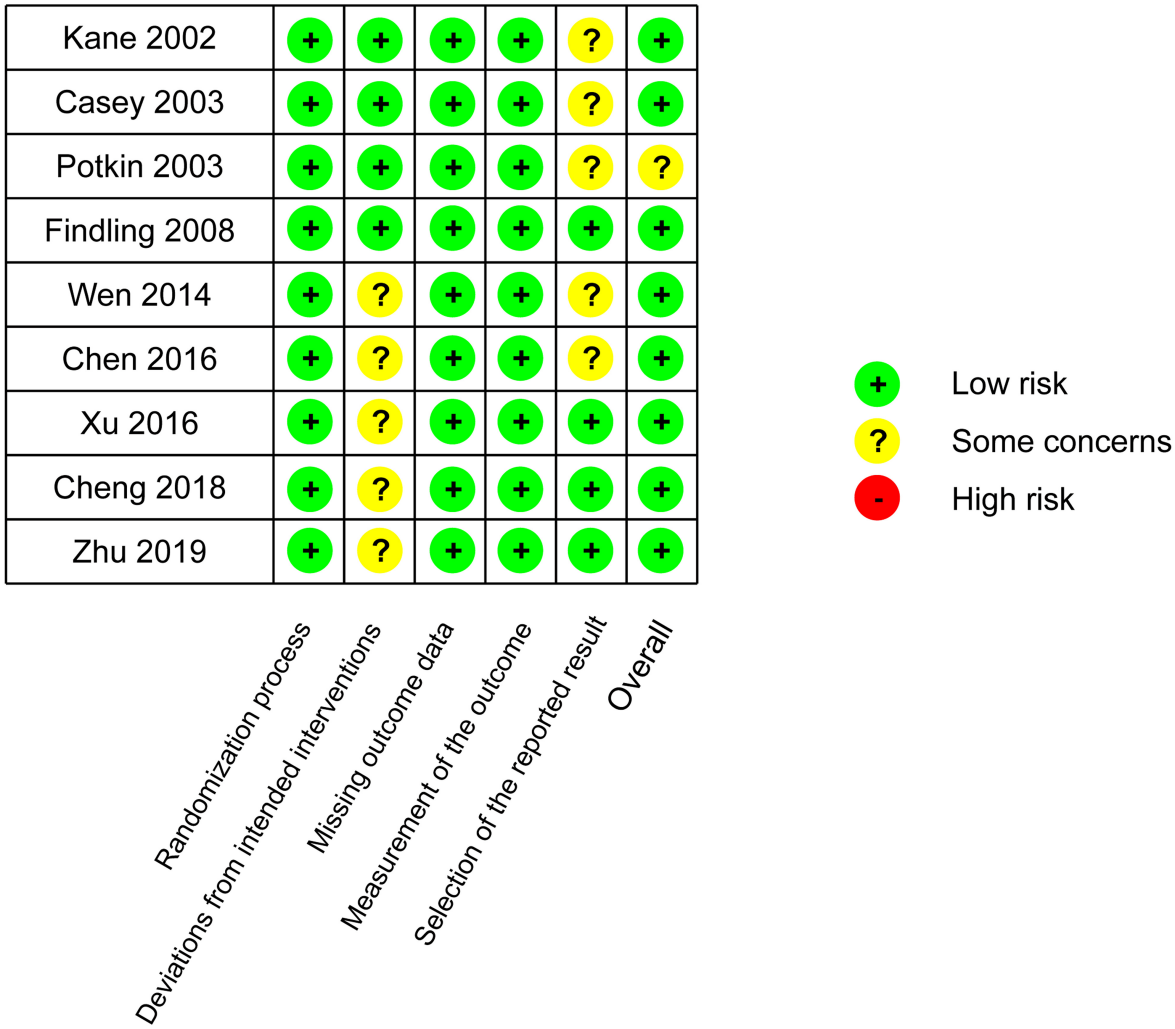
Risk-of-bias assessment.

**Figure 7:**
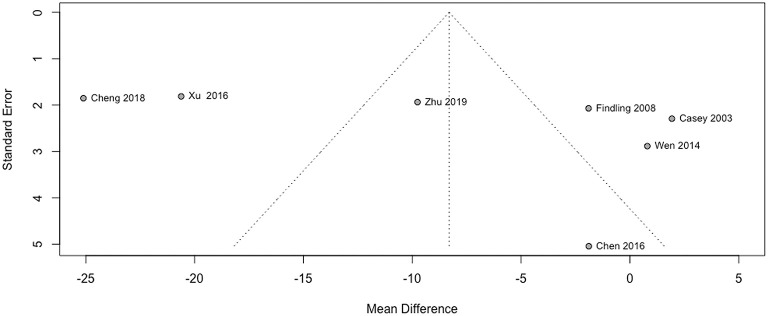
Funnel plot.

**Figure 8:**
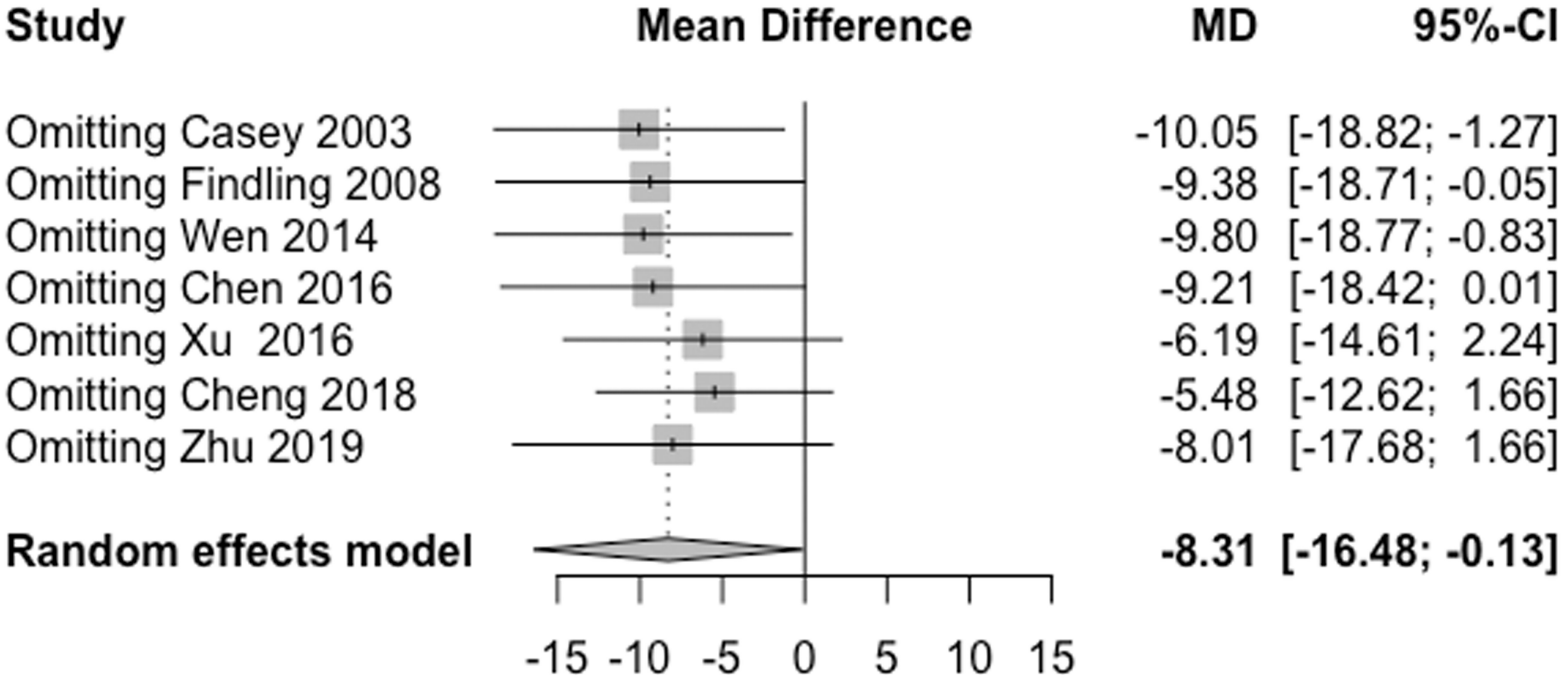
Sensitivity analysis.

No study had high risk of bias (Figure 6). Funnel plots and Egger's test are shown in Figure 7. We did not find evidence that the results may have involved publication bias (Egger's test, *P* = 0.24). We conducted a sensitivity analysis and did not identify any study that reversed this result (Figure 8).

## Discussion

We analyzed the dose-response relationship of aripiprazole and provided evidence for an optimal dose strategy. We found that the high-dose strategy had a statistically significant superior effect in long-term treatment. Compared with the low-dose group, the high-dose group (at least 20 mg/day) showed, on average, benefits of 8.31 points (95% CI: 0.13–16.48) on PANSS total score performance. The subgroup analysis of long-term treatment showed a significant benefit of 13.81 points (95% CI: 2.55–25.07) in PANSS total scores. However, no significant difference was found in reductions in PANSS positive or negative subscale scores between the high-dose group and the low-dose group.

In several studies, patients given high-dose aripiprazole (30 mg/day) for more than 8 weeks showed significant improvements in PANSS total scores compared with the low-dose group (8–10, 23). Aripiprazole exhibits linear pharmacokinetics, as Kinghorn and McEvoy (25) have found. Participants in the group receiving 30 mg/day had an early time of peak plasma concentration with no saturation effect. The benefit of this high dose could also be related to the high affinity of aripiprazole for dopamine D_2_ and D_3_ receptors. The occupancy of these receptors was observed to have a significant increasing dose-dependent relationship with aripiprazole (26).

On the basis of existing evidence on the tolerability of aripiprazole (3), we provided further evidence that the difference in adverse events between high- and low-dose groups was generally comparable. Studies have shown that the side effects of aripiprazole may be related to the dose or titration schedule (27–29) when compared with the placebo group, but evidence has rarely been provided for the comparison between dose groups. In our study, we found that the rates of DAE were similar in both dose groups (*RR* = 0.93, 95% CI: 0.59, 1.49). As shown in Table 2, we did not find any statistically significant differences in any adverse effect except somnolence (*RR* = 1.98, 95% CI: 1.13, 2.98) between the two dose groups. Patients with schizophrenia or schizoaffective disorder were able to tolerate a relatively higher dose.

These results were not free of limitations. First, the total number of clinical trials related to aripiprazole remains limited. Second, several studies were conducted quite early, whereas others were conducted more recently. Differences in patients' birth cohort or race distribution may have introduced bias. Further RCTs with various antipsychotic treatment strategies are needed to improve clinical decision making.

These data support aripiprazole treatments with a high-dose strategy for patients with schizophrenia or schizoaffective disorder. We found that a treatment strategy with a relatively higher dose or more rapid dose escalation than the standard usage (i.e., starting at 10–15 mg/day and increasing dose safter a minimum of 2 weeks) (7) may bring potential benefits. The adverse events and rates of discontinuation were similar across groups. Treatment with aripiprazole was well-tolerated. In summary, we illustrated a potential association between dosage and efficacy that provided evidence for better clinical decision-making.

## Data Availability Statement

The original contributions generated for the study are included in the article/Supplementary Material, further inquiries can be directed to the corresponding author/s.

## Author Contributions

LQ, LXM, LJT, SYA, and STM conceived the study. LQ, LXM, and SYA reviewed the abstracts and the papers, and extracted the data from the selected articles. LQ and LJT preformed statistical analyses. LQ, SYA, and STM wrote and reviewed the manuscript. All authors contributed to the article and approved the submitted version.

## Conflict of Interest

Chengdu Kanghong Pharmaceutical Group Co., Ltd. assisted in obtaining the full text for some studies. The company was not involved in the study design, data collection, analysis, interpretation of data, the writing of this article or the decision to submit it for publication. The authors declare that the research was conducted in the absence of any commercial or financial relationships that could be construed as a potential conflict of interest.

## Publisher's Note

All claims expressed in this article are solely those of the authors and do not necessarily represent those of their affiliated organizations, or those of the publisher, the editors and the reviewers. Any product that may be evaluated in this article, or claim that may be made by its manufacturer, is not guaranteed or endorsed by the publisher.
