# Supplementary material for: Dose-Dependent Efficacy of Aripiprazole in Treating Patients With Schizophrenia or Schizoaffective Disorder: A Systematic Review and Meta-Analysis of Randomized Controlled Trials
Source: Front Psychiatry. 2021 Aug 11;12:717715. doi: 10.3389/fpsyt.2021.717715 (PMC8385236; doi:10.3389/fpsyt.2021.717715)
Supplement: Supplementary file 1 [file Data_Sheet_1.ZIP › supplementray material-9 RCTs/7 Chen 2016.pdf]

·临床研究·

阿立哌唑治疗利培酮所致高催乳素血症女性患者的量效关系

陈红梅,张荣珍,陈景旭,刘艳红,李　伟,谭云龙,杨甫德
(北京大学回龙观临床医学院,北京 100096)

[摘要]　**目的**:评估不同剂量阿立哌唑治疗利培酮所致女性精神分裂症患者高催乳素血症的疗效及安全性。**方法**:61例利培酮所致高催乳素血症女性精神分裂症患者,随机分至阿立哌唑5 mg组($n=16$)、10 mg组($n=15$)、20 mg组($n=15$)和安慰剂组($n=15$),维持原有利培酮剂量不变,随访8周。于基线及第2,4和8周末检测血清催乳素(PRL)水平,于基线及8周末评定阳性和阴性症状量表(PANSS)、Barnes静坐不能评定量表(BAS)、Simpson-Angus量表(SAS)及UKU副作用评定量表(UKU)。**结果**:第8周末,各阿立哌唑剂量组PRL水平均较治疗前显著下降($P<0.01$),而安慰剂组治疗前后PRL水平无统计学差异。第8周末,阿立哌唑10 mg组和20 mg组PRL下降率、正常率均显著高于阿立哌唑5 mg组和安慰剂组($P<0.01$),而阿立哌唑10 mg组与20 mg组之间无统计学显著差异。研究终点,4组PANSS,CGI-S,SAS和BAS评分及不良反应发生率均无显著性差异。**结论**:阿立哌唑可有效治疗利培酮所致女性精神分裂症患者的高催乳素血症,最佳治疗剂量为10 mg·d^{-1}。

[关键词]　阿立哌唑;利培酮;高催乳素血症;精神分裂症;量效关系
[中图分类号]　R971.4　　[文献标志码]　A　　[文章编号]　1003-3734(2016)05-0569-05

Dose-effect relationship of aripiprazole on hyperprolactinemia induced by risperidone in female patients

CHEN Hong-mei, ZHANG Rong-zhen, CHEN Jing-xu, LIU Yan-hong, LI Wei, TAN Yun-long, YANG Fu-de
(*Beijing Huilongguan Hospital, Peking University HuiLongGuan Clinical Medical School, Beijing* 100096, *China*)

[**Abstract**]　**Objective**: To evaluate the efficacy and safety of different doses of aripiprazole on hyperprolactinemia caused by risperidone in female patients with schizophrenia. **Methods**: Sixty-one female schizophrenic patients with hyperprolactinemia induced by risperidone were randomly divided into aripiprazole 5 mg group ($n=16$), 10 mg group ($n=15$), 20 mg group ($n=15$) and placebo group ($n=15$) added on to fixed dose risperidone treatment for 8 weeks. Serum prolactin levels(PRL) were measured at baseline, 2, 4 and 8 weeks. The Positive and Negative Syndrome Scale (PANSS), the Barnes AkathisiaScale (BAS), Simpson Angus Scale (SAS) and the UKU Side Effects Rating Scale (UKU) were assessed at baseline and week 8. **Results**: PRL levels in the three aripiprazole groups were significantly reduced at the end of the study compared with the baseline($P<0.01$), while there was no significant difference between pre- and post-treatment in the placebo group. At week 8, the decline rate and the normal ratio of PRL levels were significantly higher in the 10 mg and 20 mg groups than those in the 5 mg and placebo groups($P<0.01$), but no significant difference was observed between the 10 mg and 20 mg groups. The PANSS, SAS and BAS scores and the incidence of side effects did not differ among all the treatment groups at the end point. **Conclusion**: Aripiprazole may be effective for treating risperidone-induced hyperprolactinemiain female patients with schizophrenia, and the optimum dose for aripiprazole appears to be 10 mg·d^{-1}.

[**Key words**]　aripiprazole; risperidone; hyperprolactinemia; schizophrenia; dose-effect relationship

[基金项目]　优秀人才基金(2009D003014000001);北京市医管局重点医学专业发展计划(ZYLX201409);北京市科技计划项目(D121100005012005)
[作者简介]　陈红梅,女,硕士在读,主治医师/病区主任,主要从事临床精神病学研究。联系电话:15001366115,E-mail:shenqidoudou941@sina.com。
[通讯作者]　陈景旭,男,硕士,主任医师/科主任,主要从事临床精神病学研究。联系电话:13681394260,E-mail:chenjx1110@163.com。

利培酮所致高催乳素血症发生率高达 70% ~ 100%，且女性显著高于男性。高催乳素血症可能引起闭经、泌乳、男子乳房发育、性功能障碍、骨质疏松等，从而严重限制其临床应用[1]。近年来，多项临床试验证实[2-5]，联用多巴胺受体部分激动剂阿立哌唑能有效改善利培酮所致的高催乳素血症。本课题组进行的量效关系研究发现，阿立哌唑治疗利培酮所致高催乳素血症最佳剂量为 10 $mg\cdot d^{-1}$[6]。众所周知，利培酮和阿立哌唑对催乳素（prolactin，PRL）水平的影响存在很大的性别差异[7-8]，临床上更应关注患者个体，优化个体的治疗方案。因此，本研究探讨了阿立哌唑治疗利培酮所致女性精神分裂症患者高催乳素血症的量效关系，为临床合理用药提供依据。

资料与方法

1 病例选择

1.1 纳入标准 符合美国精神疾病诊断与统计手册第四版（Diagnostic and Statistical Manual of Mental Disorders, Fourth Edition, DSM-IV）精神分裂症的诊断标准；年龄 18 ~45 岁；单用利培酮 3 ~8 $mg\cdot d^{-1}$治疗，且剂量固定至少 6 周；阳性和阴性症状量表（Positive and Negative Syndrome Scale，PANSS）评分≤70 分，病情稳定；无内分泌疾病史；血清 PRL 水平 > 24 $\mu g\cdot L^{-1}$；受试者能充分知晓和理解本试验的研究内容，并签署知情同意书。本研究方案和知情同意书获得北京回龙观医院医学伦理委员会审核批准。

1.2 排除标准 合并脑、心、肝、肾、内分泌系统等重大躯体疾病；合并酒精、药物滥用及精神发育迟滞；合用影响 PRL 的药物。

1.3 剔除标准 违背研究方案；依从性差，不能按照试验方案完成试验；资料不全，影响疗效和安全性的判断。

1.4 脱落标准 严重药物不良反应；撤除知情同意书；研究者考虑患者的病情而认为应该退出研究；失访；破盲。

2 试验药品

试验药：阿立哌唑口腔崩解片（成都康弘药业集团股份有限公司，规格：每片 5 mg，批号：101202），安慰剂（成都康弘药业集团股份有限公司生产）外型、口味、颜色与阿立哌唑口腔崩解片相同，完全符合安慰剂制备要求。

3 治疗方案

本研究为随机、双盲、安慰剂对照研究，双盲治疗期为 8 周。采用 SPSS 生成随机数字表，将筛查合格的 61 例受试者按照 1∶1∶1∶1 比例随机分至阿立哌唑 5 mg 组（n = 16）、10 mg 组（n = 15）、20 mg 组（n = 15）和安慰剂组（n = 15）。

研究期间，保持原有利培酮（西安杨森制药有限公司生产，规格：每片 1 mg，批号：091023453）剂量不变。患者入组后，10 d 内加至晨服试验药品 4 片（阿立哌唑 5 mg 组：阿立哌唑 1 片，安慰剂 3 片；阿立哌唑 10 mg 组：阿立哌唑 2 片，安慰剂 2 片；阿立哌唑 20 mg 组：阿立哌唑 4 片；安慰剂组：安慰剂 4 片）。

研究期间，可给予苯二氮䓬类药物（氯硝西泮 2 ~4 $mg\cdot d^{-1}$或劳拉西泮 0.5 ~1.0 $mg\cdot d^{-1}$）改善睡眠障碍或焦虑；给予普萘洛尔 10 ~30 $mg\cdot d^{-1}$改善心动过速；给予苯海索 2 ~6 $mg\cdot d^{-1}$改善锥体外系症状。不得使用影响内分泌功能的药物。

4 量表评定

分别在基线和第 8 周末，采用 PANSS 评定精神症状，得分越高表明精神症状越严重。安全性评价采用 Barnes 静坐不能评定量表（Barnes Akathisia Scale，BAS）、Simpson-Angus 量表（Simpson Angus Scale，SAS）、UKU 副作用评定量表（UKU side effect rating scale，UKU），得分越高表明不良反应越严重。另外，采用 UKU 相关条目评定高催乳血症所致的性功能障碍（sexual dysfunction，SD）。

5 血样采集及检测

分别在基线、第 2，4 和 8 周末早晨 6∶30 ~7∶00（服用利培酮后 10 ~12 h）抽取空腹静脉血 10 mL，测定血清 PRL 水平（检测仪器为美国 Beckman-Coulter 公司生产的 ACCESS 型全自动微粒子化学发光分析仪）以及血生化、血常规。

6 疗效评定

客观指标包括 PRL 下降率[（基线 PRL 水平 - 8 周末 PRL 水平）/基线 PRL 水平 ×100%]和正常率（第 8 周末 PRL 水平≤24 $\mu g\cdot L^{-1}$的比率）。

7 统计学分析

用 EpiData 软件录入数据，采用 SPSS 13.0 软件包进行统计分析。计数资料用 $\bar{x} \pm s$ 描述。根据数据的性质，采用方差分析、χ^2 检验或重复测量方差分析。治疗 8 周末，统计 PRL 水平均数的 95% 可信区间（95% CI）。疗效分析用意向性分析（intention-

to-treat,ITT)人群。ITT 人群包括所有接受过至少 1 次研究药物治疗并且至少有 1 次基线后访视的受试者;安全性人群包括所有接受过至少 1 次研究药物的受试者。用末次观测值结转法(last observation carried forward,LOCF)对 ITT 人群进行分析,收集缺失的疗效数据。统计学分析均采用双侧检验,以 $P<0.05$ 为差异有统计学意义。

结　果

1　临床资料基本情况

2009 年 10 月 -2013 年 7 月,共纳入符合入组标准的患者 61 例。其中,阿立哌唑 5 mg 组 16 例、阿立哌唑 10 mg 组 15 例、阿立哌唑 20 mg 组 15 例、安慰剂组 15 例。研究期间脱落 6 例(9.8%)。其中,阿立哌唑 5 mg 组第 8 周失访 1 例;阿立哌唑 10 mg 组第 4 周失访 1 例;阿立哌唑 20 mg 组第 4 周因不良反应退出 1 例、8 周失访 1 例;安慰剂组第 4 周及 8 周失访各 1 例。4 组脱落率无统计学差异。

4 组在年龄、受教育年限、总病程、利培酮治疗时间、利培酮剂量以及基线 PRL 水平、基线 PANSS 评分、基线 CGI-S 评分方面无统计学显著差异,见表 1。

表 1　4 组人口学和一般资料比较　$\bar{x}\pm s$

项目	安慰剂组($n=15$)	阿立哌唑组 5 mg($n=16$)	阿立哌唑组 10 mg($n=15$)	阿立哌唑组 20 mg($n=15$)	F 值	P 值
年龄/岁	32.73 ±10.83	30.00 ±10.03	33.94 ±7.07	37.29 ±9.41	1.53	0.216
受教育年限/年	11.13 ±2.67	12.75 ±3.04	11.38 ±4.75	13.50 ±3.30	1.49	0.226
病程/年	10.53 ±7.10	9.11 ±7.86	12.66 ±5.20	12.04 ±8.22	0.78	0.511
利培酮治疗时间/月	9.40 ±3.68	8.44 ±2.42	10.38 ±4.81	8.57 ±3.20	0.94	0.427
利培酮剂量/mg·d^{-1}	5.07 ±1.10	4.50 ±1.27	4.88 ±1.15	5.21 ±1.12	1.08	0.366
基线 PRL/μg·L^{-1}	135.98 ±48.02	115.87 ±51.10	118.10 ±37.02	125.16 ±48.87	0.58	0.629
基线 PANSS/分	54.80 ±12.36	48.56 ±10.03	52.88 ±16.51	52.00 ±12.51	0.62	0.605

2　治疗前后 PRL 水平比较

重复测量方差分析发现,时间和分组的交互作用有统计学意义($F=15.22$,df = 9,$P<0.01$)。所有阿立哌唑剂量组治疗后第 2,4,8 周的 PRL 水平与基线水平有显著差异($P<0.01$),而第 2,4,8 周之间的 PRL 水平两两比较无显著性差异。安慰剂组 PRL 水平并未随治疗时间变化而明显改变。将年龄、利培酮剂量、利培酮治疗时间和病程作为协变量进行重复测量方法分析,时间和分组的交互作用仍具有统计学意义($F=14.25$,df = 9,$P<0.01$)。

治疗 8 周末,安慰剂组、阿立哌唑 5 mg 组、10 mg 组和 20 mg 组的 PRL 水平的 95% CI 分别为 109.24 ~157.97,41.98 ~87.95,27.05 ~44.06 和 20.10 ~40.87 μg·L^{-1}。方差分析显示,4 组之间 PRL 水平具有统计学显著差异($F=30.46$,df = 3,$P<0.001$)。其中,各阿立哌唑剂量组 PRL 水平均显著低于安慰剂组($P<0.01$),10 mg 组和 20 mg 组 PRL 水平显著低于 5 mg 组($P<0.05$),而 10 mg 组和 20 mg 组之间 PRL 水平无统计学差异。见表 2。

表 2　4 组治疗前后 PRL 水平比较　$\bar{x}\pm s$

PRL 水平	安慰剂组($n=15$)	阿立哌唑组 5 mg($n=16$)	阿立哌唑组 10 mg($n=15$)	阿立哌唑组 20 mg($n=15$)	F 值
基线/μg·L^{-1}	135.98 ±48.02	115.87 ±51.10	118.10 ±37.02	125.16 ±48.87	0.58
2 周末/μg·L^{-1}	135.78 ±49.84	68.42 ±36.61^{a}	43.41 ±16.25^{ab}	30.74 ±19.14^{ac}	29.22
4 周末/μg·L^{-1}	137.51 ±44.00	67.06 ±42.74^{a}	36.47 ±16.31^{ab}	30.53 ±15.67^{ac}	33.28
8 周末/μg·L^{-1}	133.60 ±44.00	64.97 ±43.14^{a}	35.56 ±15.96^{ab}	30.49 ±17.99^{ac}	30.46
0 ~8 周差值/μg·L^{-1}	2.37 ±17.38	50.90 ±40.11^{a}	82.54 ±41.94^{ab}	94.68 ±49.86^{ac}	16.70
8 周末降低率/%	-0.71 ±16.11	43.41 ±23.26^{a}	65.31 ±22.43^{ac}	72.93 ±16.11^{ac}	40.59
正常例数/n(%)	0(0)	1(6.25)	6(40.00)^{ac}	8(53.33)^{ac}	17.23

与安慰剂组比较,a:$P<0.01$;与阿立哌唑 5 mg 组比较,b:$P<0.05$,c:$P<0.01$

3 治疗终点高催乳素血症及相关症状的疗效比较

治疗 8 周末,4 组之间 PRL 降低率具有统计学差异($P<0.01$)。进一步分析显示,各阿立哌唑剂量组 PRL 降低率均显著高于安慰剂组($P<0.01$),10 mg 组和 20 mg 组 PRL 降低率均显著高于安慰剂组和 5 mg 组($P<0.01$),而 10 mg 组和 20 mg 组之间 PRL 降低率无统计学差异。见表 2。

研究终点,4 组之间 PRL 正常率有显著差异($P<0.01$)。进一步分析显示,阿立哌唑 10 mg 组和 20 mg 组 PRL 正常率均显著高于安慰剂组和5 mg 组($P<0.01$),而安慰剂组与 5 mg 组之间、10 mg 组与 20 mg 组之间 PRL 正常率无统计学差异。见表 2。

在基线水平,37 例患者出现 SD,包括闭经或月经量减少 32 例、泌乳 5 例、性欲减退 3 例和高潮功能障碍 3 例(个别患者出现 2 种及以上症状),4 组之间 SD 发生率无显著差异。8 周末,4 组之间 SD 发生率仍无显著差异。见表 3。

表 3 4 组治疗前后精神症状及药物不良反应变化

指标	安慰剂组($n=15$)	阿立哌唑组			F 值	P 值
		5 mg($n=16$)	10 mg($n=15$)	20 mg($n=15$)		
基线 PANSS	54.80 ± 12.36	48.56 ± 10.03	52.88 ± 16.51	52.00 ± 12.51	0.62	0.605
8 周末 PANSS	52.02 ± 12.97	48.25 ± 11.18	49.19 ± 13.18	46.42 ± 12.23	0.57	0.639
基线 SAS	2.20 ± 1.61	1.56 ± 1.50	1.50 ± 1.55	1.43 ± 2.03	0.68	0.571
8 周末 SAS	1.87 ± 1.64	1.31 ± 1.49	1.12 ± 1.63	0.86 ± 1.70	1.03	0.386
基线 BAS	0.40 ± 0.74	0.50 ± 0.82	0.38 ± 0.72	0.38 ± 0.63	0.12	0.939
8 周末 BAS	0.27 ± 0.46	0.44 ± 0.63	0.38 ± 0.72	0.21 ± 0.58	0.42	0.739
基线 SD 例数(%)	10(66.7)	10(62.5)	8(53.3)	9(60.0)	0.59	0.899
8 周末 SD 例数(%)	10(66.7)	7(50.0)	4(33.3)	5(40.0)	5.64	0.130

4 治疗前后精神症状、药物不良反应变化

4 组治疗前后 PANSS 评分、SAS 评分、BAS 评分均无统计学显著差异。治疗 8 周末,4 组 PANSS 评分、SAS 评分、BAS 评分均无统计学显著差异,见表 3。没有患者因病情波动退出研究。

在整个试验中出现不良反应 14 例,安慰剂组、阿立哌唑 5 mg 组、10 mg 组和 20 mg 组不良反应发生率分别为 20.0%,18.8%,20.0% 和 33.3%,无统计学显著差异。不良反应主要表现为轻度口干、便秘、恶心,无严重不良反应发生,仅阿立哌唑 20 mg 组 1 例患者因轻度恶心导致脱落。

讨　　论

PRL 的分泌受到下丘脑-垂体结节多巴胺神经元的直接抑制性调节,利培酮及其活性代谢产物 9-羟利培酮是较强的多巴胺 D_2 受体拮抗剂,可阻断这一通路的多巴胺受体,解除了对 PRL 的抑制,导致 PRL 水平上升[9]。阿立哌唑作为一种有效、高亲和性的 D_2 受体部分激动剂,它既可上调多巴胺功能的不足,又可下调多巴胺功能的亢进,是一种多巴胺递质的稳定剂[10]。另外,阿立哌唑也能原发性地抑制垂体前叶催乳素分泌[11]。由于阿立哌唑独特的药理机制,它不会升高或轻度降低 PRL 水平[1],且能有效改善利培酮所致高催乳素血症[2-5]。本研究发现,不同剂量的阿立哌唑均能显著改善利培酮所致的女性精神分裂症患者的高催乳素血症,并避免精神症状波动,不良反应发生率低,耐受性好,这与既往的研究结果一致[2-5]。另外,本研究表明随着 PRL 水平的下降,各阿立哌唑剂量组的 SD 例数下降,但与安慰剂组并未无显著差异,这可能与本研究样本量小、随访周期短有关。

近年来,有学者探讨阿立哌唑治疗利培酮所致高催乳素血症的最佳剂量。2013 年的一项荟萃分析报道[12],阿立哌唑在 5 mg·d^{-1}时已达到最大疗效。本课题组进行的一项阿立哌唑不同剂量、随机、双盲、安慰剂对照研究发现[6],阿立哌唑的治疗最佳剂量为 10 mg·d^{-1}。上述研究结果不一致的原因之一可能是未考虑到性别因素。文献报道[13],女性雌激素水平高,应用抗精神病药物治疗后,雌激素可加强多巴胺 D_2 受体阻断,从而增加 PRL 分泌细胞数量,提高 PRL 基因表达水平,进一步促进 PRL 分泌。因此,利培酮治疗的精神分裂症患者,女性 PRL

水平显著高于男性。在联用相同剂量的阿立哌唑时,其对利培酮所致女性精神分裂症患者高催乳素血症的疗效劣于男性[14]。因此,很有必要针对不同性别的患者分别展开研究,探讨阿立哌唑的最佳治疗剂量。

本研究发现,即使应用低剂量的阿立哌唑(5 $mg \cdot d^{-1}$),也能有效改善利培酮所致高催乳素血症。当阿立哌唑剂量增加到 10 $mg \cdot d^{-1}$时,PRL 水平降低率、高催乳素血症治愈率明显提高。而当阿立哌唑剂量高于 10 $mg \cdot d^{-1}$时,疗效鲜有增加。这提示 10 $mg \cdot d^{-1}$可能是阿立哌唑的最佳治疗剂量。Sparshatt 等[15]系统评估了阿立哌唑剂量、血浆浓度、药理作用、临床效果之间的关系。结果显示,阿立哌唑不同脑区的受体占有率没有明显差异,未观察到纹状体外脑区存在的优先结合。阿立哌唑血浆浓度和所有脑区的多巴胺受体占有率存在显著的相关性。当血浆水平高于 100 ~ 150 $ng \cdot mL^{-1}$时,D_2 受体占有率接近 100%。阿立哌唑血浆浓度 150 ~ 210 $ng \cdot mL^{-1}$对应的剂量范围为 10 ~ 15 $mg \cdot d^{-1}$。阿立哌唑高于 10 $mg \cdot d^{-1}$时,D_2 受体占有率已完全饱和。然而,本研究有诸多不足,例如研究时间相对短、样本量小,所以有待于进行长期的、大样本的随机双盲对照研究验证本研究结果。

[参 考 文 献]

[1] INDER WJ, CASTLE D. Antipsychotic-induced hyperprolactinaemia[J]. *Aust N Z J Psychiatry*, 2011,45(10):830 - 837.

[2] CHEN JX, SU YA, BIAN QT, *et al.* Aripiprazole treatment of risperidone-induced hyperprolactinemia [J]. *J Clin Psychiat*, 2009, 70(7): 1058 - 1059.

[3] 陈景旭, 张荣珍, 李伟, 等. 阿立哌唑治疗利培酮所致高催乳素血症的双盲对照研究[J]. 中国新药杂志, 2014, 23(7): 811 - 814.

[4] ZHAO J, SONG X, AI X, *et al.* Adjunctive aripiprazole treatment for risperidone-induced hyperprolactinemia: an 8-week randomized, open-label, comparative clinical trial[J]. *PLoS One*, 2015,10(10): e0139717.

[5] RANJBAR F, SADEGHI-BAZARGANI H, NIARI KHAMS P, *et al.* Adjunctive treatment with aripiprazole for risperidone-induced hyperprolactinemia[J]. *Neuropsychiatr Dis Treat*, 2015, 11:549 - 555.

[6] CHEN JX, SU YA, QING TB, *et al.* Adjunctive aripiprazole in the treatment ofrisperidone-induced hyperprolactinemia: a randomized, double-blind, placebo-controlled, dose-response study [J]. *Psychoneuroendocrinology*, 2015, 58:130 - 140.

[7] SUZUKI Y, FUKUI N, WATANABE J, *et al.* Gender differences in the relationship between the risperidone metabolism and the plasma prolactin levels in psychiatric patients[J]. *Prog Neuropsychopharmacol Biol Psychiatry*, 2010, 34(7):1266 - 1268.

[8] NAGAI G, MIHARA K, NAKAMURA A. Prolactin concentrations during aripiprazole treatment in relation to sex, plasma drugs concentrations and genetic polymorphisms of dopamine D2 receptor and cytochrome P450 2D6 in Japanese patients with schizophrenia [J]. *Psychiatry Clin Neurosci*, 2012, 66 (6): 518 - 524.

[9] 苏允爱,司天梅,舒良. 抗精神病药与高催乳素血症[J]. 国外医学精神病学分册, 2003, 30(3):160 - 163.

[10] KESSLER RM. Aripiprazole: what is the role of dopamine D2 receptor partial agonism? [J]. *Am J Psychiatry*, 2007, 164(9): 1310 - 1312.

[11] INOUE T, DOMAE M, YAMADA K, *et al.* Effects of the novel antipsychotic agent 7-[4-[(2,3-dichlorophenyl)-1-piperazinyl] butyoxy]-3, 4-dihydro-2 (1*H*)-quinolinone (OPC-14597) on prolactin release from the rat anterior pituitary gland[J]. *J Pharmacol Exp Ther*, 1996, 277(1):137 - 143.

[12] LI X, TANG Y, WANG C. Adjunctive aripiprazole versusplacebo for antipsychotic-induced hyperprolactinemia: meta-analysis of randomized controlled trials [J]. *PLoS One*, 2013, 8 (8): e70179.

[13] 喻东山, 余琳. 精神药物和高催乳素血症[J]. 中国新药与临床杂志, 2004, 23(12): 893 - 896.

[14] 陈景旭, 梁雪梅, 卞清涛, 等. 阿立哌唑治疗利培酮所致高催乳素血症效果的性别差异[J]. 四川精神卫生, 2015, 28(3): 215 - 218.

[15] SPARSHATT A, TAYLOR D, PATEL MX, *et al.* A systematic review of aripiprazole-dose, plasma concentration, receptoroccupancy, and response: implications for therapeutic drug mon-itoring[J]. *J Clin Psychiatry*, 2010, 71(11): 1447 - 1456.

编辑:罗娟/接受日期:2015 - 12 - 26

★ **FDA 批准 Briviact 用于治疗部分性癫痫发作** 美国 FDA 近日批准 Briviact(布瓦西坦)作为其他药物的添加治疗用药用于 16 岁及以上癫痫患者的部分性发作。

导致癫痫发作的可能原因很多,包括卒中、感染、肿瘤、创伤性脑损伤以及脑部发育异常。在许多情况下,具体原因并不清楚。三项临床试验对 Briviact 的有效性进行了评价,共计纳入 1550 例受试者。FDA 药品评价与研究中心神经病学产品室主任 Billy Dunn 博士称:"患者对现有抗癫痫药物应答情况不同,我高兴地看到,Briviact 的获批为癫痫患者提供了新的治疗药物选择。"Briviact 与其他药物联合应用,可有效减少癫痫发作频次。临床试验中服用 Briviact 的受试者报告的最常见不良反应包括困倦、头晕、疲劳、恶心及呕吐。患者罕见唇部、眼睑或舌部水肿以及伴有或不伴有呼吸困难等过敏反应。Briviact 由乔治亚州士麦那(Smyrna)的优时比(UCB)制药公司上市销售。医生必须将 Briviact 与用药指南一同发放给患者,该指南提供了药物应用和风险的相关重要信息(来源:FDA,2016 - 02 - 20)。
