# Supplementary material for: Dose-Dependent Efficacy of Aripiprazole in Treating Patients With Schizophrenia or Schizoaffective Disorder: A Systematic Review and Meta-Analysis of Randomized Controlled Trials
Source: Front Psychiatry. 2021 Aug 11;12:717715. doi: 10.3389/fpsyt.2021.717715 (PMC8385236; doi:10.3389/fpsyt.2021.717715)
Supplement: Supplementary file 1 [file Data_Sheet_1.ZIP › supplementray material-9 RCTs/1 Kane 2002.pdf]

Efficacy and Safety of Aripiprazole and Haloperidol Versus Placebo in Patients With Schizophrenia and Schizoaffective Disorder

John M. Kane, M.D.; William H. Carson, M.D.; Anutosh R. Saha, Ph.D.; Robert D. McQuade, Ph.D.; Gary G. Ingenito, M.D.; Dan L. Zimbroff, M.D.; and Mirza W. Ali, Ph.D.

© Copyright 2002 Physicians Postgraduate Press, Inc.
One personal copy may be printed

Background: Aripiprazole is an investigational agent for treating schizophrenia that has a novel pharmacologic profile. The present study investigated the efficacy, safety, and tolerability of aripiprazole and haloperidol compared with placebo.

Method: A 4-week, double-blind, randomized study, conducted at 36 U.S. centers between July 1997 and June 1998, compared aripiprazole (15 mg/day, 30 mg/day) to placebo, with haloperidol (10 mg/day) as an active control. Fixed doses of each agent were administered from day 1 throughout the study. A total of 414 patients with a primary DSM-IV diagnosis of schizophrenia or schizoaffective disorder were randomized. Efficacy measures included the Positive and Negative Syndrome Scale (PANSS) total, PANSS positive, PANSS negative, PANSS-derived Brief Psychiatric Rating Scale (BPRS) core, Clinical Global Impressions (CGI)-Severity of Illness, and mean CGI-Improvement scores. Safety and tolerability evaluations included extrapyramidal symptoms (EPS), weight gain, serum prolactin level, and QTc interval.

Results: Both doses of aripiprazole and haloperidol, 10 mg, produced statistically significant ($p \le .05$) improvements from baseline in PANSS total, PANSS positive, PANSS-derived BPRS core, and CGI-Severity scores and significantly lower CGI-Improvement scores at endpoint, compared with placebo. Aripiprazole, 15 mg, and haloperidol, 10 mg, significantly improved PANSS negative score compared with placebo. Both aripiprazole doses and haloperidol separated from placebo for PANSS total scores at week 2. Unlike haloperidol, aripiprazole was not associated with significant EPS or prolactin elevation at endpoint compared with placebo. There were no statistically significant differences in mean changes in body weight across the treatment groups versus placebo, and no patients receiving aripiprazole experienced clinically significant increases in QTc interval.

Conclusion: Aripiprazole, effective against positive and negative symptoms, is a safe and well-tolerated potential treatment for schizophrenia and schizoaffective disorder.

(J Clin Psychiatry 2002;63:763–771)

Received Feb. 4, 2002; accepted July 8, 2002. *From the Zucker Hillside Hospital, Glen Oaks, N.Y. (Dr. Kane); Bristol-Myers Squibb, Wallingford, Conn. (Dr. Carson); Otsuka Maryland Research Institute, Rockville, Md. (Drs. Saha, Ingenito, and Ali); Bristol-Myers Squibb, Lawrenceville, N.J. (Dr. McQuade); and Pacific Clinical Research, San Bernardino, Calif. (Dr. Zimbroff). Dr. Carson is now with Otsuka America Pharmaceutical, Princeton, N.J.*

This study was supported by Otsuka Pharmaceutical Co., Ltd. (Tokyo, Japan) and Bristol-Myers Squibb Company (Princeton, N.J.).

Dr. Kane has been a consultant for and/or received honoraria from Bristol-Myers Squibb, Pfizer, Abbott, Janssen, Novartis, Lundbeck, AstraZeneca, and Lilly.

Members of the Investigator Study Group are listed at the end of the article.

Corresponding author and reprints: Robert D. McQuade, Ph.D., Bristol-Myers Squibb Company, Route 206 & Province Line Road, Princeton, NJ 08543 (e-mail: robert.mcquade@bms.com).

Schizophrenia is among the most serious of mental illnesses; it causes great distress to patients and their families and has a considerable social and economic impact. Globally, it affects approximately 1% of the population and is a major cause of disability.[1,2] In the United States, alone, where schizophrenia affects more than approximately 2.5 million people, it costs an estimated $40 billion a year including lost productivity.[2]

Antipsychotic medication is the main therapeutic intervention for schizophrenia. Although the pathophysiology of the disease has yet to be clearly defined, antipsychotic drug development has been heavily influenced by the dopamine hypothesis,[3] which states that dopamine overactivity in the brain is responsible for the disease. Evidence for this hypothesis includes the capacity of antipsychotic drugs to block dopamine receptors in vivo[4] and in vitro.[5] In addition, the clinical efficacy of antipsychotic drugs is highly correlated with their ability to block dopamine D_2 receptors.[6,7]

The early agents for the treatment of psychosis, the "typical" antipsychotics, were breakthrough therapies for the positive symptoms of schizophrenia but were less effective in treating the negative symptoms of the disease. In addition, the D_2 receptor antagonism of these drugs produced unwanted side effects, such as extrapyramidal symptoms (EPS) and hyperprolactinemia.[8]

The "atypical" antipsychotics, introduced in the mid-1990s, combined D_2 blockade with antagonism of seroto-

nin 5-HT_{2A} receptors and produced significantly less EPS and hyperprolactinemia than the typical antipsychotics. However, different side effects have been seen with individual agents, including weight gain and somnolence, that can decrease compliance and persistency,[9,10] leading to an increased risk of relapse. Thus, there is still an unmet need for novel antipsychotic drugs that are better tolerated than earlier typical agents and currently available atypical medications. In addition, enhanced overall efficacy against the positive symptoms, negative symptoms, and cognitive dysfunction of schizophrenia remains an unmet medical need.

In attempts to meet these unmet needs, researchers have explored the possibility of using dopamine D_2 partial agonists for the treatment of schizophrenia.[11–13] The partial dopamine D_2 receptor agonist (–)-3-(3-hydroxyphenyl)-N-n-propylpiperidine (–3-PPP; preclamol) is perhaps the best studied of these agents. In a recent clinical study,[11] preclamol, 300 mg b.i.d., decreased the positive and negative symptoms of schizophrenia relative to placebo. However, these antipsychotic actions were not sustained for longer than 1 week.

Aripiprazole is a novel agent with a unique pharmacologic profile that acts as a potent partial agonist at dopamine D_2 receptors, a partial agonist at serotonin 5-HT_{1A} receptors, and an antagonist at 5-HT_{2A} receptors.[14–16] It has been suggested that dopamine partial agonists may be capable of stabilizing the dopaminergic system without inducing the hypodopaminergia that limits the tolerability of currently available antipsychotics.[17–19] Aripiprazole acts as a functional antagonist at D_2 receptors under hyperdopaminergic conditions, but exhibits functional agonist properties under hypodopaminergic conditions. In an animal model of dopaminergic hyperactivity, aripiprazole blocked apomorphine-induced stereotypy in a dose-dependent manner, comparable to the action of haloperidol, a D_2 antagonist.[20] In contrast, in an animal model of dopaminergic hypoactivity, aripiprazole dose-dependently attenuated reserpine-induced dopa accumulation via stimulation of presynaptic D_2 receptors,[20] and this effect was reversed by haloperidol. Dopamine partial agonist effects of aripiprazole were also observed in a study in which aripiprazole inhibited spontaneous prolactin release from rat pituitary slices; this inhibition was less than that produced by the dopamine agonist talipexole and was completely antagonized by haloperidol.[21] This pharmacodynamic profile is consistent with dopamine partial agonist activity at D_2 dopamine receptors, which is believed to result in stabilization of the dopamine system.

In addition to dopamine D_2 partial agonism, aripiprazole acts as a partial agonist at some 5-HT receptor subtypes and as an antagonist at others. At 5-HT_{1A} receptors, aripiprazole is a partial agonist.[15] The clinical relevance of aripiprazole's partial agonism at 5-HT_{1A} receptors warrants further investigation; 5-HT_{1A} partial agonists such as buspirone are known to have anxiolytic efficacy[22] and have been shown to reduce stress-induced psychosocial deficits in patients with chronic schizophrenia.[23,24] Putative relationships have also been postulated between 5-HT_{1A} agonism and improvement in depression, cognition, and negative symptoms.[25] Preclinical studies have also demonstrated that aripiprazole is an antagonist at 5-HT_{2A} receptors.[16] Antagonism at 5-HT_{2A} receptors may confer a favorable effect on negative symptoms[25–27] and have an association with low EPS liability.[8]

In combination, the above studies suggest that aripiprazole may function as a dopamine-serotonin system stabilizer, an agent that acts as a functional antagonist or functional agonist at dopamine and serotonin receptors depending on the level of the relevant neurotransmitter in the immediate environment. The activity of aripiprazole at dopamine and serotonin receptors suggests that the drug may have overall efficacy against the symptoms of schizophrenia, including both positive and negative symptoms, with a low risk of side effects. Placebo-controlled trials are necessary to determine whether these potential benefits are translated into quantifiable clinical effects.

This multicenter, randomized, double-blind, placebo-controlled study was conducted to evaluate the efficacy and safety of 2 doses of aripiprazole (15 mg and 30 mg per day) and haloperidol (10 mg per day) for the treatment of patients with acute relapse of schizophrenia or schizoaffective disorder.

METHOD

Inclusion criteria required that participants were males and nonpregnant, nonlactating females using suitable contraceptive measures, aged 18 to 65 years, with a primary diagnosis of schizophrenia or schizoaffective disorder (DSM-IV criteria), who were hospitalized for an acute relapse (DSM-IV). The diagnostic evaluation included the following: psychiatric evaluation (DSM-IV diagnosis of schizophrenia or schizoaffective disorder, acute relapse, history of the disease, and history of response to treatment), a general clinical evaluation (including medical history, physical examination, and current symptoms), medication history during past week, Positive and Negative Syndrome Scale (PANSS)[28] and Clinical Global Impressions (CGI) scale[29] scores, demographics, as well as vital signs, body weight, electrocardiogram (ECG), and laboratory tests. Patients taking a long-acting antipsychotic underwent an appropriate washout period (time required for 1 cycle of treatment plus 1 week), unless they were deemed clinically deteriorating in the investigator's judgment, in which case they could be enrolled within less than the specified time period. Results of serum chemistry analysis at screening were reviewed before randomly assigning these patients to study medication. In

© Copyright 2002 Physicians Postgraduate Press, Inc. One personal copy may be printed

addition, patients were to have a PANSS total score of at least 60 and scores of at least 4 (moderate) on any 2 of the items on the psychotic items subscale (hallucination, delusion, conceptual disorganization, and suspiciousness). They also had to meet the following requirements for prior responsiveness to antipsychotic medication, that is: previously diagnosed with schizophrenia or schizoaffective disorder, not refractory to antipsychotics and had improvement produced by an antipsychotic agent other than clozapine, and had been an outpatient for at least one 3-month period during the past year.

Patients were excluded from the study if they had a psychiatric disorder other than schizophrenia or schizoaffective disorder, a history of violence, a history of suicidal attempts or serious suicidal ideation, a clinically significant neurologic abnormality other than tardive dyskinesia or EPS, psychoactive drug abuse or dependence, drug or alcohol abuse, or treatment with an investigational drug within 4 weeks prior to the washout phase. Patients were also excluded if they had any other acute or unstable medical condition. After complete description of the study to the subjects, written informed consent was obtained, and cosigned by their next of kin or caregiver if required by the local institutional review board.

This randomized, double-blind, placebo-controlled, parallel-group, 4-week study was conducted at 36 centers in the United States between July 1997 and June 1998. Patients were screened for inclusion into the study at the initial visit and then underwent a minimum 5-day placebo washout period starting within 1 week of the screening visit. Additionally, at screening the following data were collected: age at first episode, number of previous hospitalizations, and prior antipsychotic use. Investigators had access to the medical history of the patients at the screening. After washout, patients were evaluated for entry into the treatment period of the study. Patients were included if their PANSS total score still met the inclusion criteria and they did not have any of the following: diagnosis with a psychiatric disorder other than schizophrenia or schizoaffective disorder during the washout period, a clinically significant abnormal laboratory value, or any acute or unstable medical condition.

Patients were randomly assigned to 1 of 4 treatment groups: 15 mg of aripiprazole once daily; 30 mg of aripiprazole once daily; 10 mg of haloperidol once daily; or placebo. Treatments were given orally after breakfast, and patients were treated for 4 weeks. Doses of aripiprazole and haloperidol were fixed throughout the study. Patients were hospitalized for the entire duration of the study (washout period and double-blind treatment).

Treatment efficacy was assessed using the PANSS and CGI scale. The PANSS evaluation included the total score (30 items), the positive subscale (7 items), and the negative subscale (7 items). Symptom severity was rated using a 7-point scale. The CGI consisted of two 7-point scales: Severity of Illness scale (CGI-S) and Global Improvement scale (CGI-I). For each patient, the same rater conducted the assessment throughout the study and was blinded to the patient's treatment. To ensure interrater reliability, specific procedures were used. Rater training was conducted at the investigator meeting by an experienced trainer and via videotape(s) for study center staff not present at the meeting. The PANSS training session included viewing actual patient interviews, discussion of the cases, and scoring by the designated raters from participant study centers. Rating forms were collected and analyzed by the PANSS trainer. Each attendee's rating score was then compared with the overall consensus score of the group. All raters were required to successfully complete rater training and be approved by the sponsor prior to rating patients. Efficacy assessments were performed at screening, at the end of the placebo washout period (baseline), and the end of each week of treatment (days 7, 14, 21, and 28). Primary efficacy variables were mean change from baseline to week 4 in PANSS total, PANSS positive subscale, and CGI-S scores. Other efficacy variables evaluated included mean change from baseline in PANSS negative subscale and PANSS-derived Brief Psychiatric Rating Scale (BPRS) core scores, mean CGI-I score, and responder rates (patients with a CGI-I score of 1 or 2 or a ≥ 30% decrease from baseline in PANSS total score were considered responders). Adverse events were monitored at the end of the placebo washout period (baseline) and weekly throughout the study. Investigators graded the intensity of any adverse events and assessed their likely relationship to the study medication. The status and intensity of previously reported adverse events were also evaluated at each weekly assessment.

EPS were evaluated at baseline and weekly throughout the study using the Simpson-Angus Scale,[30] Barnes Akathisia Scale,[31] and the Abnormal Involuntary Movement Scale (AIMS).[32]

Vital signs were monitored at screening, baseline, and on days 14 and 28. Additional measurements were also made on days 1 to 5 of treatment, both before dosing and 4 to 6 hours after dosing. Twelve-lead ECGs, blood samples, and urinalysis were assessed at screening, baseline (blood sample collection only), and on days 14 and 28.

Use of psychotropic agents (other than the study medication) was prohibited throughout the washout and treatment periods of the study, except for lorazepam for anxiety or insomnia. Lorazepam, administered intramuscularly, was also permitted for emerging agitation. Benztropine treatment was allowed for EPS, if judged necessary by the investigator. The dose was limited to a maximum of 6 mg per day, and was only permitted during the treatment phase of the study.

Analysis of efficacy parameters was performed on an intent-to-treat (ITT) basis using data obtained from each patient's last visit (i.e., last observation carried forward

© Copyright 2002 Physicians Postgraduate Press, Inc. One personal copy may be printed

Table 1. Baseline Demographic Characteristics, Including Efficacy and Safety Parameters (all patients)[a]

Characteristic	Placebo (N = 106)	Aripiprazole, 15 mg (N = 102)	Aripiprazole, 30 mg (N = 102)	Haloperidol, 10 mg (N = 104)	Total (N = 414)
Male/female (N)	74/32	76/26	70/32	68/36	288/126
Age, mean ± SE, y	38.5 ± 0.9	37.8 ± 1.0	39.3 ± 1.0	38.9 ± 0.9	38.6 ± 0.5
Body weight, mean ± SE, kg	83.3 ± 2.1	85.3 ± 2.2	87.8 ± 2.4	84.8 ± 2.0	85.3 ± 1.1
Serum prolactin level,[b] mean, ng/mL	11.2	12.4	12.3	15.7	12.9
Patients with schizophrenia/ schizoaffective disorder (N)	75/31	74/28	72/30	61/43	282/132
Age at first episode, mean ± SE, y	22.5 ± 0.7	21.8 ± 0.8	22.1 ± 0.7	22.9 ± 0.7	22.3 ± 0.4
PANSS total score, mean ± SE	100.2 ± 1.6	98.5 ± 1.7	99.0 ± 1.9	99.3 ± 1.7	99.3 ± 0.9
PANSS positive subscale score, mean ± SE	25.0 ± 0.5	24.8 ± 0.5	24.5 ± 0.5	25.2 ± 0.5	24.9 ± 0.3
PANSS negative subscale score, mean ± SE	25.8 ± 0.6	25.1 ± 0.6	25.5 ± 0.6	25.6 ± 0.7	25.5 ± 0.3
CGI-Severity of Illness score, mean ± SE	4.9 ± 0.1	4.9 ± 0.1	4.8 ± 0.1	4.8 ± 0.1	4.9 ± 0.0
No. of previous hospitalizations, mean ± SE	11.1 ± 1.5	8.4 ± 1.3	10.8 ± 1.8	9.8 ± 1.4	10.0 ± 0.8
Prior antipsychotics used, %					
Olanzapine	30	35	29	32	
Risperidone	21	20	23	35	
Haloperidol	26	25	28	23	
Other typical	44	40	33	40	
Other atypical	6	9	7	10	

[a]Abbreviations: CGI = Clinical Global Impressions, PANSS = Positive and Negative Syndrome Scale.
[b]The mean serum prolactin level was calculated for each group based on the following numbers of patients: placebo, N = 96; aripiprazole, 15 mg, N = 90; aripiprazole, 30 mg, N = 89; haloperidol, 10 mg, N = 95; and total, N = 370.

Table 2. Discontinuation Rates in Patients Randomly Assigned to Treatment

Variable	Placebo (N = 106)		Aripiprazole, 15 mg (N = 102)		Aripiprazole, 30 mg (N = 102)		Haloperidol, 10 mg (N = 104)		Total (N = 414)	
	N	%	N	%	N	%	N	%	N	%
Completed study	58	55	68	67	60	59	62	60	248	60
Discontinued	48	45	34	33	42	41	42	40	166	40
Reasons for discontinuation										
Adverse event	17	16	9	9	8	8	11	11	45	11
Withdrew consent	13	12	19	19	16	16	24	23	72	17
Insufficient clinical response	15	14	5	5	15	15	6	6	41	10
Other reasons[a]	3	3	1	1	3	3	1	1	8	2

[a]Includes patients who were lost to follow-up, noncompliant, or met withdrawal criteria.

© Copyright 2002 Physicians Postgraduate Press, Inc.
One personal copy may be printed

[LOCF] analysis at week 4). The ITT population consisted of all patients with at least 1 baseline and postbaseline evaluation. Treatment comparisons were aripiprazole 30 mg versus placebo, aripiprazole 15 mg versus placebo, and haloperidol versus placebo. Primary efficacy parameters were evaluated by analysis of covariance (ANCOVA) adjusting for baseline values and study center.

Other efficacy variables were analyzed in the same manner as primary efficacy variables, with the exception of CGI-I and responder analysis, which used the Cochran-Mantel-Haenszel test stratified by center.

Analyses of EPS scales, prolactin level, body weight, and QTc interval were performed using ANCOVA.

Statistical significance versus placebo was reached if the p value was less than or equal to .05.

RESULTS

Patients

Of the 502 patients enrolled at baseline, 414 were randomly assigned to double-blind treatment. Baseline characteristics for randomized patients are listed in Table 1. Of the 414 randomized patients, 248 completed the 4-week study period. Reasons for discontinuation are given in Table 2.

Efficacy Results

Both doses of aripiprazole produced significant improvements compared with placebo in the following measures: PANSS total score, PANSS positive subscale score, CGI-S score, CGI-I score, and PANSS-derived BPRS

Table 3. Efficacy Results (last-observation-carried forward [LOCF] analysis)[a]

Measure	Placebo	Aripiprazole, 15 mg	p Value vs Placebo	Aripiprazole, 30 mg	p Value vs Placebo	Haloperidol, 10 mg	p Value vs Placebo
PANSS total[b]	–2.9	–15.5	< .001	–11.4	.009	–13.8	.001
PANSS positive[b]	–0.6	–4.2	< .001	–3.8	.001	–4.4	< .001
PANSS negative[b]	–1.2	–3.6	.006	–2.3	.213	–2.9	.043
PANSS-derived BPRS core[b]	–1.1	–3.1	.001	–3.0	.001	–3.5	< .001
CGI-Severity[b]	–0.1	–0.6	< .001	–0.4	.019	–0.5	.002
CGI-Improvement[c]	4.3	3.5	< .001	3.8	.016	3.7	.002
Responder rate, %	17	35	.002	28	.050	26	.089

[a]Abbreviations: BPRS = Brief Psychiatric Rating Scale, CGI = Clinical Global Impressions, PANSS = Positive and Negative Syndrome Scale.
[b]Mean change in values from baseline.
[c]Mean values.

© Copyright 2002 Physicians Postgraduate Press, Inc.
One personal copy may be printed

Figure 1. Mean Change in PANSS Total Score From Baseline Over 4 Weeks of Treatment With Aripiprazole (15 mg or 30 mg), Haloperidol 10 mg, or Placebo (LOCF)[a,b]

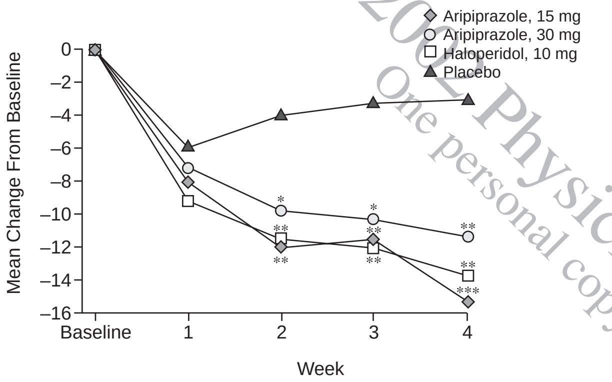

[a]Abbreviations: LOCF = last observation carried forward, PANSS = Positive and Negative Syndrome Scale.
[b]Pairwise comparison p values (vs. placebo): *p < .05; **p < .01; ***p < .001.

Figure 2. Mean Change in PANSS Positive Subscale Score From Baseline Over 4 Weeks of Treatment With Aripiprazole (15 mg or 30 mg), Haloperidol 10 mg, or Placebo (LOCF)[a,b]

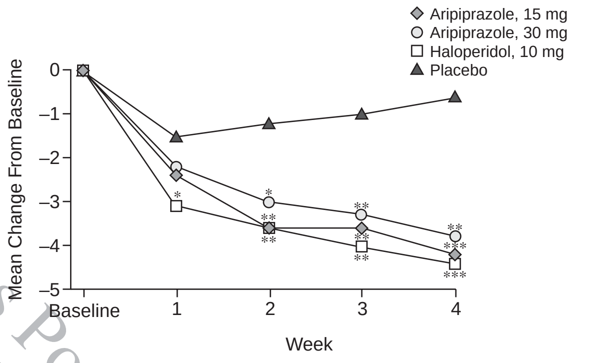

[a]Abbreviations: LOCF = last observation carried forward, PANSS = Positive and Negative Syndrome Scale.
[b]Pairwise comparison p values (vs. placebo): *p < .05; **p < .01; ***p < .001.

core score (Table 3; Figures 1–3). Aripiprazole 15 mg also produced a significantly greater improvement in PANSS negative subscale score compared with placebo (p = .006); aripiprazole 30 mg produced an improvement compared with placebo, although this was not statistically significant (Table 3). All active treatment groups demonstrated similarly rapid onset of efficacy, with both doses of aripiprazole and haloperidol 10 mg separating from placebo by week 2 for PANSS total score. The haloperidol group also showed significantly greater improvement in all these efficacy measures than placebo.

Responder rates for aripiprazole-treated patients were greater than for placebo, but there was no significant difference between the responder rates for haloperidol and those for placebo (Table 3).

Safety

Adverse events. Aripiprazole treatment was well tolerated, with adverse events generally mild-to-moderate in intensity and not treatment-limiting. Overall, 45 (11%) of the 414 randomized patients discontinued from the study due to an adverse event; 17 patients (16%) in the placebo group, 11 patients (11%) in the haloperidol group, 9 patients (9%) in the aripiprazole 15-mg group, and 8 patients (8%) in the aripiprazole 30-mg group. The most frequent adverse event that led to discontinuation was worsening of psychosis, which, as expected, was higher in the placebo group. The most common treatment-emergent adverse events, i.e., those occurring at an incidence of 5% or more in at least 1 treatment group are shown in Table 4. The occurrence of somnolence in 13% (13/103) of patients in the haloperidol 10-mg group was consistent with the known potential of the drug to cause sedation. The incidence of somnolence in the other treatment groups was as follows: placebo: 4%; aripiprazole 15 mg: 5%; and aripiprazole 30 mg: 10%. Nausea was reported by 7% of patients in the placebo group, 6% of patients in the haloperidol group, 15% of patients in the aripiprazole 15-mg

Figure 3. Mean Change in CGI-S Score From Baseline Over 4 Weeks of Treatment With Aripiprazole (15 mg or 30 mg), Haloperidol 10 mg, or Placebo (LOCF)[a,b]

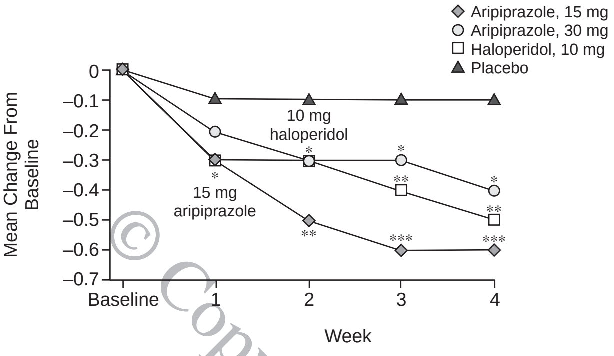

[a]Abbreviations: CGI-S = Clinical Global Impressions-Severity of Illness scale, LOCF = last observation carried forward.
[b]Pairwise comparison p values (vs. placebo): *p < .05; **p < .01; ***p < .001.

Figure 4. Mean Change From Baseline to Last Visit for Simpson-Angus Scale, Barnes Akathisia Scale, and AIMS Total Score for All Treatment Groups (LOCF)[a,b]

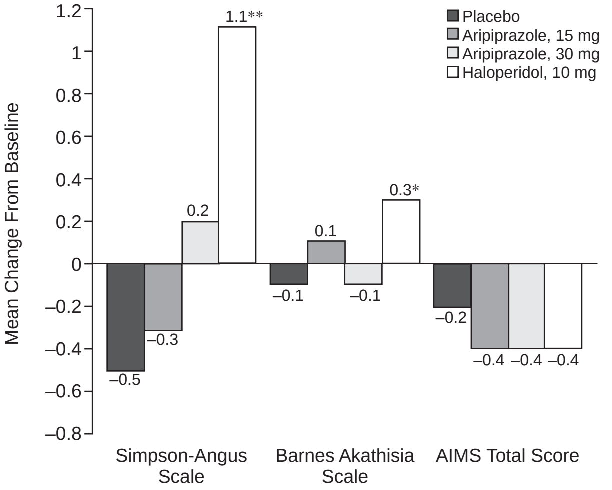

[a]Abbreviations: AIMS = Abnormal Involuntary Movement Scale, LOCF = last observation carried forward.
[b]Pairwise comparison p values (vs. placebo): *p < .05; **p < .001.

Table 4. Incidence of Treatment-Emergent Adverse Events (≥ 5% in any treatment group)

	Placebo (N = 104)		Aripiprazole, 15 mg (N = 102)		Aripiprazole, 30 mg (N = 101)		Haloperidol, 10 mg (N = 103)	
Adverse Event	N	%	N	%	N	%	N	%
Headache	24	23	24	24	29	29	26	25
Anxiety	16	15	23	23	17	17	20	19
Insomnia	18	17	19	19	22	22	25	24
Nausea	7	7	15	15	14	14	6	6
Dizziness	6	6	13	13	17	17	6	6
Abdominal pain	5	5	9	9	6	6	6	6
Vomiting	10	10	8	8	17	17	10	10
Akathisia	11	11	8	8	12	12	24	23
Somnolence	4	4	5	5	10	10	13	13
Asthenia	3	3	3	3	6	6	5	5
Orthostatic hypotension	3	3	2	2	7	7	1	1
Hypertonia	5	5	2	2	8	8	3	3
Tremor	3	3	2	2	3	3	7	7
Blurred vision	1	1	1	1	2	2	8	8

group, and 14% of patients in the aripiprazole 30-mg group. Vomiting was reported by 10% of patients in the placebo and haloperidol groups, 8% of patients in the aripiprazole 15-mg group, and 17% of patients in the aripiprazole 30-mg group. No patients in any group discontinued due to nausea or vomiting. In patients receiving aripiprazole, the majority of reports of nausea and vomiting were mild in intensity; most reports of nausea and vomiting occurred during the first week of the study and resolved within 1 week.

Overall, 15 patients (3.7%) experienced serious adverse events during the study (aripiprazole 15 mg, N = 4; aripiprazole 30 mg, N = 2; haloperidol, N = 6; placebo, N = 3). Most events were related to the underlying diagnosis, with psychosis the most frequent (9 reports overall).

Extrapyramidal symptoms. The overall incidence of EPS-related adverse events in the aripiprazole groups was comparable to placebo and less than haloperidol. Eighteen patients (18%) in the aripiprazole 15-mg group and 20 patients (20%) in the aripiprazole 30-mg group reported an EPS-related adverse event, compared with 37 patients (36%) in the haloperidol group and 22 patients (21%) in the placebo group. A greater percentage of patients in the haloperidol 10-mg group required benztropine for EPS (30%) compared with other groups (12% for the placebo group, 8% for the aripiprazole 15-mg group, and 15% for aripiprazole 30-mg group). Aripiprazole was not associated with significant changes in Simpson-Angus Scale, Barnes Akathisia, or AIMS scores compared with placebo (Figure 4).

Body weight. Comparison of mean change in body weight across the treatment groups demonstrated no significant differences from placebo. Mean weight changes from baseline to last visit for all treatment groups are shown in Figure 5A; the incidence of clinically significant weight gain (≥ 7% increase from baseline) is shown in Figure 5B.

Serum prolactin levels. Serum prolactin levels decreased from baseline in both aripiprazole groups and the placebo group over the course of the study. The changes in the aripiprazole groups were not statistically significantly different from the change in the placebo group, whereas haloperidol 10 mg produced an increase in mean serum prolactin level that was statistically significant compared with the small decrease observed in the placebo group ($p < .001$) (Figure 6).

ECG. No significantly different changes in QTc interval (as calculated by the Bazett's conversion formula, $QTc_B = QT/RR^{0.5}$) from placebo were seen in the treatment

© Copyright 2002 Physicians Postgraduate Press, Inc.
One personal copy may be printed

Figure 5A. Mean Change in Body Weight From Baseline to Last Visit for All Treatment Groups (LOCF)[a]

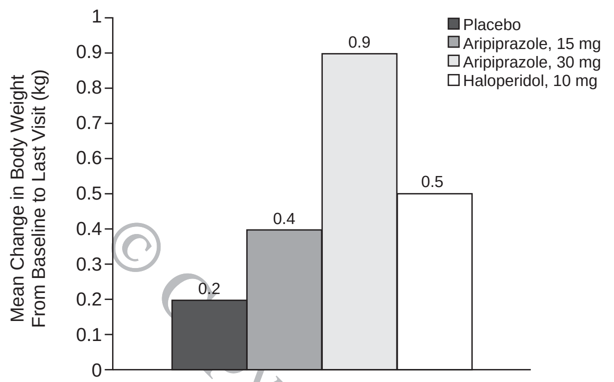

Figure 5B. Incidence of Clinically Significant Weight Gain (≥ 7% increase from baseline) at Endpoint for All Treatment Groups (LOCF)[b]

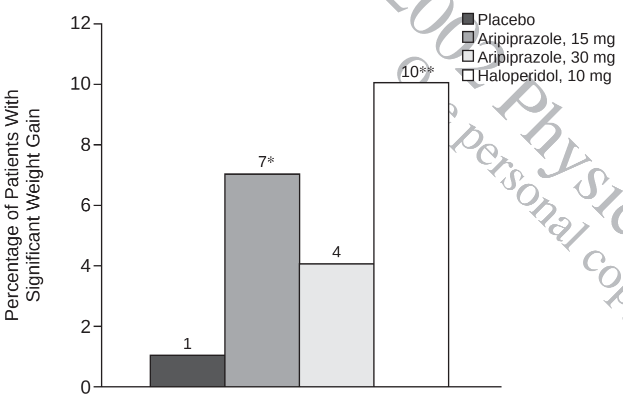

[a]Abbreviation: LOCF = last observation carried forward.
[b]Pairwise comparison p values (vs. placebo): *p < .05; **p < .01.

Figure 6. Mean Change in Prolactin Levels From Baseline to Last Visit for All Treatment Groups (LOCF)[a,b]

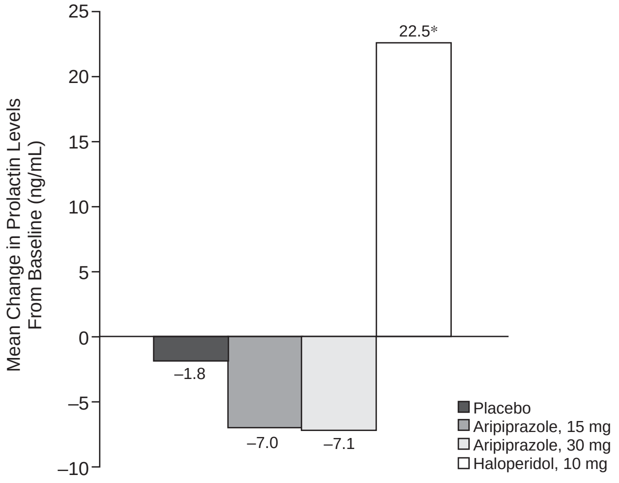

[a]Abbreviation: LOCF = last observation carried forward.
[b]Pairwise comparison p values (vs. placebo): *p < .001.

Figure 7. Mean Change in QTc Interval From Baseline to Last Visit for All Treatment Groups (LOCF)[a]

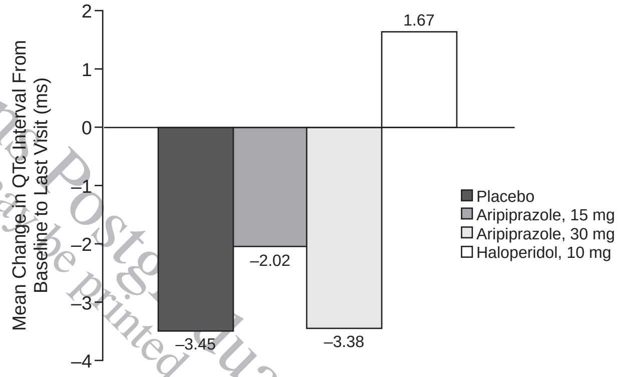

[a]Abbreviation: LOCF = last observation carried forward.

© Copyright 2002 Physicians Postgraduate Press, Inc.
One personal copy may be printed

groups (Figure 7). No patients receiving aripiprazole experienced a clinically significant increase in QTc interval (defined as a QTc ≥ 450 ms and a ≥ 10% increase from baseline). Three patients in the haloperidol group (3%) and 1 patient in the placebo group (1%) did experience clinically significant QTc interval increases. The highest recorded QTc intervals at any time point for each group were as follows: placebo: 454 ms; aripiprazole 15 mg: 453 ms; aripiprazole 30 mg: 453 ms; haloperidol: 471 ms.

Vital signs and laboratory analyses. There was no clinically significant difference in vital signs or laboratory results between treatment groups.

DISCUSSION

The current study found that aripiprazole 15 mg/day and 30 mg/day were effective, safe, and well tolerated for the treatment of patients with acute relapse of schizophrenia or schizoaffective disorder.

Aripiprazole 15 mg and 30 mg produced statistically significant improvements compared with placebo for change in PANSS total, PANSS positive, CGI-S, and PANSS-derived BPRS core scores. Both doses were also associated with significantly superior mean CGI-I scores compared with placebo. In addition, both aripiprazole 15 mg and 30 mg produced a significantly higher response rate than placebo. Aripiprazole, 15 mg, significantly improved PANSS negative subscale scores compared with placebo; the 30-mg dose of aripiprazole produced an improvement in negative symptoms, but this improvement did not differ significantly from that observed with placebo. For both doses of aripiprazole, mean change in PANSS total score from baseline separated from placebo by week 2. Analysis of PANSS total score by study center detected no significant treatment-by-center interaction.

The improvements in symptoms seen with aripiprazole treatment were comparable to those produced by haloperidol, which served as an active control in this study. Haloperidol improved all efficacy variables other than responder rate significantly more than placebo, and, like aripiprazole, separated from placebo on PANSS total score by week 2.

These efficacy data clearly indicate that aripiprazole 15 mg and 30 mg are effective in treating the acute symptoms of schizophrenia. These may also be the first data to demonstrate that a dopamine D_2 partial agonist can exhibit clinically meaningful and sustained improvements in schizophrenic symptoms, with efficacy sustained throughout the 4-week duration of the study. The mechanisms underlying the differences in the sustainability of efficacy of aripiprazole and the partial agonist –3-PPP are unknown. However, one can speculate that these differences may arise from the preclinical observation that the intrinsic activity of aripiprazole at D_2 receptors is less than that of –3-PPP.[14]

Treatment with aripiprazole was well tolerated at both treatment doses, and rates of adverse events did not vary consistently with dose. Adverse events were generally mild-to-moderate across all treatment groups and tended not to be treatment limiting. The rate of discontinuation due to adverse events was similar for all 3 active treatment groups, but was higher in the placebo group (16%). The adverse event most frequently cited as a reason for discontinuation was worsening of psychotic symptoms, which occurred most frequently in the placebo group.

Recently, significant attention has been focused on weight gain as a side effect of certain antipsychotic drugs[33]; weight gain due to antipsychotics has important implications for health with long-term use. In this study, all treatment arms produced very modest mean increases in body weight over the 4-week study period (range, 0.2–0.9 kg) that did not differ significantly from mean weight gain in the placebo group (Figure 5A). In the haloperidol 10-mg group, 10% of patients experienced clinically significant weight gain (≥ 7% increase from baseline), compared with 7% of patients in the aripiprazole 15-mg group, 4% of patients in the aripiprazole 30-mg group, and 1% in the placebo group (Figure 5B). A longer evaluation period would be needed to judge aripiprazole's weight gain potential more fully.

Prolonged QTc interval, an ECG abnormality that has been associated with certain antipsychotic drugs, produces a small but increased risk of potentially dangerous cardiac arrythmias.[34] In the current study, mean changes in QTc interval were not statistically different between any of the active treatment groups and placebo. No patient receiving aripiprazole experienced a clinically meaningful change in QTc interval, indicating that aripiprazole does not carry the risk of potentially fatal ECG changes.

Many currently available antipsychotic drugs are known to cause sedation, an undesirable side effect that can negatively impact a patient's functioning and adherence with prescribed therapy, especially in long-term treatment. The incidence of somnolence in the aripiprazole 15-mg group was comparable to placebo (5% and 4%, respectively), and the incidence of somnolence in the aripiprazole 30-mg group (10%) was not markedly elevated. The incidence of somnolence with haloperidol was 13%.

Extrapyramidal side effects and hyperprolactinemia have long been associated with antipsychotic drugs and have the potential to limit antipsychotic tolerability and effectiveness. The overall profile of the 2 aripiprazole doses for parkinsonism, akathisia, and dyskinesia was comparable to placebo. The 2 doses of aripiprazole were also comparable to placebo with regard to the incidence of adverse events related to EPS and requirements for concomitant medication. In contrast, haloperidol 10 mg was associated with the highest incidence of EPS-related adverse events and EPS-related adverse events requiring concomitant medication of any of the treatment groups.

Hyperprolactinemia can produce unwanted symptoms including sexual dysfunction, gynecomastia, amenorrhea, and galactorrhea.[35] Mean serum prolactin levels were not increased in either aripiprazole group from baseline to last visit, but increased markedly in the haloperidol group. This lack of increased serum prolactin with aripiprazole administration is consistent with findings from previous aripiprazole studies.[36,37] The low risk for both EPS and hyperprolactinemia with aripiprazole use observed in this and other aripiprazole studies may be explained by aripiprazole's partial agonism at D_2 receptors, in contrast to the D_2 antagonism of currently available antipsychotics.

The results of the present study indicate that aripiprazole has considerable potential for the treatment of psychotic disorders. The findings of the current study are consistent with the clinical effects predicted by aripiprazole's unique pharmacodynamic profile, which includes potent D_2 partial agonist activity combined with partial agonism at 5-HT_{1A} receptors and antagonism at 5-HT_{2A} receptors. These data also support the conclusion that aripiprazole is the first agent that is not a D_2 antagonist to demonstrate a rapid onset of action with sustained antipsychotic efficacy over 4 weeks. The antipsychotic effects of aripiprazole 15 mg and 30 mg given once daily were achieved with an excellent safety and tolerability profile, with no evidence of serum prolactin elevations or a marked potential for EPS, weight gain, or QTc prolongation. These data suggest that aripiprazole provides atypical antipsychotic efficacy with minimal side effect liabilities and the potential to lead to increased treatment adherence and decreased relapse rates.

© Copyright 2002 Physicians Postgraduate Press, Inc.
One personal copy may be printed

Drug names: benztropine (Cogentin and others), buspirone (BuSpar and others), clozapine (Clozaril and others), haloperidol (Haldol and others), lorazepam (Ativan and others), olanzapine (Zyprexa), reserpine (Serpalan and others), risperidone (Risperdal).

REFERENCES

1. Murray CJ, Lopez AD. Evidence-based health policy: lessons from the Global Burden of Disease Study. Science 1996;274:740–743
2. Bunney WE, Meltzer HY. Schizophrenia: overview. Clin Neurosci 1995;3:55–56
3. Seeman P, Niznik HB. Dopamine receptors and transporters in Parkinson's disease and schizophrenia. FASEB J 1990;4:2737–2744
4. Anden N, Butcher SG, Corrodi H, et al. Receptor activity and turnover of dopamine and noradrenaline after neuroleptics. Eur J Pharmacol 1970;11:303–314
5. Creese I, Burt DR, Snyder SH. Dopamine receptor binding predicts clinical and pharmacological potencies of antischizophrenic drugs. Science 1976;192:481–483
6. Carlsson A. The current status of the dopamine hypothesis of schizophrenia. Neuropsychopharmacology 1988;1:179–186
7. Emilien G, Maloteaux JM, Geurts M, et al. Dopamine receptors: physiological understanding to therapeutic intervention potential. Pharmacol Ther 1999;84:133–156
8. Richelson E. Receptor pharmacology of neuroleptics: relation to clinical effects. J Clin Psychiatry 1999;60(suppl 10):5–14
9. Fleischhacker WW, Meise U, Gunther V, et al. Compliance with antipsychotic drug treatment: influence of side effects. Acta Psychiatr Scand Suppl 1994;382:11–15
10. Kurzthaler I, Fleishhacker WW. The clinical implications of weight gain in schizophrenia. J Clin Psychiatry 2001;62(suppl 7):32–37
11. Lahti AC, Weiler MA, Corey PK, et al. Antipsychotic properties of the partial dopamine agonist (–)-3-(3-hydroxyphenyl)-N-n-propylpiperidine (preclamol) in schizophrenia. Biol Psychiatry 1998;43:2–11
12. Sramek JJ, Eldon MA, Posvar EL, et al. Initial safety, tolerability, pharmacodynamics and pharmacokinetics of CI–1007 in patients with schizophrenia. Psychopharmacol Bull 1998;34:93–99
13. Iyer RN, Davis MD, Juneau PL, et al. Brain extracellular levels of the putative antipsychotic CI–1007 and its effects on striatal and nucleus accumbens dopamine overflow in the awake rat. J Pharm Pharmacol 1998;50:1147–1153
14. Burris KD, Molski TF, Xu C, et al. Aripiprazole, a novel antipsychotic, is a high-affinity partial agonist at human dopamine D_2 receptors. J Pharmacol Exp Ther 2002;302:381–389
15. Jordan S, Koprivica V, Chen R, et al. The antipsychotic aripiprazole is a potent, partial agonist at the human 5-HT_{1A} receptor. Eur J Pharmacol 2002;441:137–140
16. McQuade RD, Burris KD, Jordan S, et al. Aripiprazole: a dopamine-serotonin system stabilizer. Int J Neuropsychopharmacol 2002(June); 5(suppl 1):S176
17. Carlsson A, Waters N, Waters S, et al. Network interactions in schizophrenia: therapeutic implications. Brain Res Rev 2000;31:342–349
18. Stahl SM. Dopamine system stabilizers, aripiprazole, and the next generation of antipsychotics, pt 1: "Goldilocks" actions at dopamine receptors [Brainstorms]. J Clin Psychiatry 2001;62:841–842
19. Stahl SM. Dopamine system stabilizers, aripiprazole, and the next generation of antipsychotics, pt 2: illustrating their mechanism of action [Brainstorms]. J Clin Psychiatry 2001;62:923–924
20. Kikuchi T, Tottori K, Uwahodo Y, et al. 7-(4-[(2,3-Dichlorophenyl)-1-piperazinyl]butyloxy)-3,4-dihydro-2(1H)-quinolinone (OPC-14597), a new putative antipsychotic drug with both presynaptic dopamine autoreceptor agonistic activity and postsynaptic D_2 receptor antagonist activity. J Pharmacol Exp Ther 1995;274:329–336
21. Inoue T, Domae M, Yamada K, et al. Effects of the novel antipsychotic agent 7-(4-[4-(2,3-dichlorophenyl)-1-piperazinyl]butyloxy)-3,4-dihydro-2(1H)-quinolinone (OPC-14597) on prolactin release from the rat anterior pituitary gland. J Pharmacol Exp Ther 1996;277:137–143
22. Glennon RA, Dukat M. Serotonin receptor subtypes. In: Bloom FE, Kupfer DJ, eds. Psychopharmacology: The Fourth Generation of Progress. New York, NY: Raven Press; 1995:419
23. Jann MW. Buspirone: an update on a unique anxiolytic agent. Pharmacotherapy 1988;8:100–116
24. Sovner R, Parnell-Sovner N. Use of buspirone in the treatment of schizophrenia. J Clin Psychopharmacol 1989;9:61–62
25. Millan MJ. Improving the treatment of schizophrenia: focus on serotonin (5-HT)1A receptors. J Pharmacol Exp Ther 2000;295:853–861
26. Leysen JE, Janssen PMF, Schotte A, et al. Interaction of antipsychotic drugs with neurotransmitter receptor sites in vitro and in vivo in relation to pharmacological and clinical role of 5-HT_2 receptors. Psychopharmacology 1993;112:S40
27. Rao ML, Muller HJ. Biochemical findings of negative symptoms in schizophrenia and their positive relevance to pharmacologic treatment. Neuropsychobiology 1994;30:160–164
28. Kay SR, Fiszbein A, Opler LA. The Positive and Negative Syndrome Scale (PANSS) for schizophrenia. Schizophr Bull 1987;13:261–276
29. Clinical Global Impressions (CGI). In: Guy W. ECDEU Assessment Manual for Psychopharmacology. US Dept Health, Education, and Welfare publication (ADM) 76-338. Rockville, Md: National Institute of Mental Health; 1976:218–222
30. Simpson GM, Angus JWS. A rating scale for extrapyramidal side effects. Acta Psychiatr Scand Suppl 1970;45(suppl 212):11–19
31. Barnes TRE. A rating scale for drug-induced akathisia. Br J Psychiatry 1989;154:672–676
32. Abnormal Involuntary Movement Scale (AIMS). In: Guy W. ECDEU Assessment Manual for Psychopharmacology. US Dept Health, Education, and Welfare publication (ADM) 76-338. Rockville, Md: National Institute of Mental Health; 1976:534–537
33. Allison DB, Casey DE. Antipsychotic-induced weight gain: a review of the literature. J Clin Psychiatry 2001;62(suppl 7):22–31
34. Gury C, Canceil O, Iaria P. Antipsychotic drugs and cardiovascular safety: current studies of prolonged QT interval and risk of ventricular arrhythmia. Encephale 2000;26:62–72
35. Dickson RA, Glazer WM. Neuroleptic-induced hyperprolactinemia. Schizophr Res 1999;35:S75–S86
36. Carson WH, McQuade RD, Ali M, et al. A double-blind, placebo-controlled trial of aripiprazole and haloperidol. Schizophr Res 2001;49 (suppl 1):221
37. Daniel DG, Saha AR, Ingenito G, et al. Aripiprazole, a novel antipsychotic: overview of a phase II study result. Int J Neuropsychopharmacol 2000;3(suppl 1):S157

© Copyright 2002 Physicians Postgraduate Press, Inc.
One personal copy may be printed

Acknowledgment

Investigator Study Group: Frank Abuzzahab, M.D. Ph.D.; Bernardo J. Arias, M.D.; Bijan Bastani, M.D.; Louise Beckett, M.D.; Robert Bielski, M.D.; J. Gary Booker, M.D.; David Brown, M.D.; John Carman, M.D.; Ronald Centric, D.O.; Stanley Cheren, M.D.; Edward A. Cherlin, M.D.; Cal Cohn, M.D.; David Richard Cox; David Daniel, M.D.; Larry Ereshefsky, Pharm.D.; Louis F. Fabre, M.D., Ph.D.; Jay Feierman, M.D., P.C.; Craig Fischer, M.D.; Arnold Friedhoff, M.D.; James Furguson, M.D.; Steven Glass, M.D.; Hal Goldberg, M.D.; Clifford Goldman, M.D.; Alan I. Green, M.D.; Barry H. Guze, M.D.; Mahlon Hale, M.D.; Mark B. Hamner, M.D.; Harold Harsch, M.D.; James Hartford, M.D.; Alan Jacobson, Ph.D.; Mary Ann Knesevich, M.D.; Irving Kolin, M.D.; Joseph Kwentus, M.D.; William Lawson, M.D.; Mark N. Lerman, M.D.; Robert Levine, M.D.; Robert Litman, M.D.; Michael McLarnon, M.D.; Charles Meredith, M.D.; Alexander L. Miller, M.D.; B. Rhett Myers, M.D.; Jorg J. Pahl, M.D., F.C.P. (S.A.); Richard Pearlman, M.D.; Steven G. Potkin, M.D.; Duttala Reddy, M.D.; Neil Richtand, M.D., Ph.D.; Robert A. Riesenberg, M.D.; Samuel Risch, M.D.; J. J. Rodos, D.O.; Murray H. Rosenthal, D.O.; Anthony Rothschild, M.D.; David A. Sack, M.D.; Kenji Sax, Ph.D.; Joyce Small, M.D.; Thomas E. Smith, M.D.; Kenneth Sokolski, M.D.; Andrea Stone, M.D.; Stephen Strakowski, M.D.; Tram Tran-Johnson, Pharm.D., Psy.D.; Scott A. West, M.D.; Craig Wronski, D.O.; John Zajecka, M.D.; Dan Zimbroff, M.D.
