# Supplementary material for: Dose-Dependent Efficacy of Aripiprazole in Treating Patients With Schizophrenia or Schizoaffective Disorder: A Systematic Review and Meta-Analysis of Randomized Controlled Trials
Source: Front Psychiatry. 2021 Aug 11;12:717715. doi: 10.3389/fpsyt.2021.717715 (PMC8385236; doi:10.3389/fpsyt.2021.717715)
Supplement: Supplementary file 1 [file Data_Sheet_1.ZIP › supplementray material-9 RCTs/9 Zhu 2019.pdf]

·药物与临床·

不同剂量阿立哌唑治疗精神分裂症的效果对比分析

朱丽荣

（忻州市荣军精神病医院，山西 忻州 034000）

摘 要：目的 研究对比精神分裂症患者实施不同剂量阿立哌唑治疗的临床效果和安全性。方法 纳入 2016 年 10 月至 2018 年 10 月，本院收治的精神分裂症患者 100 例作为研究对象，按随机表法分为实验组（n=50）、对照组（n=50），实验组：阿立哌唑 30 mg/ 天治疗，对照组：阿立哌唑 20 mg/ 天治疗，观察两组病情严重程度、不良反应发生率、生活质量、阴性、阳性症状评分、泌乳素水平。结果 病情严重程度、不良反应发生率对比：实验组低于对照组；生活质量对比：实验组高于对照组；阴性、阳性症状评分：实验组低于对照组；泌乳素水平对比：实验组低于对照组，$P<0.05$。结论 精神分裂症患者实施 30 mg/ 天大剂量阿立哌唑治疗临床效果显著，既可降低不良反应发生率，还可改善生活质量，值得借鉴。

关键词：不同剂量；阿立哌唑；慢性精神分裂症；临床效果

中图分类号：R749.3 文献标识码：B DOI：10.19613/j.cnki.1671-3141.2019.55.088

本文引用格式：朱丽荣.不同剂量阿立哌唑治疗精神分裂症的效果对比分析[J].世界最新医学信息文摘,2019,19(55):137-138.

0 引言

精神分裂症是以感知、思维、情感、意志、行为、认知等障碍为特征的迁延性疾病，好发于青壮年，且病程呈现反复发作、加重特性，该疾病患者社会功能多严重受损，故临床治疗效果较差。临床对该疾病的研究、探索从未停止，但现阶段对精神分裂症发病的具体病因尚无明确定论，多认为疾病的发生与遗传因素、患者个性特征、社会环境不良因素、神经生化异常及脑结构异常改变等有一定联系，精神分裂症以幻觉、妄想为主要特征，并在幻觉、妄想影响下出现情感及意志行为等方面的异常，最常见症状为妄想，如：被害妄想、关系妄想、嫉妒妄想等，且据资料显示[1]，8/10 精神分裂症患者伴有被害妄想。临床多实施药物治疗联合心理治疗。常见阿立哌唑药物，其被临床认为是多巴胺系统的平衡剂，但在使用过程中若未进行剂量调节，便会增加高催乳素血症等不良反应发生率，降低疗效，故本次随机选择 100 例精神分裂症患者，为其实施不同剂量阿立哌唑进行治疗，观察其临床疗效及安全性，总结如下。

1 资料与方法

1.1 一般资料。纳入我院（2016 年 10 月至 2018 年 10 月）收治的 100 例精神分裂症患者，分为 2 组（随机表法），即每组 50 例样本。实验组中男 20 例，女 30 例；年龄在 18-40 岁，平均（28.5±9.5）岁；病程在 6-20 个月，平均（13.0±7.0）个月；首发患者 33 例，复发患者 17 例；奥氮平治疗患者 20 例，喹硫平治疗患者 10 例，氯丙嗪治疗患者 11 例，利培酮治疗患者 9 例。对照组中男 19 例，女 31 例；年龄在 17-41 岁，平均（29.0±12.0）岁；病程在 5-15 个月，平均（10.0±5.0）个月；首发患者 34 例，复发患者 16 例；奥氮平治疗患者 21 例，喹硫平治疗患者 11 例，氯丙嗪治疗患者 10 例，利培酮治疗患者 8 例；精密、细致对比 2 组基础资料，符合临床研究相关规定 P>0.05，待患者及家属了解相关治疗方法、研究目的，自愿签署知情同意书后，上报医院伦理委员会获得批准可实施研究。研究纳入：①结合《精神分裂症诊疗指南》确诊患者；②年龄在 16 岁以上患者；③研究前使用精神类药物治疗患者；④高催乳素血症患者，即：女性 ≥12.8 mmol/l，男性 ≥7.8 mmol/l；排除：①药物过敏患者；②恶性肿瘤患者；③严重心脑血管疾病患者；④多卵巢囊肿患者；⑤语言障碍患者；退出：①患者或家属要求退出患者；②病情加重或减退不愿继续治疗患者；③严重不良反应患者，避免对整体研究效果产生影响。

1.2 方法。纳入研究 100 例精神分裂症患者均实施阿立哌唑（国药准字 H20140121，生产企业：江苏恩华药业股份有限公司，规格：10 mg/ 片）口服治疗，实验组患者 30 mg/ 次，1 次 / 天；对照组患者 20 mg/ 次，1 次 / 天，治疗 4 月。

1.3 分析指标

1.3.1 病情严重程度评估：采用 CGI-SI 量表评估，分值 0-10 分，得分与病情严重程度呈反比[2]。

1.3.2 不良反应发生率评估：临床常见不良反应有失眠、困倦、焦虑、头晕、便秘、食欲下降等。

1.3.3 生活质量评估：采用 SF-36 量表进行评估，分值 0-100 分，得分与患者生活质量改善程度呈正比。

1.3.4 阴性、阳性症状评分：采用 PANSS 量表有经验丰富、年资高精神科医师独立进行评估。

1.3.5 泌乳素水平评估：于空腹状态下采集静脉血 5 mL，标本采集完毕放置在抗凝试管内采用全自动化学仪测定[3]。

1.4 统计学分析。SPSS 22.0，计量资料（泌乳素水平、病情严重程度、生活质量、阴性、阳性症状评分）用 t、（$\bar{x}\pm s$）进行检验、表示，计数资料（不良反应发生率）以 χ^2、% 进行检验、表示，2 组数据资料，以 P 值区间进行表示。

2 结果

2.1 病情严重程度。两组病情严重程度评估，详见表 1。数据显示：治疗前无差异，实验组治疗后 1 个月、2 个月、3 个月评分均显著下降。

表 1 病情严重程度评估（$\bar{x}\pm s$）

组别	例数	治疗前	治疗后 1 个人月	治疗后 2 个月	治疗后 3 个月
对照组	50	6.02±1.02	5.72±0.95	4.25±1.82	2.75±1.03
实验组	50	6.05±0.98	5.08±1.05	3.96±1.21	2.42±0.95
t	-	0.1500	3.1960	0.9383	1.6653
P	-	0.8811	0.0019	0.3504	0.9900

2.2 不良反应发生率。两组不良反应发生率评估，详见表 2。数据显示：实验组低于对照组。

2.3 阴性、阳性症状评分。两组阴性、阳性症状评分评估，详见表 3。数据显示：治疗前无差异，实验组治疗后 1 个月、2 个月、3 个月评分均显著下降。

表 2 不良反应发生率评估 [n（%）]

组别	例数	失眠	困倦	焦虑	头晕	便秘	食欲下降
对照组	50	2（4.00）	1（2.00）	3（6.00）	2（4.00）	3（6.00）	4（8.00）
实验组	50	8(16.00)	7(14.00)	10(20.00)	9(18.00)	11(22.00)	12（24.00）
χ^2	-	4.0000	4.8913	4.3324	5.0051	4.3324	4.7619
P	-	0.0455	0.0270	0.0374	0.0253	0.0374	0.0291

表 3 阴性、阳性症状评分评估（$\bar{x}\pm s$）

组别	例数	治疗前	治疗后 1 个月	治疗后 2 个月	治疗后 3 个月
对照组	50	89.65±10.81	87.72±6.54	81.82±5.43	70.91±4.63
实验组	50	90.02±9.58	74.23±5.41	73.25±7.62	61.52±10.32
t	-	0.1810	11.2386	6.4765	5.8701
P	-	0.8567	0.0000	0.0000	0.0000

2.4 泌乳素水平。两组泌乳素水平评估，详见表4。数据显示：治疗前无差异，实验组治疗后 1 个月、2 个月、3 个月泌乳素水平均显著下降。

表 4 泌乳素水平评估（$\bar{x}\pm s$）

组别	例数	治疗前	治疗后 1 个月	治疗后 2 个月	治疗后 3 个月
对照组	50	41.92±12.61	43.45±12.31	42.35±10.52	41.42±10.21
实验组	50	42.11±12.52	30.22±12.52	27.61±12.51	13.11±10.52
t	-	0.0756	5.3280	6.3766	13.6550
P	-	0.9399	0.0000	0.0000	0.0000

2.5 生活质量。两组生活质量评估，详见表 5。数据显示：治疗前无差异，实验组治疗后 1 个月、2 个月、3 个月生活质量均明显提升。

表 5 生活质量评估（$\bar{x}\pm s$）

组别	例数	治疗前	治疗后 1 个月	治疗后 2 个月	治疗后 3 个月
对照组	50	33.56±15.21	38.69±16.36	48.82±14.25	59.22±10.15
实验组	50	32.53±15.96	53.31±12.11	67.35±10.22	73.12±10.08
t	-	0.3304	5.0790	7.4719	6.8709
P	-	0.7418	0.0000	0.0000	0.0000

3 讨论

精神分裂症患者治愈率较低，且易复发，故在临床上多需长期服用药物进行治疗，目前临床常规使用第二代抗精神病药物，这些药物在受体结合方面具有较高的 5-HT2 受体阻断作用，称 DA-5-HT 受体拮抗剂，对中脑边缘系统的作用比对纹状体系统的作用更具有选择性。但此类药物在达到治疗目的的同时会引起体内泌乳素水平明显升高，影响患者生活质量。有资料显示，阿立哌唑应用于精神分裂症患者治疗中，对缓解多巴胺受体过度拮抗出现的泌乳素水平有极高价值，可明显降低高泌乳素血症的发生率。

研究数据中，实验组治疗后 1 个月、2 个月、3 个月泌乳素水平为（30.22±12.52）mmol/L、（27.61±12.51）mmol/L、(13.11±10.52)mmol/L较对照组明显下降，阴性、阳性症状评分均低于对照组，且不良反应发生率低于对照组，这与李猛 [4] 等研究一致。分析：结合数据资料，阿立哌唑使用后不仅具有极高安全性，且对泌乳素水平无明显影响，对于临床闭经状态下女性患者可选择使用，不会对其生活质量产生严重影响。阿立哌唑属于新型抗精神类药物，且与大脑多巴胺受体激动性有一定联系，药物进入人体后，在体内多巴胺活性较高情况下，可有效阻断多巴胺受体，在体内多巴胺活性较低情况下，可表现出多巴胺受体激动性，达到调节体内多巴胺水平，平衡泌乳素水平的目的。数据资料对比显示，30 mg/ 天阿立哌唑的摄入对病情改善程度较 20 mg/ 天阿立哌唑明显，结合药物合理使用数据分析，阿立哌唑使用剂量越高，在对多巴胺组分实施阻断过程中作用越明显，故该剂量使用后患者生活质量明显改善，且泌乳素水平明显下降。但在患者具体使用中，对阿立哌唑剂量的调节需以合理药物数据分析为前提，并结合患者实际病情和药物耐受程度，避免增加不良反应发生率，影响整体治疗效果 [5]。

综合上述，精神分裂症患者实施大剂量阿立哌唑（30 mg/ 天）治疗临床效果显著，既可降低不良反应发生率，还可改善生活质量，值得借鉴，且 30 mg/ 天阿立哌唑治疗后泌乳素水平明显降低，症状评分、病情严重程度均得到明显改善，故该方法临床价值得到证实。但基于本次研究时间段较短，且选取样本数量较少，未对患者心理状态进行评估，故在后期研究中需加大研究样本，延长研究时间，对患者心理状态恢复情况进行分析、数据统计，为临床应用提供更高参考价值。

参考文献

[1] 程国强，钟健荣，鲁金韵，等．不同剂量阿立哌唑治疗慢性精神分裂症的效果对比 [J]. 慢性病学杂志 ,2018,19(07):972-973.
[2] 胡怡，王琪珠，黄朝红，等．不同剂量阿立哌唑对首发精神分裂症女性患者服用抗精神病药物致高泌乳素血症的治疗 [J]. 昆明医科大学学报 ,2017,38(12):89-92.
[3] 卓东炳，王广积，王达君，等．不同剂量阿立哌唑对利培酮治疗精神分裂症患者引起的高催乳素血症的影响 [J]. 国际精神病学杂志 ,2017,44(01):56-59.
[4] 李猛，张淑芳，邵玉单，等．不同剂量阿立哌唑对氨磺必利致女性精神分裂症患者催乳素升高的影响 [J]. 中国健康心理学杂志 ,2016,24(02):175-178.
[5] 彭瑾，刘君．奥氮平、利培酮及阿立哌唑治疗慢性精神分裂症患者认知功能损害的疗效观察 [J]. 淮海医药 ,2015,33(05):439-440.

（上接第 136 页）

参考文献

[1] 官威，鞠东升，姚静，等．尼莫地平与阿司匹林治疗偏头痛效果和安全性的临床研究 [J]. 中国医药指南 ,2018,16(20):99-100.
[2] 袁冬莉，谭忠宜，李王文，等．阿米替林治疗伴有抑郁的偏头痛患者的疗效评估 [J]. 世界复合医学 ,2017,3(04):74-76.
[3] 曾利．阿司匹林与尼莫地平治疗偏头痛的效果比较研究 [J]. 中国社区医师 ,2017,33(20):58-59.
[4] 梁为民，谢章科．阿米替林与黛力新防治偏头痛发作的疗效比较 [J]. 泰山医学院学报 ,2016,37(02):168-169.
[5] 李可．奥卡西平与阿米替林联合治疗偏头痛临床疗效观察 [J]. 北方药学 ,2017,14(06):120-121.
[6] 张志华．偏头痛治疗中阿司匹林与尼莫地平效果的对比性评价研究 [J]. 赤峰学院学报（自然版）,2016(9):36-37.
[7] 白金娟，张红利．对比分析盐酸氟桂利嗪与尼莫地平治疗偏头痛的临床效果 [J]. 中外医疗 ,2015(7):101-102.
[8] 张士良．阿司匹林与尼莫地平在偏头痛治疗中的效果比较分析 [J]. 临床医药文献电子杂志 ,2014,1(8):1353-1353.
