# Supplementary material for: Dose-Dependent Efficacy of Aripiprazole in Treating Patients With Schizophrenia or Schizoaffective Disorder: A Systematic Review and Meta-Analysis of Randomized Controlled Trials
Source: Front Psychiatry. 2021 Aug 11;12:717715. doi: 10.3389/fpsyt.2021.717715 (PMC8385236; doi:10.3389/fpsyt.2021.717715)
Supplement: Supplementary file 1 [file Data_Sheet_1.ZIP › supplementray material-9 RCTs/4 Findling 2008.pdf]

A Multiple-Center, Randomized, Double-Blind, Placebo-Controlled Study of Oral Aripiprazole for Treatment of Adolescents With Schizophrenia

Robert L. Findling, M.D.

Adelaide Robb, M.D.

Margaretta Nyilas, M.D.

Robert A. Forbes, Ph.D.

Na Jin, M.S.

Svetlana Ivanova, Ph.D.

Ronald Marcus, M.D.

Robert D. McQuade, Ph.D.

Taro Iwamoto, Ph.D.

William H. Carson, M.D.

Objective: Aripiprazole is a dopamine partial agonist approved for use in adults for short- and long-term treatment of schizophrenia and bipolar disorder. This study was designed to examine the acute efficacy, safety, and tolerability of aripiprazole for adolescents with schizophrenia.

Method: This was a 6-week multicenter, double-blind, randomized, placebo-controlled trial. Subjects 13 to 17 years old with a DSM-IV diagnosis of schizophrenia and a Positive and Negative Syndrome Scale (PANSS) total score of 70 or more were randomly assigned (1:1:1 ratio) to placebo or 10 or 30 mg/day of aripiprazole. The primary endpoint was mean change from baseline to endpoint (last observation carried forward) in PANSS total score. Assessments of safety and tolerability included spontaneously reported adverse events, extrapyramidal symptom scores, serum prolactin concentration, body weight, and metabolic measures.

Results: Of 302 patients, 85% completed the 6-week study. The mean baseline PANSS score was 94.1. At the end of the study, both aripiprazole doses showed statistically significant differences from placebo in reduction in PANSS total score. Adverse events occurring in more than 5% of either aripiprazole group and with a combined incidence at least twice the rate for placebo were extrapyramidal disorder, somnolence, and tremor. Mean changes in prolactin were –8.45, –11.93, and –15.14 ng/ml for placebo and 10 mg and 30 mg of aripirazole, respectively. Mean body weight changes were –0.8, 0.0, and 0.2 kg for placebo and 10 mg and 30 mg of aripiprazole, respectively.

Conclusion: Both 10- and 30-mg/day doses of aripiprazole were superior to placebo in the acute treatment of adolescents with schizophrenia. Aripiprazole was generally well tolerated.

(Am J Psychiatry 2008; 165:1432–1441)

Although childhood-onset schizophrenia is rare, adolescent-onset schizophrenia is relatively common in males, with up to 39% experiencing their first episode before age 20 (1–4). Adolescents with schizophrenia have impairments in psychosocial functioning (5) and have generally poor outcomes (6–9).

Safe and effective treatments are needed for youths, but there is a paucity of double-blind and placebo-controlled treatment studies in adolescents with schizophrenia. In a 4-week study of 75 adolescents with schizophrenia between the ages of 13 and 18 years, treatment with either loxapine or haloperidol was reported to be superior to placebo in reducing symptoms of psychosis (10). The most common side effects reported in that trial were extrapyramidal symptoms and sedation. In an 8-week study in which 107 adolescents between the ages of 13 and 17 years were randomly assigned in a 2:1 ratio to receive either olanzapine or placebo in a double-blind fashion, active treatment with olanzapine was associated with greater reductions in psychiatric symptoms than placebo (11). In that study, weight gain and sedation were reported to occur more commonly with active treatment than with placebo. The study also found elevations in cholesterol, triglycerides, glucose, insulin, and prolactin with active treatment when compared with placebo.

Aripiprazole is a dopamine partial agonist that has been shown to be efficacious in the treatment of positive and negative symptoms in acute and long-term treatment of schizophrenia or schizoaffective disorder in adults at doses of 10 to 30 mg/day (12–15). Because youths may be more vulnerable to antipsychotic-induced extrapyramidal symptoms (16) and weight gain (17), a medication's side effect profile is particularly relevant when treating adolescents.

This randomized, double-blind, placebo-controlled clinical trial assessed the efficacy and tolerability of aripiprazole in the acute treatment of adolescents with schizophrenia. The a priori hypotheses were that aripiprazole would be superior to placebo in ameliorating symptoms of psychosis and would be generally well tolerated.

This article is featured in this month's AJP Audio and is discussed in an editorial by Dr. Ross (p. 1369).

Method

Study Design

In accordance with the U.S. Code of Federal Regulations (CFR Title 21, Part 50), written, informed consent was obtained from all subjects' guardians or legal representatives, and written assent was obtained from each adolescent subject before any study-related procedures were performed. The protocol, procedures, consent, and assent statements (per guidelines of the U.S. Food and Drug Administration [FDA] and the International Conference on Harmonisation of Technical Requirements for Registration of Pharmaceuticals for Human Use) were approved by the institutional review board or ethics committee of each participating center (18, 19).

This was a phase III, randomized, placebo-controlled study. The trial was conducted in 101 centers in the United States, Europe, South America, Asia, the Caribbean, and South Africa over a 23-month enrollment period. Randomization was stratified and equally maintained across each of three geographical categories: 1) United States, 2) Europe, and 3) other regions. To assure reliability of ratings among sites, the number of raters within each site was minimized and efforts were to be made to ensure that the same rater administered the Positive and Negative Syndrome Scale (PANSS) (20, 21) for a given patient. Raters at each site had identical instructions for administering the PANSS, and each site was required to demonstrate interrater reliability on an ongoing basis throughout the period in which patients participated in the study.

Patients

Subjects who were deemed appropriate candidates by their treating physicians were screened for eligibility within 4 weeks of baseline. Eligible subjects met the following inclusion criteria: 1) male or female, 2) age 13 to 17 years inclusive, 3) DSM-IV (22) axis I primary diagnosis of schizophrenia and confirmation of the schizophrenia diagnosis by an adequately trained clinician (e.g., child psychiatrist) at the time of screening, by means of the Schedule for Affective Disorders and Schizophrenia for School-Age Children—Present and Lifetime Version (K-SADS-PL) (23), and 4) a baseline PANSS score of 70 or higher. Eligible subjects were randomized in a 1:1:1 ratio to receive placebo, 10 mg/day of aripiprazole, or 30 mg/day of aripiprazole for 6 weeks. On the basis of an effective and well-tolerated dosing schedule used in previous sponsor-conducted trials (24–26) and the FDA written request to examine the dose range of aripiprazole for adults (12–15), eligible subjects received aripiprazole according to a forced-titration scheme to achieve target doses of either 10 or 30 mg/day. In the 10-mg group, aripiprazole was titrated from a starting dose of 2 mg/day to 5 mg/day on day 3, followed by an increase to the target dose of 10 mg/day on day 5. Subjects in the 30-mg group also began at 2 mg/day; the dose was then increased every 2 days to 5 mg/day, 10 mg/day, 15 mg/day, 20 mg/day, and finally, the target dose of 30 mg/day on day 11. Target doses were maintained for at least 2 weeks. Subjects who experienced unacceptable dose-related tolerability problems before study day 25 were removed from the study. After day 25, a dose reduction to a minimum of either 5 or 15 mg/day was permitted for subjects in the 10-mg and 30-mg cohorts, respectively. After dose reduction, a return to the initial target dose was not permitted. Medication was administered once daily.

Subjects were required to discontinue prohibited medications, including mood stabilizers, antidepressants, and other psychotropics, at least 3 days before the initiation of treatment. Fluoxetine must have been discontinued for 4 weeks, and stimulants and atomoxetine must have been discontinued for at least 5 half-lives before the first dose of study drug. During the washout period, benzodiazepines (lorazepam or equivalent) or anticholinergic agents were permitted as clinically indicated. During the course of the study, if the primary efficacy measure was unchanged or worsened, or if deemed absolutely necessary by the investigator, subjects were permitted to receive benzodiazepine or anticholinergic medications for relief of transient symptoms. Treatment with benzodiazepines within 4 hours or with anticholinergic agents within 12 hours before efficacy ratings or administration of movement scales was prohibited.

Subjects were excluded if they had any current psychiatric comorbidity requiring pharmacotherapy; any evidence of suicide risk; or a history or current diagnosis of schizoaffective disorder, mental retardation, major depressive episodes, neuroleptic malignant syndrome, any neurologic disorder other than Tourette's syndrome, severe head trauma, or any unstable medical condition. Subjects whose illness was resistant to antipsychotics according to prior trials of two different antipsychotics of adequate dose and duration were also excluded, as were adolescent girls who were pregnant or breast-feeding and sexually active adolescent boys or adolescent girls who did not agree to abstinence or birth control. Subjects with positive screens for illegal drugs within 3 months of baseline or during the study were either excluded or removed from the study, respectively. Additionally, subjects who had been hospitalized for acute schizophrenia within 2 weeks or enrolled in a clinical trial within 4 weeks of baseline testing were excluded. Subjects were permitted to participate in this study on an outpatient, partial hospitalization, or full inpatient basis at any given time of the study.

Efficacy Assessments

The a priori primary efficacy assessment was the mean change from baseline to the last observed postbaseline visit (with the last observation carried forward) in total score on the PANSS. Assessments were performed at baseline and at weekly visits through day 42, which was the end of the study (week 6).

The secondary efficacy measures included the mean change in the PANSS positive and negative subscale scores from baseline to the last observation. The secondary measures also included the Clinical Global Impression (CGI) improvement and severity scales (27) and the clinician-rated Children's Global Assessment Scale (28). The latter instrument was completed at baseline and at day 42 or the end of treatment.

Quality of life and patient satisfaction were assessed by using the Pediatric Quality of Life Enjoyment and Satisfaction Questionnaire (29). Both the "total score" and a separate "overall score" item were determined at screening and at day 42.

Safety and Tolerability Assessments

To avoid bias in responses about adverse events, the subjects (and their parents or guardians) were asked, "How have you felt since your last visit?" The investigators were required to record all spontaneously reported adverse events at all scheduled assessments.

Side effects related to extrapyramidal symptoms were assessed at each visit as spontaneously reported adverse events. Extrapyramidal symptoms were also monitored at each scheduled visit by means of the Simpson-Angus Scale (30), the Barnes Rating Scale for Drug-Induced Akathisia (31), and the Abnormal Involuntary Movement Scale (AIMS) (32).

Metabolic evaluations included body weight, body mass index, diastolic and systolic blood pressure, and fasting levels of serum triglycerides, high-density lipoprotein (HDL) cholesterol, and glucose. Blood samples for measuring triglycerides, HDL cholesterol, and glucose were obtained after a minimum 8-hour fast at baseline, day 28, and day 42 (or end of study).

Additional assessments included ECG, physical examinations, vital signs, and number of hospitalizations. An independent five-member data safety monitoring board was informed of adverse

TABLE 1. Characteristics of Adolescents With Schizophrenia in a Placebo-Controlled Trial of Aripiprazole

Group and Characteristic	Placebo			Aripiprazole, 10 mg/day			Aripiprazole, 30 mg/day		
	N	Data		N	Data		N	Data	
**Total study group**	100			100			102		
		**Mean**	**SD**		**Mean**	**SD**		**Mean**	**SD**
Age (years)		15.4	1.4		15.6	1.3		15.4	1.4
Height (cm)		166.0	10.0		164.0	10.8		167.1	11.4
Weight (kg)		63.35	15.63		63.53	19.11		64.54	15.49
Body mass index (kg/m^2)		22.9	5.3		23.5	6.2		23.0	4.9
Baseline scores									
Positive and Negative Syndrome Scale		94.6	15.6		93.6	15.7		94.0	16.1
CGI severity scale		4.6	0.8		4.5	0.8		4.6	0.6
Children's Depression Rating Scale, Revised (33)		1.3	0.5		1.3	0.6		1.3	0.6
Disease duration (years)		1.5	2.3		1.4	2.3		1.3	1.6
Age at onset (years)		14.0	2.6		14.2	2.5		14.2	2.0
		**N**	**%**		**N**	**%**		**N**	**%**
Onset before age 13		23	23		15	15		15	15
Onset at age 13–17		77	77		85	85		87	85
Received treatment for previous episode[a]		73	73		75	75		75	74
Used antipsychotic before study		46	46		53	53		47	46
Atypical antipsychotic		43	43		44	44		36	35
Typical antipsychotic		8	8		13	13		17	17
Race									
Caucasian		64	64		54	54		62	61
African American		6	6		17	17		11	11
Asian		15	15		16	16		12	12
American Indian or Alaska Native		1	1		0	0		0	0
Other		14	14		13	13		17	17
Hispanic/Latino ethnicity		14	14		12	12		15	15
**Male patients**	61			45			65		
		**Mean**	**SD**		**Mean**	**SD**		**Mean**	**SD**
Age (years)		15.3	1.4		15.6	1.3		15.5	1.3
Height (cm)		170.9	8.9		170.1	10.6		172.3	9.5
Weight (kg)		65.93	15.36		66.74	18.82		67.92	15.68
Body mass index (kg/m^2)		22.5	4.8		22.8	9.7		22.9	5.2
		**N**	**%**		**N**	**%**		**N**	**%**
Race									
Caucasian		42	69		27	60		42	65
African American		5	8		7	16		7	11
Asian		8	13		5	11		5	8
American Indian or Alaska Native		0	0		0	0		0	0
Other		6	10		6	13		11	17
Hispanic/Latino ethnicity		6	10		6	13		10	15
**Female patients**	39			55			37		
		**Mean**	**SD**		**Mean**	**SD**		**Mean**	**SD**
Age (years)		15.6	1.5		15.6	1.3		15.4	1.5
Height (cm)		158.5	6.3		158.9	8.1		158.1	8.5
Weight (kg)		59.32	15.38		60.90	19.11		58.60	14.94
Body mass index (kg/m^2)		23.6	5.9		24.0	6.0		23.2	4.4
		**N**	**%**		**N**	**%**		**N**	**%**
Race									
Caucasian		22	56		27	49		20	54
African American		1	3		10	18		4	11
Asian		7	18		11	20		7	19
American Indian or Alaska Native		1	3		0	0		0	0
Other		8	21		7	13		6	16
Hispanic/Latino ethnicity		8	21		6	11		5	14

[a] Twenty-six percent of the patients were drug naive.

events throughout the course of the study for the purpose of ensuring patient safety.

Statistical Analyses

Using a two-sided alpha at the 0.025 level of significance, we determined that approximately 255 subjects (85 subjects per treatment arm) were required to provide at least an 85% power to detect a between-group difference of –11.4 in the mean change from baseline in the primary efficacy assessment (PANSS total score). This study group size was based on the assumption that variability in this assessment would be similar to that in adult schizophrenia trials over the 2- to 10-mg/day and 2- to 30-mg/day ranges, which resulted in a –11.4 median of the mean differences with a pooled standard deviation of 22.5 (12–15). Enrollees without a baseline or a postbaseline PANSS value were not included in the primary analysis of change from baseline in PANSS total score.

Statistical analysis was performed by fitting an analysis of covariance (ANCOVA) model to the PANSS change scores with right-hand terms for treatment, regional strata as factors, and baseline total score as covariate. The least-squares means derived from

Total		
N	Data	
302		
	**Mean**	**SD**
	15.5	1.4
	165.7	10.8
	63.81	16.93
	23.1	5.4
	94.1	15.8
	4.6	0.8
	1.3	0.6
	1.4	2.1
	14.1	2.4
	**N**	**%**
	53	18
	249	82
	223	74
	146	48
	123	41
	38	13
	180	60
	34	11
	43	14
	1	0
	44	15
	41	14
171		
	**Mean**	**SD**
	15.4	1.3
	171.2	9.6
	66.90	16.38
	22.7	4.9
	**N**	**%**
	111	65
	19	11
	18	11
	0	0
	23	13
	22	13
131		
	**Mean**	**SD**
	15.5	1.4
	158.5	9.6
	59.78	16.84
	23.7	5.9
	**N**	**%**
	69	53
	15	11
	25	19
	1	1
	21	16
	19	15

this model were used for comparing placebo with each active treatment. A nominal overall significance level of 0.05 (two-tailed) was used in testing the statistical significance of these two comparisons. The Hochberg's procedure was used to account for multiplicity in testing the two comparisons, with the following criteria: if both p values were <0.05 (two-tailed), then statistical significance was declared for both doses; if the larger of the two p values was >0.05, then the smaller p value was compared with 0.025 (two-tailed). The corresponding treatment comparison was declared statistically significant if the p value was <0.025.

FIGURE 1. Change in Total Score on the Positive and Negative Syndrome Scale for Adolescents With Schizophrenia in a Placebo-Controlled Trial of Aripiprazole[a]

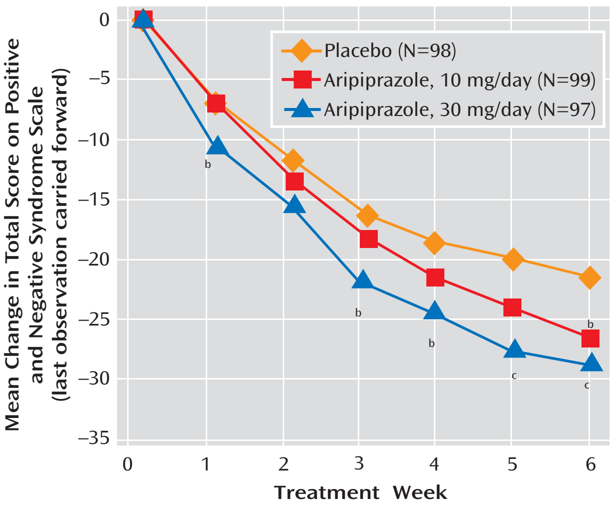

[a] The mean baseline total score for the entire group was 94.5. The numbers of subjects at week 1 were 97, 98, and 95 for the placebo, 10-mg, and 30-mg groups, respectively. The numbers at weeks 2–6 were 98, 99, and 97, respectively.

[b] Significantly different from value for placebo ($p<0.05$, t test derived from ANCOVA model with baseline value as covariate and treatment and regional strata as factors).

[c] Significantly different from value for placebo ($p<0.01$, t test derived from ANCOVA model with baseline value as covariate and treatment and regional strata as factors).

The data set at the day 42 visit, with the last observation carried forward, was used for the primary efficacy analysis. Scores on the Children's Global Assessment Scale, CGI severity scale, and PANSS positive and negative subscales were analyzed by fitting an ANCOVA model to the change scores with treatment, regional strata, and baseline value as right-hand terms. The same ANCOVA model was applied to the change in scores on the Simpson-Angus Scale, the Barnes Rating Scale for Drug-Induced Akathisia, and the AIMS. Scores on the CGI improvement scale and the overall score on the Pediatric Quality of Life Enjoyment and Satisfaction Questionnaire were analyzed by the Cochran-Mantel-Haenszel method, with regional strata as the stratification factor. Kaplan-Meier curves were plotted, and the log-rank test was used to test the significance of the difference in survival curves for time to discontinuation due to all reasons. Remission was defined as a score of 3 or less for items P1, P2, P3, N1, N4, N6, G5, and G9 of the PANSS, and the between-group differences in remission rate were analyzed by means of the Cochran-Mantel-Haenszel method, with regional strata as the stratification factor. The primary data set for analysis of scores on the Children's Global Assessment Scale, CGI severity and improvement scales, and PANSS positive and negative subscales was the last-observation-carried-forward data set at the day 42 visit. Both observed cases and the last observation carried forward were analyzed at all other visits. A significance level of 0.05 (two-tailed) was used for all secondary efficacy analyses to declare statistical significance. Analysis of change from baseline in PANSS total score was performed both for observed cases and the last observation carried forward at all visits before the day 42 visit, by means of methods similar to those already described.

Additionally, the patient dropout rates for each aripiprazole group and the placebo group were compared by using the Cochran-Mantel-Haenszel method stratified by regional strata. Statisti-

TABLE 2. Efficacy Data for Adolescents With Schizophrenia in a Placebo-Controlled Trial of Aripiprazole

Efficacy Measure	Placebo (N=98)		Aripiprazole, 10 mg/day (N=99)		Aripiprazole, 30 mg/day (N=97)		Comparison of Aripiprazole and Placebo (two-sided p)[a]	
	Mean	SE	Mean	SE	Mean	SE	10 mg/day	30 mg/day
Positive and Negative Syndrome Scale (PANSS) with last observation carried forward								
Total score								
Baseline	95.0	15.5	93.7	15.7	94.9	15.5	0.54	0.94
Change at week 1	–7.2	1.1	–6.9	1.1	–10.4	1.1	0.84	0.05
Change at week 6	–21.2	1.9	–26.7	1.9	–28.6	0.9	0.05	0.007
Positive subscale score								
Baseline	22.9	5.6	22.1	5.0	23.5	5.0	0.26	0.47
Change at week 1	–1.8	0.3	–2.1	0.3	–2.9	0.3	0.59	0.03
Change at week 6	–5.6	0.6	–7.6	0.6	–8.1	0.6	0.02	0.002
Negative subscale score								
Baseline	25.6	6.0	25.4	6.8	24.9	6.2	0.79	0.40
Change at week 1	–2.0	0.4	–1.5	0.3	–2.5	0.3	0.28	0.29
Change at week 6	–5.4	0.6	–6.9	0.6	–6.6	0.6	0.05	0.10
PANSS total score for observed cases								
Baseline	95.0	15.5	93.7	15.7	94.9	15.5	0.54	0.94
Change at week 1	–7.2	1.1	–6.9	1.1	–10.4	1.1	0.84	0.05
Change at week 6	–22.3	1.8	–30.6	1.8	–31.9	1.8	0.002	0.0002
Clinical Global Impression, with last observation carried forward								
Severity score								
Baseline	4.6	0.8	4.5	0.8	4.6	0.6	0.24	0.60
Change at week 1	–0.2	0.0	–0.2	0.0	–0.3	0.0	0.24	0.03
Change at week 6	–0.9	0.1	–1.2	0.1	–1.3	0.1	0.008	0.002
Improvement score[b]								
Week 1	3.8	0.1	3.6	0.1	3.4	0.1	0.02	0.002
Week 6	3.1	0.1	2.7	0.1	2.5	0.1	0.02	0.0004
Children's Global Assessment Scale score								
Baseline	45.4	11.2	46.7	12.6	45.6	12.0	0.43	0.87
Change at week 6	9.8	1.3	14.7	1.5	14.8	1.3	0.006	0.005
Pediatric Quality of Life Enjoyment and Satisfaction Questionnaire (29)[b]								
Total score								
Baseline	44.3	9.5	43.9	9.1	44.3	9.1	0.91	0.82
Change at week 6	4.5	0.9	5.2	0.9	5.9	0.9	0.55	0.26
Overall score[b]								
Baseline	3.4	0.9	3.2	0.9	3.3	1.1	0.48	0.27
Change at week 6	0.1	0.1	0.6	0.1	0.6	0.1	0.005	0.003
	N	%	N	%	N	%	10 mg/day	30 mg/day
Patients achieving remission with last observation carried forward[c]								
Week 1[d]	6	6	10	10	17	18	0.31	0.02
Week 6	35	36	53	54	56	58	0.02	0.003

[a] Least-squares means were used for the treatment comparisons except as noted. Two-tailed Student's t tests were used to test the difference between least-squares means within the ANCOVA model. For the baseline visit, analysis of variance was used with right-hand terms of treatment and regional strata as factors.
[b] Means were reported, and p values were derived by using the Cochran-Mantel-Haenszel method with mean score statistics stratified by regional strata.
[c] Remission was defined as a score not exceeding 3 (mild severity) for items P1, P2, P3, N1, N4, N6, G5, and G9 of the PANSS. Differences were analyzed by means of the Cochran-Mantel-Haenszel method with mean score statistics stratified by regional strata.
[d] Data were missing for four patients at the week 1 evaluation (placebo: N=97/98; 10 mg aripiprazole: N=98/99; 30 mg aripiprazole: N=95/97.

cal comparisons of mean changes in tolerability measures (e.g., extrapyramidal symptoms, metabolic variables, and ECG measures [QTcB interval]) yielding p values were performed by ANCOVA models on the respective change scores. The p values for comparing time to discontinuation from the study in each aripiprazole group and the placebo group were obtained by the log-rank test.

Results

Subject Characteristics and Disposition

Demographic characteristics of the 302 randomly assigned subjects are summarized in Table 1. During the fixed-dose period, the groups assigned to 10 and 30 mg/day of aripiprazole received mean daily doses of 9.8 mg and 28.9 mg, respectively. Of the 302 enrolled subjects, 258 (85%) completed the 6-week study. Among the 44 noncompleters (15%), 10 had been assigned to placebo, 16 to 10 mg/day of aripiprazole (nonsignificant difference from placebo, p=0.21), and 18 to 30 mg/day of aripiprazole (p=0.12 versus placebo). Of these, seven (2% of total) withdrew for lack of efficacy, 13 (4%) withdrew because of adverse events, 21 (7%) withdrew consent, one (0.0%) was lost to follow-up, and two (1%) withdrew for other reasons.

TABLE 3. Treatment-Emergent Adverse Events in Adolescents With Schizophrenia in a 6-Week Placebo-Controlled Trial of Aripiprazole

Adverse Event	Placebo (N=100)		Aripiprazole, 10 mg/day (N=100)		Aripiprazole, 30 mg/day (N=102)	
	N	%	N	%	N	%
Occurring in 5% or more of any group[a]						
Akathisia	5	5	5	5	12	12
Extrapyramidal disorder	5	5	13	13	22	22
Nausea	6	6	9	9	10	10
Vomiting	5	5	5	5	3	3
Dizziness	3	3	7	7	4	4
Headache	10	10	16	16	11	11
Somnolence	6	6	11	11	22	22
Tremor	2	2	2	2	12	12
Insomnia	15	15	11	11	10	10
Nasopharyngitis	4	4	5	5	5	5
Agitation	5	5	1	1	3	3
Extrapyramidal symptom categories[a]						
Dystonic event	0	0	4	4	2	2
Parkinsonism event (including extrapyramidal disorder and tremor)	7	7	15	15	31	30
Dyskinetic event	0	0	1	1	2	2
Akathisia event (including akathisia)	6	6	6	6	12	12
Residual event (myoclonus)	0	0	0	0	1	1

[a] Reported according to preferred terms in the Medical Dictionary for Regulatory Activities (34).

Efficacy

Treatment with both doses of aripiprazole resulted in significantly greater improvement than placebo between baseline and the end of treatment on the PANSS total (Figure 1, Table 2), PANSS positive symptom subscale, CGI severity measure, CGI improvement measure, and Children's Global Assessment Scale (Table 2). The 10-mg/day dose of aripiprazole also produced significantly greater results than placebo on the PANSS negative subscale (Table 2), whereas treatment with the 30-mg dose resulted in significant negative symptom improvement at weeks 3 and 4 (p=0.05 and p=0.05, respectively) but not at the end of the study. Separation from placebo occurred earlier for the group receiving 30 mg/day of aripiprazole (week 3) than for the 10-mg group (week 6). At the week 1 visit, the 30-mg group demonstrated significantly greater improvement than the placebo group on the PANSS total (Figure 1), PANSS positive subscale, and CGI severity and improvement scales (Table 2).

CGI improvement scores showed progressive improvement over the course of the study, with significant differences from placebo (Table 2) in the 10-mg aripiprazole group observed at weeks 1, 5, and 6 (p=0.02, p=0.03, p=0.02, respectively) and in the 30-mg group at all scheduled postbaseline visits except week 2 (week 1: p=0.002; weeks 3–6: p=0.005, p=0.004, p=0.0002, p=0.0004, respectively; last observation carried forward). Similarly, CGI severity scores showed improvement from "moderately"/"markedly" ill at baseline (scores of 4.6, 4.5, and 4.6 for the placebo, 10-mg, and 30-mg groups, respectively) to "mildly"/"moderately" ill at week 6 (score of 3.7 for placebo, 3.3 for both 10-mg and 30-mg groups) (last observation carried forward). Significant differences between placebo and aripiprazole were observed in the 10-mg group at weeks 3, 5, and 6 (p=0.04, p=0.03, p=0.008, respectively; last observation carried forward) and in the 30-mg group at all scheduled postbaseline visits except week 2 (week 1: p=0.03; weeks 3–6: p=0.003, p=0.02, p=0.004, p=0.002, respectively; last observation carried forward).

The mean change from baseline in the score on the Children's Global Assessment Scale at week 6 for both aripiprazole groups was significantly greater than the change with placebo (Table 2). On the Pediatric Quality of Life Enjoyment and Satisfaction Questionnaire Scale, both aripiprazole groups demonstrated significant improvement at the end of the study on the separate "overall score" item (prespecified in Method), but they did not show significant change from baseline on the 14-item "total score" (Table 2).

Treatment with both doses of aripiprazole resulted in significantly higher rates of remission at week 6 than was observed in the placebo group (Table 2). The rates were 54% for the patients taking 10 mg/day of aripiprazole, 58% for those taking 30 mg/day, and 36% for the placebo group.

Safety and Tolerability

Adverse events. Aripiprazole treatment was generally well tolerated and not dose limiting. There were no known suicides or deaths during the study. Forty-four (15%) of the 302 randomly assigned subjects left the study, 13 (4%) because of an adverse event: two in the placebo group (2%), seven in the group taking 10 mg/day of aripiprazole (7%), and four in the 30-mg aripiprazole group (4%). The most common adverse events associated with discontinuation were psychotic disorder (N=3) and schizophrenia (N=3); each of these six patients discontinued because of the respective adverse events rather than lack of efficacy. After demonstration of transient improvement in PANSS scores, each of the six patients was withdrawn from the study because of an event precipitated by exacerbation of symp-

TABLE 4. Metabolic Effects in Adolescents With Schizophrenia in a 6-Week Placebo-Controlled Trial of Aripiprazole

	Placebo					10 mg/day of Aripiprazole				
		Data					Data			
Metabolic Variable	N	Mean	SD	N	%	N	Mean	SD	N	%
Body weight										
Change from baseline	98					99				
Absolute (kg)		–0.8	2.6				0.0	2.1		
Percent loss	51					37				
>0% to ≤2%				13	13				17	17
>2% to ≤5%				27	28				15	15
>5%				11	11				5	5
Percent gain	47					62				
≥0% to <2%				35	36				36	36
≥2% to <5%				10	10				15	15
≥5%				2	2				11	11
Change in z score[a]	98	–0.11	0.22			99	0.0	0.28		
Body mass index	98					99				
Change from baseline (kg/m^2)		–0.3	1.0				–0.0	0.8		
Change in z score[b]		–0.12	0.27				0.01	0.26		
Fasting serum glucose level										
Change from baseline (mg/dl)[c]	65	–3.15	14.43			62	2.10	12.65		
Clinically meaningful elevation (110 mg/dl or above)	88			2	2	86			2	2
Lipid levels[d]										
Change in total cholesterol (mg/dl)	96	–10.38	24.08			98	–7.43	27.99		
Change in fasting triglycerides (mg/dl)[e]	45	–6.51	53.82			47	–4.53	48.72		
Change in fasting high-density lipoprotein cholesterol (mg/dl)[e]	45	–6.09	20.23			47	0.13	6.99		
Change in prolactin level (ng/ml)[f]	96	–8.45	24.23			98	–11.93	23.29		

[a] Ranges are as follows: placebo, –0.98 to 0.39; 10 mg aripiprazole, –1.31 to 1.54; 30 mg aripiprazole, –0.76 to 0.46.
[b] Ranges are as follows: placebo, –0.99 to 0.56; 10 mg aripiprazole, –1.08 to 1.07; 30 mg aripiprazole, –1.07 to 0.80.
[c] With the last observation carried forward; based on the number of subjects who were fasting and who completed the baseline assessment and at least one postbaseline assessment.
[d] With the last observation carried forward.
[e] From a subgroup of subjects who used central laboratories.
[f] Median values are as follows: placebo, –1.0 ng/ml; 10 mg aripiprazole, –4.0 ng/ml; 30 mg aripiprazole, –6.0 ng/ml.

toms consistent with schizophrenia or psychosis. Five patients were receiving active treatment with aripiprazole (three taking 10 mg/day, two taking 30 mg/day), and one subject was receiving placebo. No other adverse event was reported by more than one patient among those who left the study.

The most frequently reported treatment-emergent adverse events that occurred at an incidence of 5% or higher in any group are shown in Table 3. The most common adverse events associated with aripiprazole (more than 5% in either aripiprazole group and a combined incidence at least twice that for placebo) were extrapyramidal disorder, somnolence, and tremor. The incidence of these adverse events appeared to be consistently higher at the 30-mg dose than at the 10-mg dose. The rates of serious treatment-emergent adverse events were low for all groups, with an incidence of 3% in the placebo group, 4% in the 10-mg aripiprazole group, and 4% in the 30-mg aripiprazole group.

Extrapyramidal symptoms. Adverse events involving extrapyramidal symptoms other than akathisia were mild to moderate in severity; one patient in the 10-mg aripiprazole group dropped out of the study because of dystonia. Individual treatment-emergent extrapyramidal symptoms are listed in Table 3. The most common (more than 5% incidence) were extrapyramidal disorder, akathisia, and tremor. Both doses of aripiprazole showed slight worsening from baseline on the Simpson-Angus Scale. The score for placebo fell by 0.3 points, whereas the group taking 10 mg/day of aripiprazole had an increase of 0.5 and those taking 30 mg/day had an increase of 0.3. The difference from the change with placebo was significant for both the 10-mg (p=0.007) and 30-mg (p=0.05) doses. There were no statistically significant differences between either dose of aripiprazole and placebo on either the Barnes Rating Scale for Drug-Induced Akathisia or AIMS at study endpoint.

Metabolic variables. Results of metabolic measurements are summarized in Table 4. Patients in the placebo group lost an average of 0.8 kg over the 6-week treatment period, which was significantly different from the changes in the aripiprazole-treated patients, who either had no overall change in weight (10-mg group) or gained 0.2 kg on average (30-mg group) (p=0.009). There were no clinically significant differences among treatment groups in glucose or lipid measures.

Other clinical laboratory evaluations and vital signs. There were no clinically relevant changes in heart rate or blood pressure during the course of the study. The most common potentially clinically significant laboratory test abnormality was elevation of total creatine phosphokinase, defined as an increase of 500 U/liter or more from baseline. A total of 17 subjects showed a po-

30 mg/day of Aripiprazole				
	Data			
N	Mean	SD	N	%
97				
	0.2	2.3		
35				
			16	16
			12	12
			7	7
62				
			36	37
			17	18
			9	9
97	0.0	0.20		
97				
	0.0	0.8		
	0.01	0.25		
55	–0.98	10.57		
79			0	0
95	–5.01	23.28		
40	–0.73	51.85		
40	0.05	7.92		
92	–15.14	26.87		

tentially clinically significant elevation: five placebo-treated subjects, five patients receiving 10 mg/day of aripiprazole, and seven receiving 30 mg/day of aripiprazole.

All groups showed mean decreases in serum prolactin after 6 weeks of treatment (Table 4). Both active treatment groups had significantly greater reductions in prolactin than did the placebo group (10-mg aripiprazole, p=0.003; 30-mg aripiprazole, p<0.0001). While the mean change from baseline in the 30-mg aripiprazole cohort was numerically greater than that observed in the 10-mg group, this difference was not statistically significant (p=0.16). Low prolactin levels are defined as those below 3 ng/ml for females and below 2 ng/ml for males. According to these definitions, the rates of low prolactin levels were 8% for the placebo group, 34% for the group taking 10 mg/day of aripiprazole (significantly different from placebo, p<0.0001), and 26% for those taking 30 mg/day of aripiprazole (p=0.001).

No trends were observed for any potentially clinically significant changes in ECG measures, including QTcB interval. At the last visit, the mean changes in QTcB intervals were –1.3 msec (SD=15.6) for placebo, 0 msec (SD=14.0) for the 10-mg dose of aripiprazole, and –5.4 msec (SD=23.4) for 30 mg of aripiprazole.

Time to Discontinuation

No statistically significant differences were observed between placebo and aripiprazole with respect to time to discontinuation due to all reasons (10 mg versus placebo: hazard ratio, 1.67, p=0.20; 30 mg versus placebo: hazard ratio, 1.88, p=0.11).

Discussion

To our knowledge, this study with aripiprazole is the largest investigation conducted to date in adolescents with schizophrenia. Aripiprazole at doses of 10 and 30 mg/day was more efficacious in ameliorating the symptoms of schizophrenia than was placebo, as demonstrated by significantly greater improvements on the PANSS total score. Although considerable improvement was also observed with placebo, the salutary effects of placebo highlight the utility of employing a placebo-controlled methodology in treatment studies with this vulnerable adolescent population.

Statistically significant superiority for both active treatment arms was also demonstrated on the PANSS positive subscale scores at week 6. In addition, decreases were seen in PANSS negative subscale scores in both aripiprazole dosage groups. However, a statistically significant difference from placebo was observed in the 10-mg aripiprazole arm at study endpoint only. The 30-mg aripiprazole dose was superior to placebo at weeks 3 and 4 but not at endpoint (week 6 or last observation). End-of-study scores for the CGI severity and improvement scales demonstrated improvement consistent with a clinically meaningful response, with both dose groups showing significant reductions over the course of the study. Subjects with a “moderately” to “markedly” severe baseline illness (mean CGI severity score, 4.6) demonstrated clinically meaningful improvement, as shown by the CGI improvement scale. Both dose groups demonstrated statistically significant improvement compared to placebo, and the difference from placebo was numerically greater for the 30-mg group than for the 10-mg group at all scheduled postbaseline visits except week 2. Finally, after 6 weeks of treatment at either dose, scores on the Children’s Global Assessment Scale improved by more than 30% in both aripiprazole groups, compared with 22% in the placebo group.

The rate of discontinuation due to adverse events was low in both aripiprazole groups and was comparable to the rate in the placebo group. One patient in the 10-mg aripiprazole group withdrew owing to the emergence of dystonia, which resolved 3 days later (26).

While neither active treatment group exhibited substantial weight gain in the current study, the change in weight differed across groups, because of weight loss in the placebo group but not in the two active treatment groups. Thus, like the data for other antipsychotic medications, findings from the current trial suggest that clinicians treating adolescents should remained concerned about

the potential for adverse long-term changes in weight with aripiprazole.

Hyperprolactinemia also has long been reported with antipsychotic therapy. The effects of hyperprolactinemia—anovulation, amenorrhea, decreased libido, orgasmic dysfunction, breast engorgement, galactorrhea, and hypoestrogenism/androgenism—can be disruptive and may be more pronounced in postpubertal adolescents (35). All treatment groups demonstrated a reduction from baseline in serum prolactin concentrations. Significant differences between placebo and aripiprazole may be related to the dopamine partial agonist properties of aripiprazole, which is thought to mimic the inhibitory action of dopamine on pituitary prolactin secretion. Few published data exist on the consequences of lowered prolactin in adult or in child and adolescent populations; however, possible consequences include failure to lactate in childbearing girls and possible decrease in pubic hair. In cases of extremely low prolactin (e.g., less than 2 ng/ml), there is the possibility of impaired fertility (36–39).

Despite the observation that overall clinical improvement has been demonstrated in adolescents with schizophrenia who receive placebo, and the lack of approved treatments for adolescent schizophrenia at the time this study began, ethical concerns about the use of placebo in clinical studies remain. In an attempt to reduce the risk associated with participation in this clinical trial, subjects were allowed to receive “rescue medication” (benzodiazepines and anticholinergics) over the course of the study. In addition, subjects who received placebo were given the option to receive aripiprazole at the end of the study. Furthermore, as a precautionary measure, a data safety monitoring board reviewed and evaluated cumulative safety data collected at regular intervals to ensure the safety of all subjects enrolled in this study.

The procedures used in this study addressed the methodologic limitations of most other studies of pharmacologic therapy in adolescent schizophrenia. Nevertheless, because schizophrenia generally requires long-term treatment, additional large, randomized, placebo-controlled clinical trials are needed to assess long-term outcomes. In addition, the study did not include patients with schizoaffective disorder; thus, caution should be used in applying these study results to that patient group. Finally, large-scale, randomized, head-to-head trials of typical versus atypical antipsychotics in adolescents are necessary so that between-drug comparisons can be made on the basis of methodologically stringent data.

In conclusion, aripiprazole was generally well tolerated, with a low rate of discontinuation due to adverse events and a high completion rate in adolescents with schizophrenia. These results provide support for aripiprazole use in adolescents with schizophrenia at a starting dose of 2 mg/day, and they provide evidence of efficacy and tolerability at the recommended adult doses, ranging from 10 to 30 mg/day. Longer-term, randomized, head-to-head trials of typical versus atypical antipsychotics are necessary to confirm the efficacy and safety of long-term management of schizophrenia in adolescents.

Received June 29, 2007; revisions received Dec. 14, 2007, and May 5 and June 5, 2008; accepted June 6, 2008 (doi: 10.1176/appi.ajp.2008.07061035). From the Department of Child and Adolescent Psychiatry, Case Western Reserve University; University Hospitals Case Medical Center, Cleveland; the Division of Child and Adolescent Psychiatry, Children’s National Medical Center, Washington, D.C.; and Otsuka Pharmaceutical Company, Rockville, Md. Address correspondence and reprint requests to Dr. Findling, Department of Child and Adolescent Psychiatry, University Hospitals Case Medical Center, Case Western Reserve University School of Medicine, 10524 Euclid Ave., Cleveland, OH 44106-5080; robert.findling@UHhospitals.org (e-mail).

Dr. Findling receives or has received research support from, acted as a consultant to, and/or served on a speaker’s bureau for Abbott, AstraZeneca, Bristol-Myers Squibb, Celltech-Medeva, Cypress Biosciences, Forest, GlaxoSmithKline, Johnson & Johnson, Eli Lilly, New River, Novartis, Otsuka, Pfizer, Sanofi-Aventis, Sepracore, Shire, Solvay, Supernus Pharmaceuticals, and Wyeth.

Dr. Robb receives or has received research support from, acted as a consultant to, and/or served on a speaker’s bureau for Abbott, Janssen, Forest, McNeil, Eli Lilly, Lundbeck, Pfizer, Otsuka, Bristol-Myers Squibb, the National Institute of Child Health and Human Development, and NIMH.

Dr. Nyilas, Dr. Ivanova, Mr. Jin, Dr. Forbes, Dr. McQuade, Dr. Iwamoto, and Dr. Carson are employees of Otsuka.

Dr. Marcus is an employee of Bristol-Myers Squibb.

Supported by Otsuka Pharmaceutical Co., Ltd.

Clinical trial registry number: NCT00102063 (ClinicalTrials.gov).

References

1. Burd L, Kerbeshian J: A North Dakota prevalence study of schizophrenia presenting in childhood. J Am Acad Child Adolesc Psychiatry 1987; 26:347–350
2. Hafner H, Nowotny B: Epidemiology of early-onset schizophrenia. Eur Arch Psychiatry Clin Neurosci 1995; 245:80–92
3. Loranger AW: Sex difference in age at onset of schizophrenia. Arch Gen Psychiatry 1984; 41:157–161
4. Thomsen PH: Schizophrenia with childhood and adolescent onset—a nationwide register-based study. Acta Psychiatr Scand 1996; 94:187–193
5. American Academy of Child and Adolescent Psychiatry: Practice Parameter for the Assessment and Treatment of Children and Adolescents With Schizophrenia. J Am Acad Child Adolesc Psychiatry 2001; 40(July suppl)
6. McClellan JM, Werry JS, Ham M: A follow-up study of early onset psychosis: comparison between outcome diagnoses of schizophrenia, mood disorders, and personality disorders. J Autism Dev Disord 1993; 23:243–262
7. Krausz M, Muller-Thomsen T: Schizophrenia with onset in adolescence: an 11-year followup. Schizophr Bull 1993; 19:831–841
8. Cawthron P, James A, Dell J, Seagroatt V: Adolescent onset psychosis: a clinical and outcome study. J Child Psychol Psychiatry 1994; 35:1321–1332
9. Gillberg IC, Hellgren L, Gillberg C: Psychotic disorders diagnosed in adolescence: outcome at age 30 years. J Child Psychol Psychiatry 1993; 34:1173–1185
10. Pool D, Bloom W, Mielke DH, Roniger JJ Jr, Gallant DM: A controlled evaluation of Loxitane in seventy-five adolescent schizophrenic patients. Curr Ther Res Clin Exp 1976; 19:99–104
11. Kryzhanovskaya L, Schulz C, McDougle CJ, Frazier JA, Shen J, Dittman R, Robertson-Plouch C, Bauer T, Xu W, Wang WV, Carlson J, Corya S, Tohen M: A double-blind, placebo-controlled

study of olanzapine in adolescents with schizophrenia. Neuropsychopharmacology 2005; 30(suppl 1):S258–S259
12. Cutler AJ, Marcus RN, Hardy SA, O'Donnell A, Carson WH, McQuade RD: The efficacy and safety of lower doses of aripiprazole for the treatment of patients with acute exacerbation of schizophrenia. CNS Spectr 2006; 11:691–702
13. Kane JM, Carson WH, Saha AR, McQuade RD, Ingenito GG, Zimbroff DL, Ali MW: Efficacy and safety of aripiprazole and haloperidol versus placebo in patients with schizophrenia and schizoaffective disorder. J Clin Psychiatry 2002; 63:763–771
14. Pigott TA, Carson WH, Saha AR, Torbeyns AF, Stock EG, Ingenito GG: Aripiprazole for the prevention of relapse in stabilized patients with chronic schizophrenia: a placebo-controlled 26-week study. J Clin Psychiatry 2003; 64:1048–1056
15. Potkin SG, Saha AR, Kujawa MJ, Carson WH, Ali M, Stock E, Stringfellow J, Ingenito G, Marder SR: Aripiprazole, an antipsychotic with a novel mechanism of action, and risperidone vs placebo in patients with schizophrenia and schizoaffective disorder. Arch Gen Psychiatry 2003; 60:681–690
16. Keepers GA, Clappison VJ, Casey DE: Initial anticholinergic prophylaxis for neuroleptic-induced extrapyramidal syndromes. Arch Gen Psychiatry 1983; 40:1113–1117
17. Safer DJ: A comparison of risperidone-induced weight gain across the age span. J Clin Psychopharmacol 2004; 24:429–436
18. World Medical Association: World Medical Association Declaration of Helsinki: Ethical Principles for Medical Research Involving Human Subjects. Helsinki, World Medical Association, 1964, pp 1–5
19. World Health Organization: Declaration of Helsinki: recommendations guiding physicians in biomedical research involving human subjects. BMJ 1996; 313:1448–1449
20. Kay SR: Positive and Negative Syndromes in Schizophrenia: Research and Assessment. New York, Brunner/Mazel, 1991
21. Kay SR, Fiszbein A, Opler A: The Positive and Negative Syndrome Scale for Schizophrenia (PANSS). Schizophr Bull 1987; 13:261–276
22. American Psychiatric Association: Diagnostic and Statistical Manual of Mental Disorders, 4th ed (DSM-IV). Arlington, Va, American Psychiatric Press, 2000
23. Kaufman J, Birmaher B, Breslau D, Rao U, Ryan N: Kiddie-SADS—Present and Lifetime Version (K-SADS-PL). Pittsburgh, University of Pittsburgh, School of Medicine, Oct 1996 (http://www.wpic.pitt.edu/ksads/ksads-pl.pdf)
24. Findling RL, Blumer JL, Kauffman R, Batterson JR, Gilbert DL, Bramer S, Marcus R: Pharmacokinetic effects of aripiprazole in conduct disorder, in Scientific Proceedings of the 50th Annual Meeting of the American Academy of Child and Adolescent Psychiatry. Washington, DC, AACAP, 2003
25. Findling RL, Kauffman R, Sallee FR, Auby P, Nyilas M, Mallikaarjun S, Forbes RA, Marcus RM, Carson WH: Safety, tolerability, and pharmacokinetics of aripiprazole in children and adolescents with major psychiatric diagnoses, in 2006 Annual Meeting New Research Program and Abstracts. Arlington, VA, American Psychiatric Association, 2006, number 653
26. Findling RL, Kauffman R, Sallee FR, Auby P, Nyilas M, Mallikaarjun S, Forbes RA, Marcus RM, Carson WH: Effectiveness of aripiprazole in children and adolescents with major psychiatric diagnoses, in 2006 Annual Meeting New Research Program and Abstracts. Arlington, VA, American Psychiatric Association, 2006, number 654
27. Guy W, Bonato R (eds): Manual for the ECDEU Assessment Battery, 2nd ed. Chevy Chase, Md, National Institute of Mental Health, 1970, pp 12-1–12-6
28. Shaffer D, Gould MS, Brasic J, Ambrosini P, Fisher P, Bird H, Aluwahlia S: A children's global assessment scale (CGAS). Arch Gen Psychiatry 1983; 40:1228–1231
29. Endicott J, Nee J, Yang R, Wohlberg C: Pediatric Quality of Life Enjoyment and Satisfaction Questionnaire (PQ-LES-Q): reliability and validity. J Am Acad Child Adolesc Psychiatry 2006; 45: 401–407
30. Simpson GM, Angus JW: A rating scale for extrapyramidal side effects. Acta Psychiatr Scand Suppl 1970; 212:11–19
31. Barnes TR, Braude WM: Akathisia variants and tardive dyskinesia. Arch Gen Psychiatry 1985; 42:874–878
32. Guy W (ed): ECDEU Assessment Manual for Psychopharmacology: Publication ADM 76-338. Washington, DC, US Department of Health, Education, and Welfare, 1976, pp 534–537
33. Poznanski EO, Freeman LN, Mokros HB: Children's Depression Rating Scale—Revised. Psychopharmacol Bull 1984; 21:979–989
34. Brown EG, Wood L, Wood S: The medical dictionary for regulatory activities (MedDRA). Drug Saf 1999; 20:109–117
35. Correll CU, Carlson HE: Endocrine and metabolic adverse effects of psychotropic medications in children and adolescents. J Am Acad Child Adolesc Psychiatry 2006; 45:771–791
36. Dalzell GW, Atkinson AB, Carson DJ, Sheridan B: Normal growth and pubertal development during bromocriptine treatment for a prolactin-secreting pituitary macroadenoma. Clin Endocrinol (Oxf) 1987; 26:169–172
37. Gillam MP, Molitch ME, Lombardi G, Colao A: Advances in the treatment of prolactinomas. Endocr Rev 2006; 27:485–534
38. Kauppila A, Martikainen H, Puistola U, Reinila M, Ronnberg L: Hypoprolactinemia and ovarian function. Fertil Steril 1988; 49: 437–441
39. Schulz KD, Geiger W, del PE, Kunzig HJ: Pattern of sexual steroids, prolactin, and gonadotropic hormones during prolactin inhibition in normally cycling women. Am J Obstet Gynecol 1978; 132:561–566
