# Supplementary material for: Dose-Dependent Efficacy of Aripiprazole in Treating Patients With Schizophrenia or Schizoaffective Disorder: A Systematic Review and Meta-Analysis of Randomized Controlled Trials
Source: Front Psychiatry. 2021 Aug 11;12:717715. doi: 10.3389/fpsyt.2021.717715 (PMC8385236; doi:10.3389/fpsyt.2021.717715)
Supplement: Supplementary file 1 [file Data_Sheet_1.ZIP › supplementray material-9 RCTs/2 Casey 2003.pdf]

Psychopharmacology (2003) 166:391–399
DOI 10.1007/s00213-002-1344-3

ORIGINAL INVESTIGATION

Daniel E. Casey · William H. Carson · Anutosh R. Saha · Amy Liebeskind · Mirza W. Ali · Darlene Jody · Gary G. Ingenitoon behalf of the Aripiprazole Study Group

Switching patients to aripiprazole from other antipsychotic agents: a multicenter randomized study

Received: 23 May 2002 / Accepted: 6 November 2002 / Published online: 28 February 2003
© Springer-Verlag 2003

Abstract *Rationale:* Switching patients from one antipsychotic to another can lead to tolerability problems or transient symptom exacerbations. It is important to compare switching strategies to determine which methods produce the best possible patient outcomes. *Objective:* To investigate the efficacy, safety and tolerability of three dosing strategies for switching chronic, stable patients with schizophrenia from current oral antipsychotic monotherapy to once-daily oral aripiprazole monotherapy. *Method:* Patients in this 8-week, open-label, outpatient study were randomized to: 1) immediate initiation of 30 mg/day aripiprazole with simultaneous immediate discontinuation of current antipsychotic; 2) immediate initiation of 30 mg/day aripiprazole while tapering off current antipsychotic over 2 weeks; or 3) up-titrating aripiprazole to 30 mg/day over 2 weeks, while simultaneously tapering off current antipsychotic. Efficacy assessments included PANSS, CGI-S, and CGI-I scores. Safety assessments included: adverse events (AEs) recording, evaluation of extrapyramidal symptoms (EPS), vital signs, ECG, and clinical laboratory tests.

Results Efficacy with aripiprazole was maintained during the study with numerical improvements compared with baseline in all three groups. The overall incidence of AEs was broadly comparable across all groups, and AEs were generally mild to moderate in severity and time-limited. Discontinuations due to AEs were comparable across the groups. No deterioration in EPS occurred in any group. The reduction in body weight and plasma prolactin levels following switch to aripiprazole were comparable across the three groups. *Conclusion:* Any of the three strategies evaluated can be used safely for switching patients to aripiprazole from antipsychotic monotherapy. Furthermore, patients' symptoms may continue to improve after switching to aripiprazole.

Keywords Aripiprazole · Schizophrenia · Atypical antipsychotic · Switching

D. E. Casey (✉)
Mental Health Division (P3MIRECC),
VA Medical Center, 3710 SW US Veterans Hospital Road,
Portland, OR 97239, USA
e-mail: daniel.casey@med.va.gov
Tel.: +1-503-2208262
Fax: +1-503-2735211

W. H. Carson
Otsuka America Pharmaceutical Inc,
Princeton, NJ 08540, USA

A. R. Saha · M. W. Ali · G. G. Ingenito
Otsuka Maryland Research Institute,
Rockville, MD 20850, USA

A. Liebeskind
Lenox Hill Hospital, New York, NY 10021, USA

D. Jody
Bristol-Myers Squibb, Princeton, NJ 08543, USA

Introduction

Schizophrenia is a devastating, heterogeneous, chronic disease that deprives afflicted individuals of active, satisfying participation in social interactions and work. In addition to a drastically impaired quality of life for the affected individual, the disease places huge demands and strains on the networks of family and friends who provide care and support for patients with schizophrenia. Patients often require long-term, highly intensive treatment and care, with a considerable impact on medical resources and associated cost. In the United States, schizophrenia is estimated to cost over $40 billion a year when indirect costs such as lost productivity are taken into consideration (Bunney and Meltzer 1995).

At present, antipsychotic medication is the mainstay of therapy for schizophrenia. The current pharmacologic armamentarium includes both typical and atypical antipsychotics. Typical agents are associated with efficacy in terms of positive symptoms in some patients, but have limited efficacy against negative symptoms. In addition, at least 20% of patients do not demonstrate a response to typical agents (Davis et al. 1980). Furthermore, side effect liabilities, including extrapyramidal symptoms (EPS) and

hyperprolactinemia, can limit patients' ability to tolerate and maintain treatment. Atypical agents have been suggested to be more effective against negative symptoms and to produce a response in a subset of patients not responsive to typical agents, but are also associated with potentially treatment-limiting side effects including weight gain, hyperprolactinemia, somnolence, QT_c prolongation and agranulocytosis (Lieberman et al. 1988; Fleischhacker et al. 1994; Casey 1997; Kurzthaler and Fleischhacker 2001).

Inadequate response and lack of tolerability are the two main reasons clinicians switch patients from treatment with one antipsychotic to another. Response rates with typical agents range from 60 to 70% (Kane 1987). For patients not responding to typical antipsychotic therapy, switching to an atypical agent is recommended (Remington and Chong 1999), as studies have shown that treatment with another conventional antipsychotic often proves ineffective. One study showed that unresponsiveness to these agents appeared to occur across the class (Kolakowska et al. 1985). In a more recent study (Kinon et al. 1993), patients showing a poor response to fluphenazine also responded poorly when switched to haloperidol.

Variations are also observed between patients in their response to different atypical agents. Prior studies have shown that switching to atypicals may be associated with enhanced response rates (Remington and Chong 1999; Dossenbach et al. 2000, 2001). Variations in the response of patients to the newer atypicals are thought to relate to the differing pharmacological activity of these agents. In addition, variations are observed between patients in their tolerability to different atypical agents. For example, studies involving patients responsive to clozapine treatment but experiencing medication-induced adverse events have shown that switching to olanzapine can reduce drug-induced side effects while maintaining symptom control (Littrell et al. 2000).

One of the main problems associated with switching antipsychotic agents is the potential for adverse risks upon discontinuation. The process of switching antipsychotic agents can result in a re-emergence or worsening of psychosis, along with unpleasant side effects such as insomnia, nausea, vomiting, anxiety and agitation (Kinon et al. 2000; Peuskens 2000). Studies systematically investigating strategies for switching from one antipsychotic to another are limited and the conclusions have been mixed. Some studies have shown that abrupt withdrawal of the current antipsychotic may be possible for some patients, while others may need a gradual reduction in dose before the new antipsychotic is initiated. Other studies have suggested that the switch is best made by overlapping existing therapy and the new antipsychotic (Kinon et al. 2000; Peuskens 2000).

The current study investigates strategies for switching patients from their current antipsychotic therapy to aripiprazole, a newly developed antipsychotic with a unique mechanism of action. Unlike currently available antipsychotics, all of which are dopamine D_2 receptor antagonists, aripiprazole has a unique combination of dopamine and serotonin receptor-binding affinities and functional activities (Burris et al. 2000; Jordan et al. 2001). Aripiprazole is a potent dopamine D_2 partial agonist; it blocks D_2 receptors under hyperdopaminergic conditions, but under hypodopaminergic conditions, agonist properties are evident (Kikuchi et al. 1995; Inoue et al. 1996; Burris et al. 2000). At serotonin 5-HT_{1A} receptors, aripiprazole is a potent partial agonist (Jordan et al. 2001). Partial agonism at 5-HT_{1A} receptors has been associated with anxiolytic efficacy (Glennon and Dukat 1995), and 5-HT_{1A} partial agonists have been reported to reduce stress-induced psychosocial deficits in patients with chronic schizophrenia (Jan 1988; Sovner and Parner-Sovner 1989). Preclinical studies have demonstrated that aripiprazole is a serotonin 5-HT_{2A} receptor antagonist (unpublished data). This combination of partial agonist activity at 5-HT_{1A} receptors (Millan 2000) and antagonist activity at 5-HT_{2A} receptors (Leysen et al. 1993) is postulated to result in favorable effects on the negative symptoms of schizophrenia.

The above studies suggest that aripiprazole may function as a dopamine-serotonin system stabilizer, acting as a functional antagonist or functional agonist at dopamine and serotonin receptors depending on the level of the relevant neurotransmitter in the immediate environment.

Earlier clinical studies have examined the efficacy, safety and tolerability of aripiprazole in patients with schizophrenia or schizoaffective disorder (Carson et al. 2001; Saha et al. 2001; Yeung et al. 2001; Kane et al. 2002). In placebo-controlled studies, aripiprazole was shown to be effective in improving both the positive and negative symptoms of these disorders. Furthermore, aripiprazole treatment was associated with an excellent safety and tolerability profile, with minimal liability for the side effects that limit treatment with other agents, including EPS, elevated prolactin, weight gain, sedation, and QT_c prolongation (Carson et al. 2001, 2002; Yeung et al. 2001). These results indicate considerable potential for aripiprazole in the treatment of psychotic disorders and are consistent with the effects predicted from the drug's unique pharmacologic profile.

The present 8-week, open-label, randomized study examined the relative efficacy, safety and tolerability of three different strategies for switching stable outpatients from prior antipsychotic monotherapy to aripiprazole 30 mg/day monotherapy.

Materials and methods

Organization of the study and selection of patients

This study ran in accordance with the principles of the Declaration of Helsinki, Good Clinical Practice and complied with Code of Federal Regulations for Institutional Review Board (IRB) and independent ethics committees. All patients were required to provide informed, written consent prior to participation in the

study, which was co-signed by their next of kin or caregiver as required by the local IRB.

For inclusion in the study, patients were required to be between 18 and 65 years of age. Both men and women were eligible. All patients had to have a confirmed diagnosis of schizophrenia or schizoaffective disorder (DSM-IV). Women could not be pregnant, and were required to be using adequate contraceptive measures. Patients were also required to be chronic and stable in terms of their disease condition, as defined by taking a stabilized dose of a single oral typical antipsychotic (haloperidol or thioridazine), or atypical antipsychotic (risperidone or olanzapine) for at least 1 month prior to study entry. In addition, the study protocol only states that there must have been an adequate clinical reason for the patient to try a new medication (i.e. aripiprazole) other than current therapy. As stated in the exclusion criteria, patients could not have been hospitalized for an exacerbation of schizophrenia or schizoaffective disorder for at least 2 months. There is no further information or data that was collected on the study report forms detailing the exact reason the investigator felt it necessary to switch to aripiprazole.

Exclusion criteria included: any patient hospitalized for acute exacerbation of their condition within 2 months of randomization; judged to be at risk of suicide or with a recent history of alcohol or psychoactive substance dependence; who had taken a selective serotonin reuptake inhibitor (SSRI) within 4 weeks of screening; and any patient with a psychiatric diagnosis other than schizophrenia or schizoaffective disorder that required pharmacotherapy. Patients were also to be excluded if they had any of the following neurologic diagnoses: migraine, epilepsy, Parkinson's disease, Alzheimer's disease, multiple sclerosis, episodes of stroke or transient ischemic attacks, cerebral palsy, or any condition manifested by abnormality at neurological examination. Patients with tardive dyskinesia or symptoms of extrapyramidal syndrome due solely to their current or prior use of antipsychotic medication were not to be excluded. Patients were to be excluded if they had an acute or unstable medical condition or somatic condition, whose symptoms could masquerade as signs or symptoms of psychosis or as adverse events of antipsychotic medication. Patients were also to be excluded if they had taken an investigational drug within 4 weeks prior to randomization. In addition, patients who had received aripiprazole in a prior study, whose current antipsychotic medication was an injectable long-acting agent, or who were required to take, or might need to take, medication that inhibits the microsomal enzyme CYP2D6 or inhibits or acts as a substrate for CYP3A4, were to be excluded.

Study design and randomization to treatment

This randomized, open-label, parallel-group, 8-week, outpatient study was conducted at 29 centers in the United States. Patients were randomized to one of the three aripiprazole treatment groups via a centralized telephone call-in system: group 1: immediate initiation of once-daily 30 mg oral aripiprazole with simultaneous immediate discontinuation of current antipsychotic monotherapy; group 2: immediate initiation of once-daily 30 mg aripiprazole while tapering off the current antipsychotic monotherapy over the first 14 days; group 3: titrating oral aripiprazole upwards (from 10 mg/day during week 1, to 20 mg/day during week 2 and starting week 3 with 30 mg/day), while tapering off the current antipsychotic medication over the same 2-week period, then maintaining the patient at 30 mg/day oral aripiprazole. Tapering of current antipsychotic medication was to be performed as follows: during week 1, the dose of current antipsychotic medication was to be decreased to half the previous week's dose; during week 2, the dose of current antipsychotic medication was to be decreased to half the dose during week 1, and at the start of week 3, the current antipsychotic medication was to be totally discontinued. All aripiprazole treatment was administered as 10 mg tablets in a single morning dose after breakfast. No dosage adjustments during the study were permissible.

The primary objective of this study was to assess the relative safety and tolerability of three different dosing schemes for switching patients from prior antipsychotic monotherapy to aripiprazole monotherapy. Secondary objectives were to determine the continued efficacy and safety, compared to prior therapy, of aripiprazole over the 8-week period. The aripiprazole dose of 30 mg/day was selected for the study as this dose has been shown to be clinically effective and is the highest dose that has been examined in clinical efficacy studies.

Efficacy assessments

Efficacy was assessed using the Positive and Negative Syndrome Scale (PANSS) (Kay et al. 1987) at baseline and the end of weeks 4 and 8. Assessments included the PANSS total score, the PANSS positive subscale score (seven positive symptom constructs) and the PANSS negative subscale score (seven negative symptom constructs). Each of the subscales rates the severity of each symptom construct on a 7-point scale. The Clinical Global Impression (CGI) Scale (Guy 1976b) was also used to assess efficacy and consists of two 7-point subscales, the Severity of Illness Scale (CGI-S) and the Global Improvement Scale (CGI-I). The CGI-S was performed at baseline, and subsequently, CGI-I and CGI-S assessments were conducted at each post-baseline visit and at discontinuation (if applicable). Time to discontinuation was also assessed as a further measure of efficacy.

Safety assessments

Adverse events (AEs) were elicited by direct, non-prompted questioning by the investigator at each post-baseline visit and classified by body system using Coding Symbols for Thesaurus of Adverse Reaction Terms (COSTART) terminology. AEs were assessed as mild, moderate or severe. In this study, worsening of schizophrenia was recorded as an AEs. The relationship of each AEs to study medication was assessed by investigators as probable, possible, or none. Serious AEs were defined as life-threatening, resulting in death, persistent or significant disability, or requiring hospitalization.

Potential EPS-related side effects (e.g. parkinsonism, dyskinesia, and akathisia) were evaluated using three standardized rating instruments: the Simpson-Angus Scale (SAS) (Simpson and Angus 1970), the Barnes Akathisia Rating Scale (BAS) (Barnes 1989), and the Abnormal Involuntary Movement Scale (AIMS) (Guy 1976a). These scales were completed by the investigator at baseline and at each scheduled visit thereafter (end of weeks 1, 2, 3, 4, 6, and 8).

Vital signs (blood pressure and heart rate) were measured at screening, baseline, and all scheduled visits. Twelve-lead ECGs were performed at screening and at the end of weeks 1, 2, 3, 4, and 8. Body weight was recorded at screening, baseline and at the end of weeks 2, 3, 4, 6, and 8. Physical examinations were performed at screening and at the end of week 8. Blood and urine samples were obtained at the end of weeks 1, 2, 4, and 8 for laboratory analyses and pregnancy testing. Blood samples for pharmacokinetic analyses were taken at baseline, and at the end of weeks 1 and 2.

Concomitant medication

Any medication taken at the time of screening, with the exception of current antipsychotic treatment, was to be continued unaltered throughout the study, with medications maintained at the same doses used during the 4 weeks prior to randomization. Permitted medications included anticonvulsants used as "mood stabilizers", lithium, benzodiazepines (for anxiety, insomnia, or agitation), and anticholinergics (for symptoms of akathisia or EPS). Medications that were not permitted were known potent inhibitors of CYP2D6 or known inhibitors of or substrates for CYP3A4, and included SSRIs. Additional medications (i.e. those not used in a stable, ongoing manner during the 4 weeks before randomization) were allowed for the management of conditions (AEs or new intercurrent illness) that developed during the study.

Statistical procedures

Descriptive statistics were provided for most variables. Efficacy was summarized by tabulating the mean and standard deviation of the changes from baseline in the PANSS total, PANSS negative, PANSS positive, and CGI-S scores, and of the on-treatment CGI-I scores. Safety data for vital signs, body weight, laboratory, and ECG results were reviewed for potentially clinically significant events. The incidence of AEs was summarized by body system according to a modified COSTART dictionary. The change from baseline for Simpson-Angus Scale, Barnes Akathisia, and AIMS scores was summarized by means and standard deviations.

Results

Disposition of patients

A total of 311 predominantly male, chronic, stable outpatients with schizophrenia were randomized to treatment. Each of the three treatment groups was comparable for age, gender, race, weight, height, type of schizophrenia or schizoaffective disorder (Table 1). Pre-study antipsychotic medication use is shown in Table 2, and was comparable between the groups.

Overall, 224 of the 311 (72%) randomized patients completed the 8-week treatment period. The incidence of discontinuations was lower in group 3 (19%) than in group 1 (31%) or group 2 (34%). This difference was primarily due to between-group differences in the numbers of patients who withdrew consent (group 1: 7%, group 2: 12%, group 3: 1%; Table 3). There was no evidence that differences in discontinuation rates were due to clinically important differences in efficacy, safety or tolerability. The percentage of patients discontinuing due to worsening schizophrenia was comparable across all three treatment groups (10% in groups 1 and 2, 8% in group 3; P=0.751). The percentages of patients discontinuing due to adverse events other than worsening schizophrenia were: group 1, 6%, group 2: 10%, group 3: 6%.

Efficacy

For all three treatment groups, PANSS total, positive, and negative score assessments revealed changes from baseline directionally in favor of consistent, progressive improvement following the switch to aripiprazole (Table 4). The time course of change in PANSS total score is shown in Fig. 1; similarly, PANSS positive and PANSS negative scores also showed increasing improvement over the course of the study.

With the CGI-S and CGI-I scores, analysis of mean changes from baseline for both sets of ratings showed comparable improvement in scores following switch to

Table 1 Disposition and demographic characteristics of patients, including baseline efficacy ratings

		Treatment groups[a]			
		1	2	3	Total
Randomized		104	104	103	311
Gender [*n* (%)]	Male	68 (65)	72 (69)	78 (76)	218 (70)
	Female	36 (35)	32 (31)	25 (24)	93 (30)
Race [*n* (%)]	Caucasian	65 (62)	72 (69)	59 (57)	196 (63)
	Black	22 (21)	19 (18)	20 (19)	61 (20)
	Hispanic	8 (8)	5 (5)	10 (10)	23 (7)
	Asian	4 (4)	6 (6)	11 (11)	21 (7)
	Other	5 (5)	2 (2)	3 (3)	10 (3)
Age (years)	Mean (±SD)	39.7 (±10.3)	37.1 (±9.5)	39.3 (±10.9)	38.7 (±10.3)
Weight (kg)	Mean (±SD)	90.9 (±21.3)[b]	90.6 (±22.3)	89.1 (±19.6)	90.2 (±21.0)[c]
Diagnosis [*n* (%)]					
Schizophrenia type	Disorganized	3 (3)	0	4 (4)	7 (2)
	Paranoid	50 (48)	55 (53)	55 (53)	160 (51)
	Residual	2 (2)	3 (3)	1 (1)	6 (2)
	Undifferentiated	14 (14)	15 (14)	8 (8)	37 (12)
Schizoaffective disorder type	Bipolar	22 (21)	15 (14)	18 (18)	55 (18)
	Depressive	13 (13)	16 (15)	17 (17)	46 (15)
Baseline efficacy ratings					
Mean PANSS scores	Total	70.2[b]	68.0	70.9	69.7[c]
	Positive	16.3[b]	16.0	16.3	16.2[c]
	Negative	19.9[b]	18.2	20.4	19.5[c]
Mean CGI score	Severity of Illness	4.0[b]	3.9	4.0	4.0[c]

[a] Treatment group 1: immediate initiation of 30 mg oral aripiprazole with simultaneous discontinuation of current antipsychotic therapy; treatment group 2: immediate initiation of 30 mg oral aripiprazole while tapering off current antipsychotic therapy; treatment group 3: titrating oral aripiprazole upwards over 2 weeks while tapering off current antipsychotic therapy over the same 2 weeks

[b] n=103

[c] n=310

Table 2 Antipsychotic use at study entry

Antipsychotic use [*n* (%)]	Treatment groups[a]			
	1	2	3	Total
Typical	8 (8)	8 (8)	8 (8)	24 (8)
Haloperidol	6 (6)	3 (3)	6 (6)	15 (5)
Thioridazine	1 (1)	5 (5)	1 (1)	7 (2)
Other	1 (1)	1 (1)	0	2 (1)
Atypical	96 (92)	96 (92)	95 (92)	287 (92)
Olanzapine	57 (55)	58 (56)	58 (57)	173 (56)
Risperidone	38 (37)	37 (36)	37 (36)	112 (36)

[a] Treatment group 1: immediate initiation of 30 mg oral aripiprazole with simultaneous discontinuation of current antipsychotic therapy; treatment group 2: immediate initiation of 30 mg oral aripiprazole while tapering off current antipsychotic therapy; treatment group 3: titrating oral aripiprazole upwards over 2 weeks while tapering off current antipsychotic therapy over the same 2 weeks

Table 3 Discontinuation rates

	Number (%) of patients			
	Treatment groups[a]			
	1	2	3	Total
Randomized (*n*)	104	104	103	311
Completed [*n* (%)]	72 (69)	69 (66)	83 (81)	224 (72)
Discontinued	32 (31)	35 (34)	20 (19)	87 (28)
AEs (other than worsening schizophrenia)	6 (6)	10 (10)	6 (6)	22 (7)
Worsening schizophrenia	10 (10)	10 (10)	8 (8)	28 (9)
Non-compliance	4 (4)	1 (1)	1 (1)	6 (2)
Patients withdrew consent	7 (7)	12 (12)	1 (1)	20 (6)
Other[b]	5 (5)	2 (2)	4 (4)	11 (4)

[a] Treatment group 1: immediate initiation of 30 mg oral aripiprazole with simultaneous discontinuation of current antipsychotic therapy; treatment group 2: immediate initiation of 30 mg oral aripiprazole while tapering off current antipsychotic therapy; treatment group 3: titrating oral aripiprazole upwards over 2 weeks block;while tapering off current antipsychotic therapy over the same 2 weeks
[b] Lost to follow-up, protocol violation, patient met withdrawal criteria

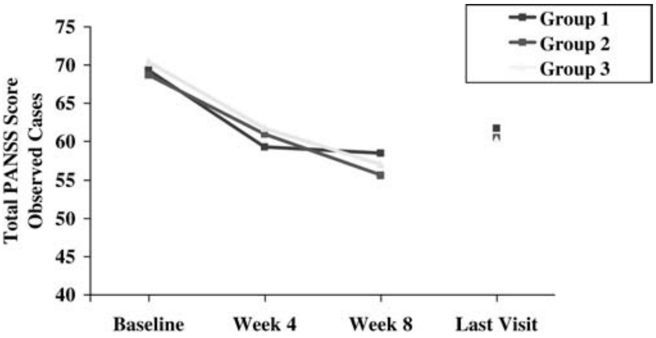

Fig. 1 Mean change in PANSS Total Score, Efficacy Sample. Group 1: immediate initiation of 30 mg oral aripiprazole with simultaneous discontinuation of current antipsychotic therapy. Group 2: immediate initiation of 30 mg oral aripiprazole while tapering off current antipsychotic therapy. Group 3: titrating oral aripiprazole upwards over 2 weeks while tapering off current antipsychotic therapy over the same 2 weeks

aripiprazole for the three treatment groups over the duration of the study. Improvements in these chronic and stable patients were consistently progressive from week 1 to endpoint. The changes from baseline to last visit for these ratings and the mean CGI-I scores are summarized in Table 4.

Safety and tolerability

Adverse events

The majority of patients in the safety data set reported at least one AEs during the study although most were mild to moderate in intensity. The incidence was broadly comparable in all three treatment groups with 89%, 89%, and 81% recorded in treatment groups 1, 2, and 3, respectively. Those AEs with a reported incidence of 10% or greater are summarized in Table 5. The most frequently reported AEs was insomnia, which was reported by 28%, 32% and 33% of patients in treatment groups 1, 2 and 3, respectively. The majority of insomnia (94%) cases were described as mild or moderate, and insomnia was cited as a reason for discontinuation by only two of 309 patients in the safety sample. In patients reporting insomnia during the first week of treatment, symptoms resolved continuously over time.

For certain AEs, some variation existed between treatment groups: the incidence of diarrhea was noticeably lower in group 1 (2%) compared to group 2 (10%) and group 3 (7%), while the incidence of upper respiratory infection was noticeably lower in group 2 (5%) compared to groups 1 (9%) and 3 (13%). The incidence

Table 4 Mean changes from baseline to last visit for PANSS and CGI-S efficacy assessments and mean CGI Improvement scores (LOCF analyses)

	Treatment groups[a]					
	1		2		3	
	n	Mean (±SD)	*n*	(±SD)	*n*	(±SD)
PANSS total	97	−7.59 (±19.23)	100	−8.18 (±17.23)	101	−10.11 (±15.74)
PANSS positive	97	−1.59 (±5.15)	100	−2.00 (±5.09)	101	−2.75 (±5.49)
PANSS negative	97	−2.88 (±5.62)	100	−2.37 (±6.13)	101	−2.81 (±5.53)
CGI Severity of Illness	101	−0.43 (±0.71)	102	−0.37 (±0.81)	102	−0.39 (±0.76)
C Global Improvement	101	3.17 (±1.30)	102	3.31 (±1.28)	102	3.16 (±1.25)

[a]Treatment group 1: immediate initiation of 30 mg oral aripiprazole with simultaneous treatment group 2: immediate initiation of 30 mg oral aripiprazole while tapering off current antipsychotic therapy; treatment group 3: titrating oral aripiprazole upwards over 2 weeks while tapering off current antipsychotic therapy over the same 2 weeks

Table 5 Incidence of AEs reported by 10% or more of patients in any treatment group

Reported AEs [*n* (%)]	Treatment groups[a]		
	1 *n*=103	2 *n*=104	3 *n*=102
Insomnia	29 (28)	33 (32)	34 (33)
Nausea	23 (22)	23 (22)	11 (11)
Akathisia	15 (15)	11 (11)	21 (21)
Anxiety	13 (13)	16 (15)	15 (15)
Psychosis	16 (16)	13 (13)	15 (15)
Headache	9 (9)	11 (11)	14 (14)
Upper respiratory infection	8 (8)	5 (5)	13 (13)
Somnolence	12 (12)	11 (11)	11 (11)
Lightheadedness	12 (12)	7 (7)	10 (10)
Vomiting	7 (7)	12 (12)	2 (2)
Agitation	8 (8)	8 (8)	11 (11)
Diarrhea	2 (2)	10 (10)	7 (7)

[a]Treatment group 1: immediate initiation of 30 mg oral aripiprazole with simultaneous discontinuation of current antipsychotic therapy; treatment group 2: immediate initiation of 30 mg oral aripiprazole while tapering off current antipsychotic therapy; treatment group 3: titrating oral aripiprazole upwards over 2 weeks while tapering off current antipsychotic therapy over the same 2 weeks

rates of reported nausea (group 1: 22%; group 2: 22%; group 3: 11%) and vomiting (group 1: 7%; group 2: 12%; group 3: 2%) were noticeably lower in treatment group 3. Nearly all reports of nausea and vomiting occurred during the first 14 days of treatment, and the duration of symptoms was less than 7 days in the majority of patients. Nausea was cited as a reason for discontinuation by one patient in group 1 and two patients in group 2; no patients in the study discontinued due to vomiting. The incidence of akathisia was higher in group 3 (group 1: 15%, group 2: 11%, group 3: 21%). Overall, the majority (96%) of akathisia was described as mild or moderate, and akathisia was cited as a reason for discontinuation for only 4 of 309 patients in the safety sample (1%). In patients reporting akathisia during week 1 of treatment, symptoms resolved continuously over time.

Seven percent of patients in the safety group (23/309) experienced a serious adverse event (SAEs) that required hospitalization. The incidence of SAEs was comparable across the three treatment groups and the majority of were associated with worsening of schizophrenia. Only one SAE (psychosis) was considered to be probably related to study medication by the investigator. Three additional SAEs were considered possibly related to study medication, while the remaining SAEs were considered unrelated to study medication.

Movement disorders

Mean changes from baseline evaluation in Simpson-Angus Scale scores showed no worsening or mild improvement of parkinsonism in all treatment groups throughout the duration of the study. In addition, Barnes Akathisia Scale scores were comparable across all three treatment groups, with mean changes from baseline at all time points revealing no deterioration or mild improvement in akathisia. Analysis of mean changes from baseline in the AIMS total score, based on the first seven items of the scale, confirmed no worsening or mild improvement of dyskinesia in all treatment groups throughout the study. The mean changes from baseline to last visit, based on last observation carried forward (LOCF) analyses, for each of these rating scales were small, but all were negative, indicating improvement (Fig. 2).

The only concomitant medication used for the potential treatment of EPS was benztropine. Overall, 13 patients (4%) received benztropine during the study: two patients in group 1 (2%); four patients in group 2 (4%); seven patients in group 3 (7%).

Body weight

Switch to aripiprazole treatment was associated with a modest weight loss. Mean changes in body weight were comparable for all three treatment groups at all assessments. The mean changes from baseline to last visit for groups 1, 2, and 3 were: −1.4, −1.7 and −1.3 kg, respectively (Fig. 3).

The percentage of patients who experienced clinically significant weight gain (defined as an increase of ≥7%

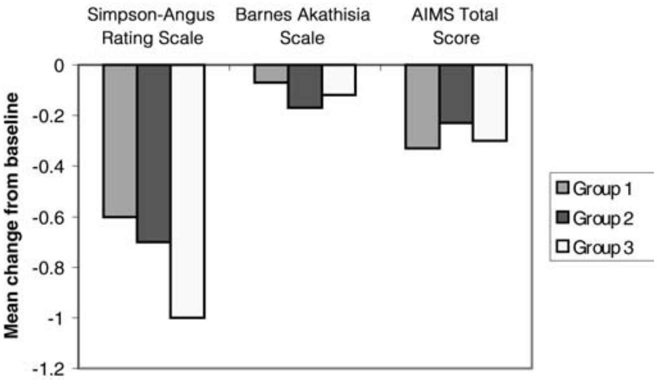

Fig. 2 Mean change from baseline to last visit for Simpson-Angus Scale, Barnes Akathisia Scale and AIMS total scores for all treatment groups. Group 1: immediate initiation of 30 mg oral aripiprazole with simultaneous discontinuation of current antipsychotic therapy. Group 2: immediate initiation of 30 mg oral aripiprazole while tapering off current antipsychotic therapy. Group 3: titrating oral aripiprazole upwards over 2 weeks while tapering off current antipsychotic therapy over the same 2 weeks

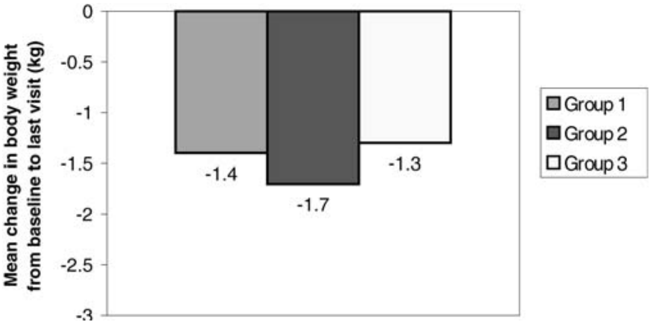

Fig. 3 Mean change in body weight from baseline to last visit for all treatment groups. Group 1: immediate initiation of 30 mg oral aripiprazole with simultaneous discontinuation of current antipsychotic therapy. Group 2: immediate initiation of 30 mg oral aripiprazole while tapering off current antipsychotic therapy. Group 3: titrating oral aripiprazole upwards over 2 weeks while tapering off current antipsychotic therapy over the same 2 weeks

from baseline) in groups 1, 2, and 3 were: 3%, 5%, and 3%, respectively. The incidence of clinically significant weight loss (defined as a decrease of ≥7% from baseline) in groups 1, 2 and 3 were: 7%, 15% and 8%, respectively.

Prolactin

Overall, aripiprazole produced reductions in serum prolactin concentrations. Mean serum prolactin levels at baseline were 22.5 ng/ml, 25.8 ng/ml, and 21.8 ng/ml in groups 1, 2, and 3, respectively (the normal range for serum prolactin levels defined by the central laboratory was 2–18 ng/ml in males and 2–23 ng/ml in non-lactating females). Mean changes from baseline to endpoint in groups 1, 2 and 3 were: −15.9, −19.4 and −16.2 ng/ml, respectively. No patients in any treatment group experienced prolactin levels below the lower limit of normal.

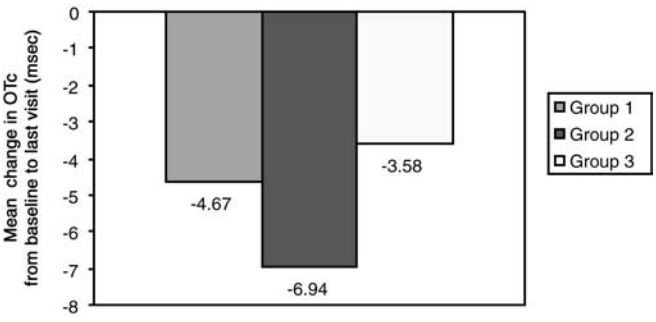

Fig. 4 Mean changes in QT_C interval from baseline to last visit for all treatment groups. Group 1: immediate initiation of 30 mg oral aripiprazole with simultaneous discontinuation of current antipsychotic therapy. Group 2: immediate initiation of 30 mg oral aripiprazole while tapering off current antipsychotic therapy. Group 3: titrating oral aripiprazole upwards over 2 weeks while tapering off current antipsychotic therapy over the same 2 weeks

Thirteen patients (group 1, *n*=1; group 2, *n*=4; group 3, *n*=8) experienced potentially clinically significant elevations in prolactin levels, defined as a serum prolactin level greater than the upper limit of normal. Three of these patients experienced prolactin elevations after discontinuing aripiprazole, and eight had prolactin elevations while still taking their prior antipsychotic (and had reduced values while on aripiprazole alone). Of the two patients who had elevated prolactin levels while on aripiprazole alone, one had an elevated prolactin level at baseline and had a lower prolactin level while on aripiprazole alone, and the other had a subsequent normal prolactin level while on aripiprazole alone.

QT_c interval

QT_c intervals were calculated using the FDA Neuropharmacology Division's correction factor ($QT_{cN}=QT/RR^{0.37}$). Mean changes in QT_C interval were −4.67 ms, −6.94 ms, and −3.58 ms in groups 1, 2 and 3, respectively (Fig. 4).

No patients in any of the treatment groups had a potentially clinically significant increase in QT_c interval (defined as ≥450 ms and ≥10% increase from baseline).

Vital signs and laboratory abnormalities

There were no clinically significant differences in vital sign or laboratory values between the treatment groups, and no patients discontinued the study due to vital sign or laboratory abnormalities.

There was no evidence of any clinically relevant abnormalities during physical examinations. One pregnancy occurred during the study and this patient subsequently had a normal delivery.

Discussion

This study showed that all three strategies were equally effective for switching patients with chronic and stable schizophrenia or schizoaffective disorder to aripiprazole treatment from previous antipsychotic monotherapy. Aripiprazole was well tolerated by patients using all three strategies: immediate initiation of aripiprazole with immediate discontinuation of current antipsychotic therapy (group 1); immediate initiation of aripiprazole while tapering off current antipsychotic therapy (group 2); up-titration of aripiprazole while tapering off current antipsychotic medication (group 3). In addition, efficacy ratings showed small improvements from baseline during the study in all three groups.

Overall, 72% of patients (224/311) completed the 8-week study. Completion rates were similar for groups 1 and 2 (69% and 66%, respectively), but significantly higher in group 3 (81%; P=0.035). The difference in rates between the groups was, however, almost entirely due to administrative reasons. Thus differences in completion rates between the groups do not appear to reflect differences in the tolerability of the different switching strategies.

Efficacy was maintained among chronic and stable patients throughout the study in all three treatment groups. Efficacy assessments (PANSS and CGI Scale scores) showed no evidence of a deterioration in schizophrenia symptoms from baseline in any treatment group. Changes from baseline in PANSS total score, PANSS positive and PANSS negative assessments confirmed that switching treatment to aripiprazole enabled chronic and stable patients to maintain or somewhat improve upon the level of control of positive and negative symptoms they had attained with prior therapy. Consistent and progressive improvements in efficacy ratings were observed throughout the study in each of the treatment groups; it is, however, possible that the fact that this study was open rather than blinded may have contributed to the efficacy improvements and the low relapse rates.

The overall safety profile was similar for all three treatment groups. Aripiprazole treatment was well tolerated in all three groups, with the majority of AEs being mild to moderate in intensity. The overall incidence of AEs was generally comparable between the groups, with notable differences only observed for nausea and vomiting, which were less frequent in group 3 than in groups 1 and 2. The majority of reported nausea or vomiting occurred during the first 14 days of treatment and lasted for less than 1 week. Analysis of the movement disorder rating scales (Simpson-Angus Scale, Barnes Akathisia Scale, and AIMS) showed small improvements in mean scores for all three scales during the study.

Weight gain with antipsychotics has been the subject of increasing attention over the past several years, and is linked to adverse cardiovascular and metabolic outcomes, as well as decreased compliance with treatment (Goodnick 2001; McIntyre 2001; Meltzer 2001; Meyer 2001; Sussman 2001). After switching to aripiprazole, patients lost between 1.3 and 1.7 kg over the 8-week study period.

This study demonstrates that switching patients from prior antipsychotic therapy to aripiprazole can be successfully accomplished using any one of three switching strategies. While gradual cross titration may be a preferred option for some patients, all three switching strategies with aripiprazole showed favorable efficacy, safety and tolerability results. Previous placebo-controlled clinical trials have demonstrated that aripiprazole is effective against both positive and negative symptoms of schizophrenia and has an excellent safety and tolerability profile (Carson et al 2001; Yeung et al 2001). Consistent with previous studies, the current study demonstrated no evidence of increased liability for EPS, weight gain, somnolence, increased prolactin levels, or QT_c prolongation. Given that the patients in the current study have chronic, stable disease, improvements in positive and negative symptoms upon switching to a new medication would not necessarily be expected. However, aripiprazole demonstrated improvements in both positive and negative symptoms in all three treatment groups, possibly because of aripiprazole's unique mechanism of action. Additional benefits included improvements in EPS, weight loss, and prolactin normalization, in the absence of QT_c prolongation. For chronic, stable patients who have failed treatment with other agents due to inadequate response or lack of tolerability, switching to aripiprazole can be achieved with favorable results using any of the three strategies evaluated, allowing physicians flexibility to choose the strategy with which they are most comfortable and which is best for their patients.

Acknowledgements This study was supported by Bristol-Myers Squibb and Otsuka Pharmaceutical Co.

Members of the Aripiprazole Study Group: S. Altinsan, Pharmacology Research Corporation, Reno, Nev., USA; T.M. Antin, PACT Atlanta LLC, Decatur, Ga., USA; M. Banov, Northwest Behavioral Research Center, Marietta, Ga., USA; B. Bastani, North Coast Clinical Trials, Beachwood, Ohio, USA; R. Bielski, Institute for Health Studies, Okemos, Mich., USA; J. Carr, Affiliated Research Institute, Claremont, Calif., USA; S. Cheren, Stanley Cheren, MD, Inc., Brookline, Mass., USA; A. Cutler, Coordinated Research of Florida, Inc., Winter Park, Fla., USA; J.M. Ferguson, Pharmacology Research Clinic, Salt Lake City, Utah, USA; D.L. Fogelson, Pacific Psychopharmacology Research, Santa Monica, Calif., USA; A.M. Freeman, III, Louisiana State University Medical Center, Shreveport, La., USA; H.L. Goldberg, Kauai Community Mental Health Center, Lihue, Hawaii, USA; M.S. Hale, New Britain General Hospital, New Britain, Conn., USA; M.B Hamner, Ralph H. Johnson VA Medical Center, Charleston, S.C., USA; J.T. Hartford, Hartford Research Group, Cincinnati, Ohio, USA; I.S. Kolin, Irving S. Kolin, MD, PA, Winter Park, Fla., USA; G.L. Larson, VA Medical Center, Milwaukee, Wisc., USA; D. Mee-Lee, Hawaii Clinical Research Center, Honolulu, Hawaii, USA; A.L. Miller, University Health Center-Downtown, San Antonio, Tex., USA; J. Miller, Clinical Neuroscience Solutions, West Palm Beach, Fla., USA; J.J. Pahl, Pahl Brain Associates, Inc., Oklahoma City, Okla., USA; R.L. Pearlman, Sisters of Charity Medical Center, Staten Island, N.Y., USA; D.A. Sack, The Institute of Psychopharmacology, Cerritos, Calif., USA; J.S. Simon, Northbrooke Research Center, Brown Deer, Wisc., USA; K. Sokolski, Affiliated Research Institute, Santa Ana, Calif., USA; V. Spratlin, Mercer University, Atlanta, Ga., USA; T.K. Tran-Johnson, California Neuropsychopharmacology, San Diego, Calif., USA; M.M. Viner, Pharmacology Research Corporation, Reno, Nev., USA; S.A. West, Psychiatric Institute of Florida, PA,

Orlando, Fla., USA; C. Wronski, Affiliated Research Institute, Santa Ana, Calif., USA; D.L. Zimbroff, Pacific Clinical Research, San Bernardino, Calif., USA.

References

Barnes TRE (1989) A rating scale for drug-induced akathisia. Br J Psychiatry 154:672–676

Bunney WE, Meltzer HY (1995) Schizophrenia—overview. Clin Neurosci 3:55–56

Burris KD, Molski TF, Ryan E, Xu C, Tottori K, Kikuchi T, Yocca FD, Molinoff PB (2000) Aripiprazole is a high affinity partial agonist at human D_2 dopamine receptors. Int J Psychopharmacol 3:S129

Carson WH, McQuade RD, Ali M, Saha AR, Dunbar GC, Ingenito GA (2001) A double-blind, placebo-controlled trial of aripiprazole and haloperidol. Schizophrenia Res (Special Issue VIIIth International Congress on Schizophrenia Research, Whistler, British Columbia, Canada) 49:221

Carson WH, Stock E, Saha AR, Ali M, McQuade RD, Kujawa MJ, Ingenito G (2002) Meta-analysis of safety and tolerability with aripiprazole. Abstracts of the XIth Biennial Winter Workshop on Schizophrenia, Davos, Switzerland, February 24–March 1 2002. Schizophr Res 53:1–274

Casey DE (1997) The relationship of pharmacology to side effects. J Clin Psychiatry 58:55–62

Davis JM, Schaffer CB, Killian GA, Kinard C, Chan C (1980) Important issues in the drug treatment of schizophrenia. Schizophr Bull 6:70–87

Dossenbach MRK, Beuzen JN, Avnon M, Belmaker RH, Elizur A, Mark M, Munitz H, Schneidman M, Shoshani D, Kratky P, Grundy SL, Tollefson GD (2000) The effectiveness of olanzapine in treatment-refractory schizophrenia when patients are nonresponsive to or unable to tolerate clozapine. Clin Ther 22:1021–1034

Dossenbach MR, Kratky P, Schneidman M, Grundy SL, Metcalfe S, Tollefson GD, Belmaker RH (2001) Evidence for the effectiveness of olanzapine among patients nonresponsive and/or intolerant to risperidone. J Clin Psychiatry 6:28–34

Fleischhacker WW, Meise U, Gunther V, Kurz M (1994) Compliance with antipsychotic drug treatment: influence of side effects. Acta Psychiatr Scand Suppl 382:11–15

Glennon RA, Dukat M (1995) Serotonin receptor subtypes. In: Bloom FE, Kupfer DJ (eds) Psychopharmacology. The fourth generation of progress. Raven Press, New York, pp 415–430

Goodnick PJ, Kato MM (2001) Antipsychotic medication: effects on regulation of glucose and lipids. Exp Opin Pharmacother 2:1571–1582

Guy W (1976a) Abnormal involuntary movement scale (AIMS). National Institute of Mental Health. In: ECDEU Assessment Manual for Psychopharmacology, Revised 1976. National Institute of Mental Health, Psychopharmacology Research Branch, Division of Basic Brain and Behavioral Sciences, Rockville, Md.

Guy W (1976b) Clinical Global Impressions (CGI). In: ECDEU Assessment Manual for Psychopharmacology. US Department of Health and Human Services, Public Health Service, Alcohol Drug Abuse and Mental Health Administration, NIMH Psychopharmacology Research Branch, Rockville, Md., pp. 218–222

Inoue T, Domae M, Yamada K, Furukawa T (1996) Effects of the novel antipsychotic agent 7-(4-[4-(2,3-dichlorophenyl)-1-piperazinyl]butyloxy)-3,4-dihydro-2(1H)-quinolinone (OPC-14597) on prolactin release from the rat anterior pituitary gland. J Pharmacol Exp Ther 277:137–143

Jan MW (1988) Buspirone: an update on a unique anxiolytic agent. Pharmacotherapy 8:100–116

Jordan S, Koprivica V, Chen R, Tottori K, Kikuchi T, Altar CA (2001) The antipsychotic aripiprazole is a potent, partial agonist at the human $5\text{-}HT_{1A}$ receptor. Eur Neuropsychopharmacol 11:S268

Kane JM (1987) Treatment of schizophrenia. Schizophr Bull 13:133–156

Kane JM, Carson WH, Saha AR, McQuade RD, Ingenito GG, Zimbroff DL, Ali MW (2002) Efficacy and safety of aripiprazole and haloperidol versus placebo in patients with schizophrenia and schizoaffective disorder. J Clin Psychiatry 63:763–771

Kay SR, Opler LA, Fiszbein A (1987) The Positive and Negative Syndrome Scale (PANSS) for schizophrenia. Schizophr Bull 13:261–276

Kikuchi T, Tottori K, Uwahodo Y, Hirose T, Miwa T, Oshiro Y, Morita S (1995) 7-{4-[4-(2,3-Dichlorophenyl)-1-piperazinyl]butoxyl}-3,4-dihydro-2(1*H*)-quinolone (OPC-14597), a new putative antipsychotic drug with both presynaptic and postsynaptic D_2 receptor antagonist activity. J Pharmacol Exp Ther 274:329–336

Kinon BJ, Kane JM, Johns C et al. (1993) Treatment of neuroleptic resistant schizophrenia relapse. Psychopharmacol Bull 29:309–314

Kinon BJ, Basson BR, Gilmore, JA, Malcolm S, Stauffer VL (2000) Strategies for switching from conventional antipsychotic drugs or risperidone to olanzapine. J Clin Psychiatry 61:833–840

Kolakowska T, Williams AO, Arden M et al. (1985) Schizophrenia with good and poor outcome. I: Early clinical features, response to neuroleptics and signs of organic dysfunction. Br J Psychiatry 146:229–239

Kurzthaler I, Fleishhacker WW (2001) The clinical implications of weight gain in schizophrenia. J Clin Psychiatry 62:32–37

Leysen JE, Janssen PMF, Schotte A, Luyten WHML, Mergens AAHP (1993) Interaction of antipsychotic drugs with neurotransmitter receptor sites in vitro and in vivo in relation to pharmacological and clinical role of $5\text{-}HT_2$ receptors. Psychopharmacology 112:S40

Lieberman JA, Johns CA, Kane JM, Rai K, Pisciotta AV, Saltz BL and Howard A (1988) Clozapine-induced agranulocytosis: non-cross-reactivity with other psychotropic drugs. J Clin Psychiatry 49:271–277

Littrell KH, Johnson CG, Hilligoss NM, Peabody CD, Littrell SH (2000) Switching clozapine responders to olanzapine. J Clin Psychiatry 61:912–915

McIntyre RS, Mancini DA, Basile VS (2001) Mechanisms of antipsychotic-induced weight gain. J Clin Psychiatry 62:23–29

Meltzer HY (2001) Putting metabolic side effects into perspective: risks versus benefits of atypical antipsychotics. J Clin Psychiatry 62:35–39

Meyer JM (2001) Effects of atypical antipsychotics on weight and serum lipid levels. J Clin Psychiatry 62:27–34

Millan MJ (2000) Improving the treatment of schizophrenia: focus on serotonin $(5\text{-}HT)_{1A}$ receptors. J Pharmacol Exp Ther 295:853–861

Peuskens J (2000) Switching approach in the management of schizophrenia patients. Int Clin Psychopharmacol 15:S15–19

Remington G, Chong S-A (1999) Conventional versus novel antipsychotics: changing concepts and clinical implications. J Psychiatr Neurosci 24:431–441

Saha AR, Carson WH, Ali MW, Dunbar GC, Ingenito G (2001) Efficacy and safety of aripiprazole and risperidone vs placebo in patients with schizophrenia and schizoaffective disorder. W J Biol Psychiatry 2:305S

Simpson GM, Angus JSW (1970) A rating scale for extrapyramidal side effects. Acta Psychiatr Scand 45:11–19

Sovner R, Parner-Sovner N (1989) Use of buspirone in the treatment of schizophrenia. J Clin Psychopharmacol 9:61–61

Sussman N (2001) Review of atypical antipsychotics and weight gain. J Clin Psychiatry 62:5–12

Yeung PP, Carson WH, Saha AR, McQuade RD, Ali MW, Stringfellow JC, Ingenito G (2001) Efficacy of aripiprazole, a novel antipsychotic, in schizophrenia and schizoaffective disorder: results of a placebo-controlled trial with risperidone. Eur Neuropsychopharmacol 11:S241
