# Supplementary material for: Dose-Dependent Efficacy of Aripiprazole in Treating Patients With Schizophrenia or Schizoaffective Disorder: A Systematic Review and Meta-Analysis of Randomized Controlled Trials
Source: Front Psychiatry. 2021 Aug 11;12:717715. doi: 10.3389/fpsyt.2021.717715 (PMC8385236; doi:10.3389/fpsyt.2021.717715)
Supplement: Supplementary file 1 [file Data_Sheet_1.ZIP › supplementray material-9 RCTs/8 Cheng 2018.pdf]

·专题报道·

不同剂量阿立哌唑治疗慢性精神分裂症的效果对比

程国强，钟健荣，鲁金韵，邱国亮
肇庆市第三人民医院，广东 肇庆 526060

摘要：目的 探讨不同剂量阿立哌唑治疗慢性精神分裂症的效果差异，以期寻找一个疗效与安全性最佳状态的治疗方案。**方法** 选取肇庆市第三人民医院2014年1月—2016年1月收治的78例慢性精神分裂症患者，采用随机数字方法将其分为观察组和对照组，每组39例。其中观察组给予30 mg/d阿立哌唑治疗，对照组给予20 mg/d阿立哌唑治疗，两组均治疗6个月，采用阴性与阳性综合征量表（PANSS）和生活质量量表（QOL）评价两组治疗前后疗效及两组不良反应的发生情况。**结果** 两组患者治疗前QOL量表和PANSS量表评分比较差异无统计学意义（$P>0.05$），治疗后观察组QOL量表评分明显高于对照组（$P<0.05$），观察组PANSS量表评分明显低于对照组（$P<0.05$）；观察组和对照组治疗期间不良反应发生率分别为12.82%和10.26%，差异无统计学意义（$P>0.05$）。**结论** 使用30 mg/d阿立哌唑治疗慢性精神分裂症患者疗效更加显著，对于稳定病情和防止复发均有积极作用，安全性好。

关键词： 慢性精神分裂症；阿立哌唑；剂量

中图分类号： R473.74 **文献标志码：** A **文章编号：** 1674-8166(2018)07-0972-02

DOI:10.16440/j.cnki.1674-8166.2018.07.049

精神分裂症患者中发部分处于病情迁延的慢性状态，合并有阴性症状和阳性症状，临床上使用传统的抗精神病药物的治疗效果较不理想，阿立哌唑是新一类的抗精神病药物，对于改善患者的阴性症状及阳性症状均有较为显著的效果[1,2]。目前国内关于阿立哌唑用药剂量对此类疾病的患者治疗效果方面的相关研究较少，本研究重点对不同剂量的阿立哌唑治疗慢性精神分裂症的疗效，现报告如下。

1 资料与方法

1.1 一般资料 选取肇庆市第三人民医院2014年1月—2016年1月收治的88例慢性精神分裂症患者作为研究对象，采用随机数字方法分为观察组和对照组，每组39例。其中观察组男27例，女12例；年龄23～69岁，平均年龄（43.5±4.1）岁；病程1～13年，平均病程（5.8±0.6）年。对照组男26例，女13例；年龄22～69岁，平均年龄（42.4±3.9）岁；病程1～14年，平均病程（5.7±0.7）年。两组患一般资料比较差异无统计学意义（$P>0.05$），具有可比性。本研究经过肇庆市第三人民医院伦理委员会批准，所有患者知情同意并签署知情同意书。

1.2 治疗方法 观察组采用30 mg/d的阿立哌唑（国药准字H20041501，成都康弘药业集团股份有限公司）治疗，对照组剂量为20 mg/d治疗，3个月为1个疗程，两组均治疗2个疗程。

1.3 评价方法 采用生活质量量表（QOL）[3]对两组患者生活质量进行评价，包括12项统计指标，每项1～5分，评分越高则患者的生活质量越好；采用PANSS量表[4]对两组患者的病情进行评估，包括阴性症状和阳性症状各7项，评分越高则症状越重。比较两组治疗期间不良反应的发生情况。

1.4 统计学处理 采用SPSS20.0统计软件进行统计分析，计量资料用均数±标准差（$\bar{x}\pm s$）表示，采用t检验，计数资料用率（%）表示，采用χ^2检验，$P<0.05$为差异有统计学意义。

2 结果

2.1 两组治疗效果比较 两组患者治疗前QOL量表和PANSS量表评分比较差异无统计学意义（$P>0.05$），治疗后观察组QOL量表评分明显高于对照组，差异有统计学意义（$P<0.05$），观察组PANSS量表评分明显低于对照组，差异有统计学意义（$P<0.05$）。见表1。

2.2 两组患者治疗期间不良反应发生情况比较 两组患者治疗期间不良反应发生率比较，差异无统计学意义（$P>0.05$）。见表2。

作者简介： 程国强，大学本科，主治医师，研究方向：精神卫生

通信作者： 程国强，E-mail：aptangs@126.com

?1994-2019 China Academic Journal Electronic Publishing House. All rights reserved. http://www.cnki.net

表 1　两组治疗前后 QOL 量表和 PANSS 量表评分比较（$\bar{x}\pm s$）

组别	例数	QOL 评分	PANSS 总分
观察组	39		
治疗前		39.55±12.31	92.83±9.16
治疗后		53.53±13.44[ab]	52.27±5.88[ab]
对照组	39		
治疗前		38.32±11.58	92.76±9.40
治疗后		41.32±8.53[a]	77.31±6.42[a]

注：与本组治疗前比较，a 为 $P<0.05$；与对照组治疗后比较，b 为 $P<0.05$

表 2　两组患者治疗期间不良反应发生情况比较[例（%）]

组别	例数	口干	头晕	视物模糊	便秘	总发生
观察组	39	1(2.56)	1(2.56)	1(2.56)	2(5.13)	5(12.82)
对照组	39	1(2.56)	2(5.13)	0(0.00)	1(2.56)	4(10.26)

注：组间比较，$\chi^2=0.126$，$P=0.723$

3 讨论

慢性精神分裂症患者多数合并阴性和阳性症状，采用传统的抗精神病药物的疗效并不理想，且该种药品造成的锥体外系反应较为常见。精神分裂症患者的阳性症状可能和大脑中边缘巴胺功能处于亢进状态具有较为密切的关系，则患者表现出的阴性症状与 5-HT 系统关系密切。阿立哌唑对 5-羟色胺受体与多巴胺 D_2 受体具有一定的激动活性[5]。阿立哌唑属于最新一种类型的非典型抗精神病药物，同时也是二氢喹啉酮药物，该药物的药理作用机制以及化学结构和其他的抗精神病药物具有一定的差异性，故为第三代抗精神病药物[6]。慢性精神分裂症患者认知功能障碍的严重程度会随着病程延长以及抗精神病类药物治疗效果起到缓慢进程的作用。在治疗慢性精神分裂症患者时，药物剂量的严格控制具有重要的作用[7,8]。因此，本研究对不同剂量阿立哌唑疗效进行比较，结果显示，两组患者治疗前 QOL 量表和 PANSS 量表评分差异无统计学意义（$P>0.05$），治疗后观察组 QOL 量表评分明显高于对照组（$P<0.05$），观察组 PANSS 量表评分明显低于对照组（$P<0.05$），其原因可能在于在合理用药基础上，阿立哌唑的剂量越高，则其发挥受体阻断作用越强，对于患者认知功能的改善以及生活质量的改善均较为明显[9]。提示了经过一段时间治疗，患者的生活质量也有了联动提高。另外本研究结果显示，两组患者治疗过程中发生的不良反应包括口干、头晕、视物模糊、便秘，但两组不良反应发生率比较差异无统计学意义（$P>0.05$），表明 30 mg/d 的阿立哌唑尚处于安全范围内，具有较高的安全性，不会加重患者的不良反应发生情况[10]。孟彬等[11]研究显示，阿立哌唑一方面可以改善精神分裂症患者的各种症状，另一方面对患者的血浆催乳素水平不会产生明显的干扰，甚至能够对已经出现的高催乳素血症以及闭经等产生逆转效果，也进一步提示对于出现闭经的女性患者可选用该种药物。需要强调的是，阿立哌唑的剂量处于合理范围是一个重要的前提，且在实际使用过程中应注重考虑患者的自身情况，因此应综合考虑患者的病情决定用药剂量。

综上所述，使用 30 mg/d 阿立哌唑治疗慢性精神分裂症患者疗效更加显著，对于稳定病情和防止复发均有积极作用。

参考文献：

[1] 张展星，褚庆文，陆怡．氨磺必利联合奥氮平改善精神分裂症患者认知功能的疗效观察[J]．现代药物与临床，2016，31(6):897-900.
[2] 唐娟，路孝琴，陈丽芬，等．北京市城市社区精神分裂症患者及家庭对社会资源知晓和利用情况及其影响因素调查分析[J]．中国全科医学，2016，19(20):2459-2463.
[3] 孙妍，郭建兵，李贺斌，等．社会支持对精神分裂症患者生活质量和康复效果的影响[J]．解放军医药杂志，2017，29(5):72-74.
[4] 白晓英，刘建武，杨睿，等．精神分裂症患者劳动能力鉴定 PANSS 量表应用分析[J]．陕西医学杂志，2009，38(4):508-509.
[5] 汪卫华，刘丽，李光耀，等．阿立哌唑治疗奥氮平所致精神分裂症患者体质量增加的随机双盲对照研究[J]．临床精神医学杂志，2016，26(2):89-91.
[6] 赵明军，毛亚阁，张瑞岭．低剂量阿立哌唑增效治疗难治性抑郁症的有效性和安全性的 Meta 分析[J]．临床药物治疗杂志，2017，15(2):43-48.
[7] 张艳，康传媛，元静，等．COMT 基因多态性与利培酮治疗精神分裂症临床疗效及认知功能的关联性分析[J]．中华行为医学与脑科学杂志，2017，26(1):32-36.
[8] 王慧，周娱菁，刘靖，等．青少年精神分裂症患者的健康相关危险行为[J]．中国心理卫生杂志，2016，30(5):363-368.
[9] 唐磊，罗加国．棕榈酸帕利哌酮与利培酮治疗精神分裂症患者有效性与安全性的开放、随机、对照研究[J]．临床精神医学杂志，2016，26(2):98-101.
[10] 王潇，张燕，孙梦夕，等．青少年精神分裂症首次发病患者脑灰质体积与认知功能的相关性[J]．临床精神医学杂志，2016，26(6):365-367.
[11] 孟彬，张炳奎，武景霞．阿立哌唑治疗闭经的女性精神分裂症患者[J]．上海精神医学，2010，22(3):168.

收稿日期：2018-01-05　**修回日期：**2018-02-14　**责任编辑：**戴旭旭

?1994-2019 China Academic Journal Electronic Publishing House. All rights reserved.　http://www.cnki.net
