# Supplementary material for: Dose-Dependent Efficacy of Aripiprazole in Treating Patients With Schizophrenia or Schizoaffective Disorder: A Systematic Review and Meta-Analysis of Randomized Controlled Trials
Source: Front Psychiatry. 2021 Aug 11;12:717715. doi: 10.3389/fpsyt.2021.717715 (PMC8385236; doi:10.3389/fpsyt.2021.717715)
Supplement: Supplementary file 1 [file Data_Sheet_1.ZIP › supplementray material-9 RCTs/6 Xu 2016.pdf]

实用医药杂志,2013,3(12):79－80

[9] 何佳,徐东勋. 脑梗死高危人群抑郁有关危险因素研究[J]. 精神医学杂志,2015,28(3):211－213

[10] 刘宗和,田旭,张静. 文拉法辛合并奥氮平治疗老年抑郁症的对照研究[J]. 精神医学杂志,2014,27(3):218－219

[11] 许二平. 中风后抑郁症的研究进展[J]. 河南中医,2007,27(1):75－78

[12] 赵靖平. 精神病学新进展[M]. 北京:中华医学电子音像出版社, 2009: 149－151

[13] 梁颂游. 心理干预对老年脑卒中患者神经功能缺损和幸福感的影响[J]. 精神医学杂志,2014,27(3):215－217

[14] 赵丽莎,毛佩贤,鲍枫,等. 社区老年慢性病患者生活质量及影响因素分析[J]. 精神医学杂志,2014,27(1):16－19

[15] 崔爱军,郑玉英,石永存,等. 高频重复经颅磁刺激合并艾司西酞普兰治疗老年难治性抑郁症对照研究[J]. 精神医学杂志,2014,27(4):300－302

[16] 蒋重蓉,耿姝霞. 草酸艾司西酞普兰治疗老年期抑郁症随期对照研究[J]. 西部医学, 2011, 23(11): 2107－2108

[17] 白树新, 王德斌, 李广玉, 等. 草酸艾司西酞普兰与盐酸帕罗西汀治疗脑卒中后抑郁的临床对照研究[J]. 河北医药, 2013, 35(2): 230－231

(收稿日期:2015－11－09)
(修回日期:2015－12－09)

阿立哌唑差异性用法对慢性精神分裂症的疗效分析

徐炳聪

【摘要】 目的　探讨阿立哌唑差异性用法对慢性精分症患者的疗效。**方法**　将 124 例慢性精神分裂症患者根据随机数字表法随机分成 30 mg/d 阿立哌唑组(30 mg/d 组)及 20 mg/d 阿立哌唑组(20 mg/d 组)各 62 例,使用不同剂量阿立哌唑治疗 6 个月。于治疗前及治疗后采用阳性和阴性综合征量表(PANSS)及生活质量量表(QOL)评定疗效,使用治疗中需处理的不良反应症状量表(TESS)评定药物不良反应。**结果**　30 mg/d 组总有效率高于 20 mg/d 组($P<0.05$)。治疗后两组 PANSS 总分及各因子分均较各自治疗前降低($P<0.05$),两组 QOL 评分均较各自治疗前升高($P<0.05$)。治疗后 30 mg/d 组 PANSS 总分及各因子分均低于 20 mg/d 组($P<0.05$);30 mg/d 组 QOL 评分高于 20 mg/d 组($P<0.05$)。两组总不良反应发生率比较差异无统计学意义($P>0.05$)。治疗前 QOL 评分与 PANSS 总分无相关性($P>0.05$),治疗后 QOL 评分与 PANSS 总分呈负相关($P<0.05$)。**结论**　治疗期间应用 30 mg/d 的阿立哌唑对于慢性精神分裂症患者的病情具有较好的改善作用,且能更好稳定病情,防止复发,值得临床推荐。

【关键词】　阿立哌唑　慢性精神分裂症　临床疗效　安全性
【中图分类号】R749.3　**【文献标识码】**　A　**【文章编号】**　2095－9346(2016)－02－0131－03
doi:10.3969/j.issn.2095－9346.2016.02.014

慢性精神分裂症在精神科占有很大比例,其治疗及康复已经成为世界上研究的重点。非典型抗精神病药物的使用在精神科对慢性精神分裂症的治疗及康复中已取得一定的成效,但进展仍然较慢。在临床工作中,应用阿立哌唑对慢性精神分裂症患者的治疗较为普遍[1]。然而,Kishi T 等[2]和陆德青等[3]报道指出,在治疗期间使用不同剂量的阿立哌唑对于慢性精神分裂症患者的病情改善作用亦有所差异。鉴于国内较少涉及阿立哌唑用药剂量对慢性精神分裂症治疗的报道,本研究旨在分析慢性精神分裂症患者在治疗期间使用不同剂量的阿立哌唑对其病情疗效及安全性的影响,目的在于寻找兼具疗效与安全性的用药方案,现报道分析如下。

1　对象与方法

1.1　对象　选取 2013 年 5 月～2015 年 2 月在广州市民政局精神病院接受治疗的慢性精神分裂症患者 124 例进行研究。纳入标准:(1)符合世界卫生组织(WHO)编写的国际疾病分类第 10 版(ICD-10)中精神分裂症的诊断标准[4],病情至少持续两年;(2)性别不限;(3)年龄 26～66 岁;(4)阳性和阴性综合征量表(PANSS)总分≥60 分;(5)入组前 2 周内未使用任何抗精神病药物;(6)均已告知患者及其家属本研究的目的、方法及意义,自愿参加本研究并签署知情同意书者,并可配合接受本次研究。排除标准:(1)有其他严

作者单位:510430 广东广州,广州市民政局精神病院

重的心、肝、肾等功能性障碍；(2) 处于妊娠或者哺乳期妇女；(3) 有癫痫或者药物依赖者；(4) 对阿立哌唑过敏者；(5) 无法配合完成研究者。根据随机数字表法将入组患者随机分成 30 mg/d 阿立哌唑组(30 mg/d 组)及 20 mg/d 阿立哌唑组(20 mg/d 组)各 62 例。其中 30 mg/d 组男 30 例，女 32 例；年龄 26～63 岁，平均年龄(42.6±3.8)岁；病程 1～16 年，平均病程(5.9±0.4)年。20 mg/d 组男 28 例，女 34 例；年龄 28～66 岁，平均年龄(42.3±2.2)岁；病程 2～18 年，平均病程(5.6±0.2)年。两组患者性别、年龄以及病程等资料比较差异无统计学意义($P>0.05$)，具有可比性。

1.2　方法

1.2.1　治疗方法　30 mg/d 组阿立哌唑用药剂量为 30 mg/d，20 mg/d 组阿立哌唑用药剂量为 20 mg/d，两组均治疗 2 个疗程，每个疗程 3 个月，分别在治疗前及治疗 2 个疗程后进行临床疗效及安全性对比。

1.2.2　量表评定　分别于治疗前及治疗后采用 PANSS 量表及生活质量量表(QOL)评定疗效[5]。其中 QOL 量表由 12 项统计指标构成，每项 1～5 分，评分越高表示患者的生活质量越好。患者的病情评估使用 PANSS 判定，此量表包含阳性症状量表、阴性症状量表各 7 项，以及一般病理量表计 16 项，评分越高表示患者的症状越严重。利用 PANSS 减分率评价疗效，减分率＝[(治疗前评分－治疗后评分)/(治疗前评分－30)]×100%，其中 PANSS 减分率≥75% 为痊愈，50%～74% 为显著进步，25%～49% 为进步，<25% 为无效。使用治疗中需处理的不良反应症状量表(TESS)[6]评定药物不良反应。

1.2.3　统计学方法　采用 SPSS13.0 统计软件分析，数据比较采用 χ^2 检验，计量数据以 $\bar{x}\pm s$ 表示，予以 t 检验。$P<0.05$ 为差异有统计学意义。

2　结果

2.1　两组临床疗效比较　治疗后，30mg/d 组痊愈 9 例，显著进步 12 例，进步 32 例，无效 9 例，总有效率 85.48%；20 mg/d 组痊愈 7 例，显著进步 8 例，进步 28 例，无效 19 例，总有效率 69.53%。30 mg/d 组总有效率高于 20 mg/d 组($\chi^2=4.613$, $P=0.032$)。

2.2　两组治疗前后 PANSS、QOL 评分比较　治疗前，两组 PANSS 总分及各因子分、QOL 评分比较，差异无统计学意义($P>0.05$)。治疗后两组 PANSS 总分及各因子分均较各自治疗前降低($P<0.05$)，两组 QOL 评分均较各自治疗前升高($P<0.05$)。治疗后 30 mg/d组 PANSS 总分及各因子分均低于 20 mg/d 组($P<0.05$)；30 mg/d 组 QOL 评分高于 20 mg/d 组($P<0.05$)。见表 1。

表 1　两组治疗前后量表评分比较($\bar{x}\pm s$)

量表	20mg/d 组($n=62$)	30mg/d 组($n=62$)	t 值	P 值
阳性症状				
治疗前	21.31±4.68	21.64±3.92	0.426	0.671
治疗后	16.94±3.22*	12.88±2.31*	2.106	0.037
阴性症状				
治疗前	23.31±6.42	23.26±5.87	0.045	0.964
治疗后	16.89±1.82*	12.21±1.05*	2.548	0.012
一般病理症状				
治疗前	45.16±7.57	44.99±6.83	0.131	0.896
治疗后	37.99±2.12*	27.24±2.08*	1.988	0.041
PANSS 总分				
治疗前	93.72±10.66	93.58±11.31	0.071	0.944
治疗后	73.96±2.13*	53.20±2.14*	1.982	0.049
QOL 评分				
治疗前	38.64±10.23	38.72±9.81	0.044	0.965
治疗后	48.17±2.49*	52.33±2.68*	8.954	0.000

注：与治疗前比较，* $P<0.05$

2.3　两组不良反应比较　治疗后两组出现的不良反应为头晕、口干、视物不清、便秘、体质量增加，各不良反应发生率比较差异均无统计学意义($P>0.05$)。见表 2。20 mg/d 组总不良反应发生率为 4.84%，30 mg/d组总不良反应发生率为9.68%，两组总不良反应发生率比较，差异无统计学意义($\chi^2=1.078$, $P=0.299$)。

表 2　两组不良反应比较[n(%)]

不良反应	20mg/d 组($n=62$)	30mg/d 组($n=62$)	χ^2 值	P 值
头晕	1(1.61)	2(3.23)	0.342	0.559
口干	1(1.61)	2(3.23)	0.342	0.559
视物不清	0(0)	1(1.61)	–	0.314*
便秘	1(1.61)	0(0)	–	0.314*
体质量增加	0(0)	1(1.61)	–	0.314*

注：* 采用 fisher 精确概率法

2.4　两组 QOL 评分与 PANSS 总分的相关性分析　治疗前 QOL 评分与 PANSS 总分无相关性($r=0.104$, $P=0.225$)，治疗后 QOL 评分与 PANSS 总分呈负相关($r=-0.846$, $P<0.05$)。

3　讨论

临床上，慢性精神分裂症患者在精神科很常见，近年来的报道研究亦显示[7~9]，认知功能障碍在精神分裂症的患者群体中较为常见，且此种障碍程度可随着患者病程的延长及抗精神病类药物的治疗表现出缓慢进展特征。因此，在对慢性精神分裂症患者进行治疗时，药物剂量应严格予以控制。鉴于此，本文通过对比慢性精神分裂症患者在治疗期间应用不同剂量的阿立哌唑治疗后产生的临床疗效，旨在寻找更加合理的阿立哌唑用药剂量。

本研究发现,两组患者在治疗前 PANSS、QOL 评分比较差异无统计学意义($P>0.05$)。提示应用不用剂量的阿立哌唑前,两组慢性精神分裂症患者的病情基本处于同一水平。但治疗后 30 mg/d 组的 PANSS 总分及各因子分均低于 20 mg/d 组($P<0.05$),且治疗后 30 mg/d 组的 QOL 评分高于 20 mg/d 组($P<0.05$),表明 30 mg/d 组的阿立哌唑用药剂量对患者病情的改善程度更佳,且对患者生活质量的改善作用更好。原因可能在于在合理的药物剂量上,阿立哌唑的药物含量水平越高,则其发挥的受体相关阻断作用亦更加明显,对患者认知功能的改善情况随之越好,也能够更好地改善患者的生活质量。而治疗前 QOL 评分与 PANSS 总分无相关性,治疗后 QOL 评分与 PANSS 总分呈负相关。这提示经过一段时间的阿立哌唑药物治疗后,患者生活质量亦得到了相应的联动提升。

同时,本研究还发现,30 mg/d 组的总有效率是 85.48%,高于 20 mg/d 组的 69.53%($P<0.05$),这提示对处于维持治疗期间的慢性精神分裂症患者实施阿立哌唑药物治疗所得疗效明显更好。这亦符合国外 Srisurapanont M 等[10]报道的在 30 mg/d 剂量的维持用药剂量下,慢性精神分裂症患者的恢复情况较好这一结论。究其原因,这可能是因为阿立哌唑是新型非典型性抗精神病药物,其亦为二氢喹啉酮类药物。其化学结构式及相应的药理作用机制与其他类的抗精神病药物有所差异,具有革新性,因此又被称为第三代的抗精神病药物。在作用机制方面,其不仅能够阻断患者机体的 5-HT_{2A} 受体,同时还可阻断其突触前膜中 5-HT_{1A} 受体的有关作用,进而提升多巴胺能,并部分激动对应的 D_1 及 D_2 等多巴胺受体,从而在较大程度上改善患者的认知障碍,最终增大药物治疗疗效[11]。需要说明的是,维持治疗时合理的阿立哌唑药物剂量是一个基础性前提,且在实际用药的过程中应结合患者自身恢复情况合理使用药物,30 mg/d 仅为同等条件下的参考剂量值,而并非固定剂量值,因此,临床医师在治疗时应综合考虑患者的病情。

此外,本文研究还显示,两组患者总不良反应发生率比较差异无统计学意义($P>0.05$)。表明应用 30 mg/d 剂量的阿立哌唑并不会增加患者的不良反应,具有较高的安全性。原因可能在于无论是 20 mg/d 亦或是 30 mg/d 均在阿立哌唑推荐用药剂量的安全范围内,因此不会发生太大差异的药物不良反应。这亦与国外 Raoufinia A 等[12]报道的结果基本一致。

综上所述,维持治疗期间应用 30 mg/d 的阿立哌唑对于慢性精神分裂症患者的病情具有较好的改善作用,防止复发,对于其病情康复具有重要的意义,值得临床推荐。

参考文献

[1] 张峰,王江.阿立哌唑治疗精神分裂症后抑郁的临床疗效及患者生活质量分析[J].中华神经医学杂志,2013,12(5):521-524

[2] Kishi T, Matsuda Y, Matsunaga S, et al. Aripiprazole for the management of schizophrenia in the Japanese population: a systematic review and meta-analysis of randomized controlled trials [J] Neuropsychiatr Dis Treat, 2015, 20(11):419-434

[3] 陆德青,肖刚,刘春华,等.齐拉西酮与阿立哌唑治疗女性首发精神分裂症的随机对照研究[J].中华临床医师杂志(电子版),2013,1(14):6688-6689

[4] 世界卫生组织.ICD-10 精神与行为障碍分类:临床描述与诊断要点[M].北京:人民卫生出版社,1993:225

[5] 张作记.行为医学量表手册[J].中国行为医学科学,2001,10(特刊):143-147

[6] 张明园.精神科评定量表手册[M].2 版.长沙:湖南科学出版社,2003:150-153,197-202

[7] 李祥.阿立哌唑增效治疗女性难治性精神分裂症的研究[J].中华全科医学,2013,11(10):1573-1574

[8] Zhang S, Lan G. Prospective 8-week trial on the effect of olanzapine, quetiapine, and aripiprazole on blood glucose and lipids among individuals with first-onset schizophrenia [J]. Shanghai Arch Psychiatry,2014,26(6):339-346

[9] 范汉同.阿立哌唑治疗脑血管病所致精神障碍的对照研究[J].中华老年心脑血管病杂志, 2011,13(1):5-7

[10] Srisurapanont M, Suttajit S, Maneeton N, et al. Efficacy and safety of aripiprazole augmentation of clozapine in schizophrenia: A systematic review and meta-analysis of randomized-controlled trials [J]. J Psychiatr Res,2015, 1(62):38-47

[11] 岳英,林治光,马玉苹,等.喹硫平、阿立哌唑对精神分裂症患者血小板 5-羟色胺水平的影响[J].中华行为医学与脑科学杂志,2010,19(1):36-38

[12] Raoufinia A, Baker RA, Eramo A, et al. Initiation of aripiprazole once-monthly in patients with schizophrenia [J]. Curr Med Res Opin,2015,31(3):583-592

(收稿日期:2015-11-24)
(修回日期:2015-12-14)
