# Supplementary material for: Dose-Dependent Efficacy of Aripiprazole in Treating Patients With Schizophrenia or Schizoaffective Disorder: A Systematic Review and Meta-Analysis of Randomized Controlled Trials
Source: Front Psychiatry. 2021 Aug 11;12:717715. doi: 10.3389/fpsyt.2021.717715 (PMC8385236; doi:10.3389/fpsyt.2021.717715)
Supplement: Supplementary file 1 [file Data_Sheet_1.ZIP › supplementray material-9 RCTs/3 Potkin 2003.pdf]

ORIGINAL ARTICLE

Aripiprazole, an Antipsychotic With a Novel Mechanism of Action, and Risperidone vs Placebo in Patients With Schizophrenia and Schizoaffective Disorder

Steven G. Potkin, MD; Anutosh R. Saha, PhD; Mary J. Kujawa, MD, PhD; William H. Carson, MD; Mirza Ali, PhD; Elyse Stock, MD; Joseph Stringfellow, MS; Gary Ingenito, MD, PhD; Stephen R. Marder, MD

Background: Aripiprazole is a dopamine D_2 receptor partial agonist with partial agonist activity at serotonin $5HT_{1A}$ receptors and antagonist activity at $5HT_{2A}$ receptors. This multicenter trial examined the efficacy, safety, and tolerability of aripiprazole in patients with acute exacerbation of schizophrenia or schizoaffective disorder.

Methods: In this 4-week double-blind study, 404 patients were randomized to 20 mg/d (n=101) or 30 mg/d (n=101) of aripiprazole, placebo (n=103), or 6 mg/d of risperidone (n=99). Efficacy assessments included Positive and Negative Syndrome Scale (PANSS) scores and Clinical Global Impression scores. Safety and tolerability evaluations included extrapyramidal symptoms and effects on weight, prolactin, and corrected QT (QT_c) interval.

Results: Aripiprazole (20 and 30 mg/d) and risperidone (6 mg/d) were significantly better than placebo on all efficacy measures. Separation from placebo occurred at week 1 for PANSS total and positive scores with aripiprazole and risperidone and for PANSS negative scores with aripiprazole. There were no significant differences between aripiprazole and placebo in mean change from baseline in the extrapyramidal symptom rating scales. Mean prolactin levels decreased with aripiprazole but significantly increased 5-fold with risperidone. Mean change in QT_c interval did not differ significantly from placebo with any active treatment group. Aripiprazole and risperidone groups showed a similar low incidence of clinically significant weight gain.

Conclusions: Aripiprazole is effective, safe, and well tolerated for the positive and negative symptoms in schizophrenia and schizoaffective disorder. It is the first non-D_2 receptor antagonist with clear antipsychotic effects and represents a novel treatment development for psychotic disorders.

Arch Gen Psychiatry. 2003;60:681-690

THE INTRODUCTION of the second-generation or atypical antipsychotics was a major advance in the pharmacotherapy of schizophrenia. These agents are associated with a substantially lower liability for extrapyramidal adverse effects and a reduced risk of tardive dyskinesia compared with first generation or typical agents. The improved tolerability profile of atypical antipsychotics has been attributed to their serotonin $5HT_{2A,C}$ receptor antagonism and/or $5HT_{1A}$ partial agonism in addition to their dopamine D_2 receptor antagonism.[1-3] Others have emphasized the potential importance of the disassociation rate (K_{off}) of these antagonists at the dopamine receptor.[4-6] Although atypical agents have demonstrated efficacy against positive and negative symptoms compared with placebo, they are associated with a range of other adverse effects including hyperprolactinemia (which may produce sexual adverse effects, gynecomastia, and galactorrhea), weight gain, increased risk for diabetes mellitus, and prolongation of the corrected QT (QT_c) interval on electrocardiograms (ECGs). These adverse effects are associated with potential long-term health risks as well as decreased adherence to treatment regimens.[7,8]

All currently available antipsychotic agents, both conventional and atypical, are dopamine D_2 receptor antagonists. Blockade of dopamine receptors in the mesolimbic pathway is thought to mediate antipsychotic efficacy, in particular the ability to decrease positive symptoms.[9,10] However, D_2 receptor blockade in the mesocortical, nigrostriatal, and tuberoinfundibular pathways is correlated with a dysfunctional reward system and increased liability for extrapyramidal symptoms (EPS) and hyperprolactinemia—unwanted side effects of antipsychotic therapy.[11]

The development of D_2 partial agonists is a logical strategy for the treatment of schizophrenia because the patho-

Author affiliations are listed at the end of this article.

©2003 American Medical Association. All rights reserved.
Downloaded From: http://archpsyc.jamanetwork.com/ by a University of St. Andrews Library User on 05/24/2015

physiologic mechanism of schizophrenia is thought to be based on too much dopamine activity in some regions of the brain and too little dopamine activity in other regions. A D_2 partial agonist can act as a functional antagonist in areas of high levels of dopamine, such as the mesolimbic pathway, but not in areas of normal dopamine levels, such as the nigrostriatal and tuberoinfundibular pathways. Thus, a D_2 partial agonist is expected to reduce the positive symptoms of schizophrenia without producing movement disorders or elevated prolactin levels. In regions of low dopamine concentration, such as the mesocortical pathway, a D_2 partial agonist will show functional agonist activity. Initial studies with a partial dopamine agonist SDZ HDC 912 indicated the potential of this approach.[12] However, although showing efficacy comparable to that of haloperidol in a 4-week double-blind randomized trial, this compound did not provide the expected advantages over conventional antipsychotics because the reduction in negative symptoms and the incidence of EPS were also comparable between the 2 treatment arms. Another partial dopamine agonist, terguride, was examined in open-label pilot trials, which indicated efficacy against negative symptoms only.[13,14] Preclamol, (−)3-PPP or (−)-3-(3-hydroxyphenyl)-*N*-n-propylpiperidine, another partial dopamine agonist, showed significant improvement in both the positive and negative symptoms of psychosis after 7 days of therapy, but the antipsychotic actions were not sustained at later points.[15] Differences in clinical effects of these dopamine partial agonists may be due to their differential intrinsic activity at D_2 receptors.[16]

Aripiprazole shows potent partial agonist activity at cloned human D_2 receptors in vitro.[17] In these experiments, aripiprazole exhibited agonist properties qualitatively similar to those of dopamine but different quantitatively. In the absence of dopamine, aripiprazole activates D_2 receptors, resulting in a net increase of receptor activity; this activity is less than that elicited by dopamine. If dopamine is present, aripiprazole inhibits its binding to D_2 receptors, and receptor activity is decreased; because of the intrinsic activity of aripiprazole, the activation of D_2 receptors is not fully abolished.[17] In ex vivo experiments, aripiprazole inhibited spontaneous prolactin release from the isolated anterior pituitary slices.[18] This inhibition was less than that achieved with the full dopamine D_2 receptor agonist talipexole and could be completely blocked by haloperidol. In vivo, aripiprazole displays D_2 antagonist effects in animal models of dopaminergic hyperactivity (eg, blockade of apomorphine hydrochloride–induced stereotypy) and D_2 agonist activity in a model of dopaminergic hypoactivity (inhibition of increased dopamine synthesis in reserpine-treated rats).[19]

Additional preclinical studies have indicated that aripiprazole shows relatively high affinity for serotonin $5HT_{2A}$ and $5HT_{1A}$ receptors. At $5HT_{1A}$ receptors, aripiprazole displayed partial agonist activity.[20] Partial agonist action at $5HT_{1A}$ receptors has been hypothesized to correlate with overall efficacy against the symptoms of schizophrenia, including anxiety, depression, and cognitive and negative symptoms.[2] Aripiprazole has also been shown to have antagonist activity at $5HT_{2A}$ receptors[21] similar to that of other atypical agents. Antagonist activity at $5HT_{2A}$ receptors is thought to be associated with a low liability for EPS[1] and beneficial effects on the negative symptoms of schizophrenia.[22,23]

The aim of the present study was to investigate the efficacy, safety, and tolerability of 20 mg and 30 mg doses of aripiprazole for the treatment of acute psychosis in patients with schizophrenia and schizoaffective disorders, including evaluation of negative symptoms and the relationship of aripiprazole doses with time to response. Risperidone, a widely available atypical antipsychotic, was used as the active control in this study to measure the study group's response to treatment. The study was not designed to detect a difference in response between the active treatment groups.

METHODS

The study was conducted in accordance with Good Clinical Practice procedures, Food and Drug Administration regulations, and the Declaration of Helsinki. Approval was gained from the institutional review board or ethics committee at each of the study medical centers. All patients gave informed, written consent, which was cosigned by their next of kin or caregiver if required by the local institutional review board.

INCLUSION AND EXCLUSION CRITERIA

Men and nonpregnant, nonlactating women aged 18 to 65 years with a primary diagnosis of schizophrenia or schizoaffective disorder (*DSM-IV*) and who were hospitalized due to an acute relapse were eligible for enrollment in the study. For inclusion, patients had to have evidence for responsiveness to antipsychotic medication (ie, were not refractory to antipsychotics, had previously shown an improvement with an antipsychotic drug other than clozapine, and had been an outpatient for at least one 3-month period during the past year), a Positive and Negative Syndrome Scale (PANSS) total score of at least 60, and a minimum score of 4 (moderate) on at least 2 items of the psychotic item subscale. Patients taking a long-acting neuroleptic could be included if a time period of at least 1 treatment cycle plus 1 week had elapsed since their last treatment or if a shorter time period had elapsed but they were judged to be clinically deteriorating by the investigator.

Study exclusion criteria included a psychiatric disorder other than schizophrenia or schizoaffective disorder requiring pharmacotherapy; a history of violence; a recent history of suicidal attempts or serious suicidal ideation; a clinically significant neurological abnormality other than tardive dyskinesia or EPS; current diagnosis of psychoactive substance dependence or a history of drug or alcohol abuse (*DSM-IV*) within 1 month of the start of the study; or treatment with an investigational drug within 4 weeks before the washout phase. Patients were also to be excluded if they had any other acute or unstable medical condition.

STUDY DESIGN

This was a randomized, 4-week, inpatient, double-blind, placebo-controlled, parallel-group study, conducted at 40 medical centers in the United States between September 1997 and October 1998. Patients meeting all inclusion criteria and none of the exclusion criteria underwent a minimum 5-day placebo washout period starting within 1 week of the screening visit. Patients completing the washout period were evaluated for eligibility for inclusion in the treatment phase of the study and were excluded if they were diagnosed with a psychiatric disorder other than

©2003 American Medical Association. All rights reserved.
Downloaded From: http://archpsyc.jamanetwork.com/ by a University of St. Andrews Library User on 05/24/2015

schizophrenia or schizoaffective disorder during the washout period, had a clinically significant abnormal laboratory value, or had any acute or unstable medical condition.

Patients were randomized to receive 1 of 4 treatments for 4 weeks: 20 mg/d of aripiprazole, 30 mg/d of aripiprazole, 6 mg/d of risperidone, or placebo. The risperidone dosing regimen was selected based on the package insert of the drug and clinical practice at the time the study was initiated. Risperidone dosages were titrated upward (2 mg on day 1, 4 mg on day 2, and 6 mg/d for the remainder of the study) and administered orally after breakfast and after the evening meal. Aripiprazole was given as a fixed full dose orally after breakfast, with placebo given in the evening to maintain study blinding. The placebo group received placebo in the morning and the evening. Dosages were fixed throughout the study and could not be increased for lack of efficacy or decreased for the occurrence of adverse events. Patients were hospitalized for the entire duration of the study.

EFFICACY EVALUATIONS

Treatment efficacy was assessed using the PANSS and Clinical Global Impression (CGI) Scale. The PANSS evaluation included the total score (30 items), the positive subscale (7 items), and the negative subscale (7 items). The severity of each symptom on these subscales was rated on a 7-point scale. The CGI consisted of two 7-point subscales: the Severity of Illness (CGI-S) and Global Improvement (CGI-I) scales. For each patient, the same rater conducted the assessment throughout the study and was blinded to the patient's treatment.

Efficacy evaluations were performed at screening, at the end of the washout period (baseline), and at the end of each week of treatment (days 7, 14, 21, and 28). The primary efficacy parameters were the change from baseline in PANSS total score, PANSS positive score, and CGI-S score. Secondary efficacy parameters included the change from baseline in PANSS negative score. Other assessments included the number and percentage of responders (patients with a ≥30% decrease from baseline in PANSS total score or a score of 1 [very much improved] or 2 [much improved] on the CGI-I scale), change from baseline in PANSS-derived Brief Psychiatric Rating Scale (BPRS) core score, and mean CGI-I scores.

SAFETY EVALUATIONS

Adverse events were monitored at baseline and thereafter weekly by asking patients if they had experienced any problems or symptoms since the previous week. Investigators graded the intensity of events (mild, moderate, or severe) and assessed their likely relationship to the study medication. The status and intensity of previously reported events was also evaluated at each weekly assessment.

The occurrence of parkinsonism, akathisia, and dyskinesia was evaluated using standardized EPS rating scales: the Simpson-Angus Scale (SAS), the Barnes Akathisia Rating Scale (BAS), and the Abnormal Involuntary Movements Scale (AIMS), respectively. The SAS and AIMS each assess 10 items using a 5-point scale of severity (SAS: 1, normal, to 5, severe; AIMS: 0, none, to 4, severe) and give a mean overall score for each patient. The BAS comprises 3 items assessing akathisia and an overall global clinical assessment.

Vital signs (pulse and systolic and diastolic blood pressure) were measured at screening, at baseline, and on days 1 through 5, 14, and 28 of the study. Body weight and serum prolactin levels were measured at baseline and on days 14 and 28. Twelve-lead ECGs and hematological parameters, serum chemical parameters, and urinalysis results were obtained at screening, baseline (not ECG), and on days 14 and 28.

CONCOMITANT MEDICATION

The use of psychotropic drugs other than those prescribed by the study protocol was prohibited during the study, with the exception of lorazepam for anxiety or insomnia or intramuscular lorazepam for emerging agitation if deemed necessary by the investigator. Benztropine mesylate treatment for EPS was permitted during the double-blind treatment phase up to a maximum dosage of 6 mg/d, if judged necessary by the investigator. All concomitant medications used during the study were recorded on the appropriate case report forms.

STATISTICAL PROCEDURES

Primary efficacy analyses were based on data obtained at a patient's last visit, regardless of whether they had completed the study, ie, last observation carried forward analysis at week 4. The primary treatment comparisons were 20 mg of aripiprazole vs placebo and 30 mg of aripiprazole vs placebo. Continuous efficacy data (eg, change from baseline) were evaluated by analysis of covariance, adjusting for baseline values and study medical center. Categorical efficacy data (eg, CGI-I scores and responders) were analyzed using the Cochran-Mantel-Haenszel test, controlling for study medical center.

The primary efficacy treatment comparisons were tested by following a stepdown procedure, ie, first 30 mg of aripiprazole vs placebo was tested at a 2-tailed significance level of .05; if rejected, 20 mg of aripiprazole vs placebo was tested at a 2-tailed significance level of .05. A monotonic dose-response was assumed.

For safety parameters, mean changes from baseline were evaluated without adjustment for baseline values, study center, or other variables. The percentage of patients with significant weight gain was evaluated by the Cochran-Mantel-Haenszel test as described above.

RESULTS

PATIENT DEMOGRAPHICS AND DISPOSITION

A total of 404 patients were randomized to 4 treatment groups for 4 weeks (**Figure 1**): 20 mg/d of aripiprazole (n=101), 30 mg/d of aripiprazole (n=101), 6 mg/d of risperidone (n=99), or placebo (n=103). Overall, 283 patients (70%) were male, and the mean age for patients in each treatment group ranged from 38.1 to 40.2 years. Of the total patient population, 289 (72%) were diagnosed with schizophrenia, and 115 (28%) were diagnosed with schizoaffective disorder. At baseline, the range in mean PANSS total score for the treatment groups was 92.6 to 95.7. Baseline demographic parameters were well matched across the 4 groups in the study (**Table 1**). Baseline body weight ranged from 82.4 to 87.2 kg. The mean number of prior hospitalizations was 8.6.

Of the 404 patients randomized to treatment, 403 were included in the safety analysis. The efficacy analysis included 392 patients after a further 11 patients were excluded because they did not have a postrandomization efficacy evaluation (9 withdrew consent for personal reasons, and 2 were lost to follow-up).

In all, 242 patients (60%) completed the 4-week study period, and 162 discontinued treatment. Forty-two (10%) of 404 patients discontinued due to lack of

©2003 American Medical Association. All rights reserved.
Downloaded From: http://archpsyc.jamanetwork.com/ by a University of St. Andrews Library User on 05/24/2015

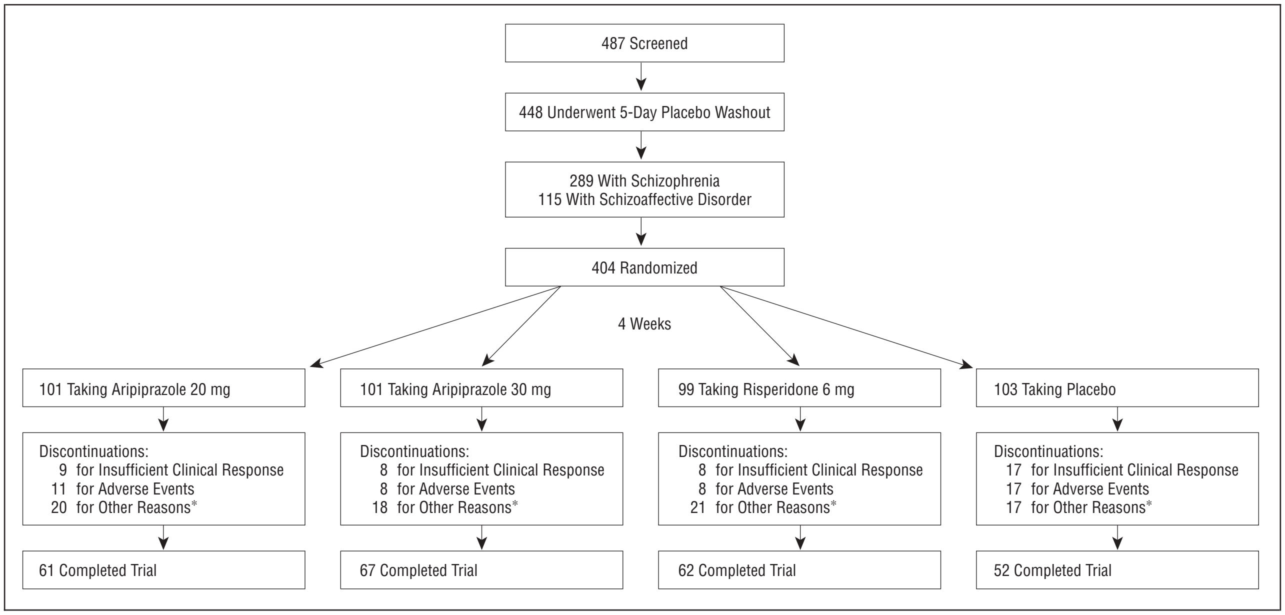

Figure 1. Summary of participant flow in the randomized clinical trial. Asterisk indicates consent withdrawal, protocol violation, noncompliance, and loss to follow-up.

Table 1. Baseline Demographic Characteristics, Including Efficacy and Safety Parameters at Baseline

Characteristic	Placebo (n = 103)	Aripiprazole 20 mg (n = 101)	Aripiprazole 30 mg (n = 101)	Risperidone 6 mg (n = 99)	Total (N = 404)
Male/female, No.	73/30	73/28	66/35	71/28	283/121
Mean age, y	38.8	38.1	40.2	38.6	38.9
Mean body weight, kg	85.2	87.2	84.0	82.4	84.7
Mean serum prolactin level, ng/mL*	10.7	12.1	12.7	9.0	11.1
Mean No. of hospitalizations	7.0	8.1	7.7	11.8	8.6
Mean PANSS score					
Total	95.7	94.4	92.6	94.9	94.4
Positive	24.8	24.7	24.1	24.2	24.4
Negative	23.6	23.6	23.2	24.8	23.8
Mean CGI-S score	4.8	4.8	4.8	4.8	4.8

Abbreviations: CGI-S, Clinical Global Impression Severity of Illness Scale; PANSS, Positive and Negative Syndrome Scale.
*Data are missing for some patients: placebo, n = 95; aripiprazole 20 and 30 mg, n = 94 and 87, respectively; risperidone 6 mg, n = 91; and total, n = 367.

clinical response or worsening schizophrenia, 50 (12%) discontinued due to personal reasons, and 44 (11%) discontinued due to adverse events. The numbers of discontinuations were highest in the placebo group (n=51; 50%) and similar in the 3 active treatment groups (aripiprazole 20 mg, n=40 [40%]; aripiprazole 30 mg, n=34 [34%]; risperidone, n=37 [37%]) (**Table 2**).

EFFICACY DATA

Primary Efficacy Measures

Both doses of aripiprazole produced significant improvements in the 3 primary efficacy parameters compared with placebo: PANSS total score (aripiprazole 20 mg, $P=.001$; aripiprazole 30 mg, $P=.003$), PANSS positive score (aripiprazole 20 mg, $P=.001$; aripiprazole 30 mg, $P=.02$), and CGI-S score (aripiprazole 20 mg, $P=.03$; aripiprazole 30 mg, $P=.006$). The risperidone group also showed significantly greater improvement on all primary efficacy measures, confirming the responsiveness of the patient population to active treatment (**Table 3**). All active treatments demonstrated rapid onset of efficacy, with statistically significant effects evident from week 1 for both PANSS total scores and PANSS positive scores (**Figure 2**A and B).

Secondary and Other Efficacy Measures

Both aripiprazole dosages produced significantly greater improvements compared with placebo for CGI-I (aripiprazole 20 mg, $P=.005$; aripiprazole 30 mg, $P=.001$), PANSS negative (aripiprazole 20 mg, $P=.002$; aripiprazole 30 mg, $P=.002$) (Figure 2C), and PANSS-derived BPRS core scores (aripiprazole 20 mg, $P=.004$; aripiprazole 30 mg, $P=.01$) (Table 3). For all 3 parameters, the statistically significant differences between both aripiprazole dosages and placebo were evident from week 1 onward. Statistically significant differences between risperidone and placebo were evident from week 2 for PANSS

©2003 American Medical Association. All rights reserved.
Downloaded From: http://archpsyc.jamanetwork.com/ by a University of St. Andrews Library User on 05/24/2015

Table 2. Discontinuation Rates*

	Placebo (n = 103)	Aripiprazole 20 mg (n = 101)	Aripiprazole 30 mg (n = 101)	Risperidone 6 mg (n = 99)	Total (N = 404)
Completed study	52 (50)	61 (60)	67 (66)	62 (63)	242 (60)
Discontinued	51 (50)	40 (40)	34 (34)	37 (37)	162 (40)
Reasons for discontinuation					
Adverse event	17 (17)	11 (11)	8 (8)	8 (8)	44 (11)
Noncompliance	0 (0)	1 (1)	2 (2)	1 (1)	4 (1)
Insufficient clinical response	17 (17)	9 (9)	8 (8)	8 (8)	42 (10)
Other†	17 (17)	19 (19)	16 (16)	20 (20)	72 (18)

*Values are given as the number (percentage) of patients.
†Includes patients who withdrew consent (owing to personal reasons and lack of effect), protocol violations, patients who met withdrawal criteria (1 patient in the aripiprazole 30-mg group received the wrong dose), and patients lost to follow-up.

Table 3. Efficacy Results: Last Observation Carried Forward Data

Parameter	Placebo (n = 103)	Aripiprazole 20 mg (n = 98)	*P* Value vs Placebo	Aripiprazole 30 mg (n = 96)	*P* Value vs Placebo	Risperidone 6 mg (n = 95)	*P* Value vs Placebo
PANSS score*							
Total	−5.0	−14.5	.001	−13.9	.003	−15.7	<.001
Positive	−1.8	−4.9	.001	−3.9	.02	−5.2	<.001
Negative	−0.8	−3.4	.002	−3.4	.002	−3.1	.005
PANSS-derived* BPRS core score	−1.7	−3.5	.004	−3.3	.01	−3.9	<.001
CGI-S score*	−0.2	−0.5	.03	−0.6	.006	−0.7	<.001
Mean CGI-I score	4.0	3.4	.005	3.3	.001	3.3	<.001
Responder rate, No. (%)†	24 (23)	35 (36)	.04	39 (41)	.005	38 (40)	.008

Abbreviations: BPRS, Brief Psychiatric Rating Scale; CGI-I, Clinical Global Impression Global Improvement Scale; CGI-S, Clinical Global Impression Severity of Illness Scale; PANSS, Positive and Negative Syndrome Scale.
*Values are given as the mean change from baseline.
†Patients with a 30% or more decrease from the baseline PANSS total score or a score of 1 (very much improved) or 2 (much improved) on the CGI-I scale.

negative score (Figure 2C) and from week 1 for CGI-I and PANSS-derived BPRS core scores.

Responder rates, based on changes in PANSS total and CGI-I scores from baseline to the last visit (as described in the "Methods" section), were statistically significantly superior for both aripiprazole dosages compared with placebo (placebo, 23%; aripiprazole 20 mg, 36%; $P=.04$; aripiprazole 30 mg, 41%; $P=.005$). Risperidone was also associated with a significantly higher responder rate than was placebo (40%; $P=.008$) (Table 3).

SAFETY

Adverse Events

Overall, both dosages of aripiprazole were well tolerated, with most adverse events being mild to moderate in intensity and generally not treatment-limiting. A total of 44 (11%) of 403 patients in the safety sample discontinued from the study due to adverse events: 17 patients (17%) in the placebo group, 8 patients (8%) in the risperidone group, 11 patients (11%) in the aripiprazole 20-mg group, and 8 patients (8%) in the aripiprazole 30-mg group. The most frequent adverse event that led to discontinuation was psychosis: 8 patients (8%) in the placebo group, 5 patients (5%) in the risperidone group, 10 patients (10%) in the aripiprazole 20-mg group, and 5 patients (5%) in the aripiprazole 30-mg group. The vast majority of these reports were deemed to be unrelated to study medication.

The number of subjects with treatment-related adverse events was similar for all 4 treatment groups: 91% (92/101) in the aripiprazole 20-mg group, 91% (91/100) in the aripiprazole 30-mg group, 93% (92/99) in the risperidone group, and 86% (89/103) in the placebo group. Adverse events occurring at an incidence of 5% or more in at least 1 treatment group are shown in **Table 4**. In patients receiving aripiprazole, adverse events of headache, nausea, vomiting, insomnia, and somnolence occurred mainly during the first week of treatment and generally did not exceed 1 week in duration. A dose-response relationship was not apparent for most adverse events, with the possible exception of somnolence.

Overall, 8 patients had a serious adverse event during the study (aripiprazole 20 mg, n=2; aripiprazole 30 mg, n=3; placebo, n=3). There were 4 reports of psychosis, and 1 report each of hernia, attempted suicide, cellulitis, and agitation. All these events were considered unrelated to study medication, and, in general, the nature of these serious events was related to the underlying diagnosis; 63% (5/8) led to discontinuation from the study.

©2003 American Medical Association. All rights reserved.
Downloaded From: http://archpsyc.jamanetwork.com/ by a University of St. Andrews Library User on 05/24/2015

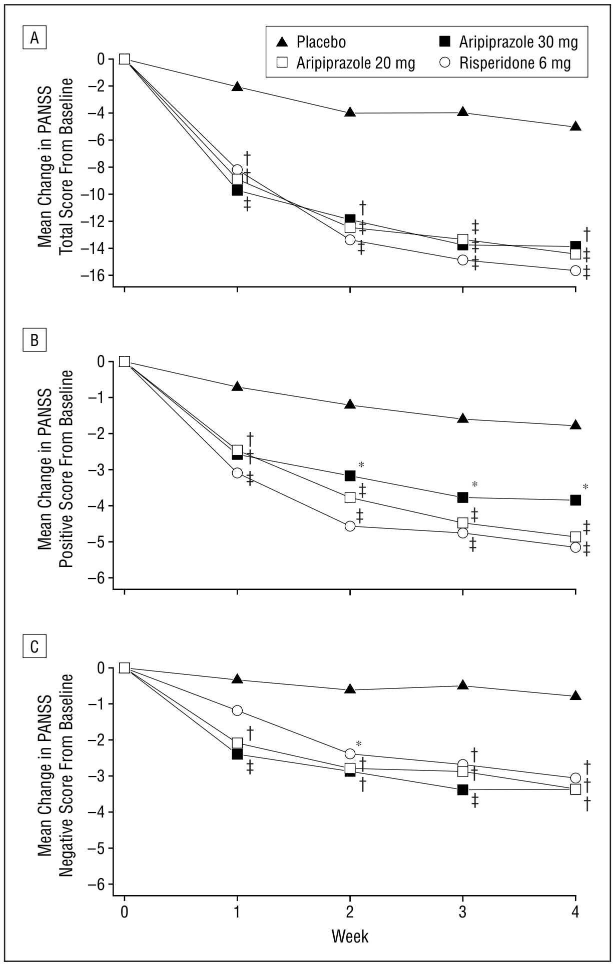

Figure 2. Mean change in Positive and Negative Syndrome Scale (PANSS) total (A), positive (B), and negative (C) scores from baseline over 4 weeks of treatment with aripiprazole (20 mg/d or 30 mg/d), risperidone (6 mg/d), or placebo (last observation carried forward). Asterisk indicates $P<.05$ vs placebo; dagger, $P<.01$ vs placebo; double dagger, $P\leq.001$ vs placebo.

Extrapyramidal Symptoms

EPS-Related Adverse Events. The overall incidence of EPS-related adverse events was comparable in the aripiprazole and risperidone groups (aripiprazole 20 mg, n=32 [32%]; aripiprazole 30 mg, n=31 [31%]; risperidone, n=31 [31%]). In the placebo group, 21 patients (20%) reported an EPS-related adverse event. Dystonia/hypertonia was reported by 14 patients (14%) in the risperidone group, compared with 3 (3%) in the aripiprazole 20-mg group, 1 (1%) in the aripiprazole 30-mg group, and 6 (6%) in the placebo group.

Simpson-Angus Scale Score. The mean change in score from baseline to last visit was –0.16 in the aripiprazole 20-mg treatment group, –0.09 in the aripiprazole 30-mg group, –0.18 in the risperidone group, and –0.29 in the placebo group. Pairwise comparisons revealed no statistically significant differences between active treatments and placebo.

Barnes Akathisia Rating Scale Global Score. The mean change in score from baseline to last visit was 0.15 in the aripiprazole 20-mg treatment group, 0.18 in the aripiprazole 30-mg group, 0.14 in the risperidone group, and 0.11 in the placebo group. Pairwise comparisons revealed no statistically significant differences between active treatments and placebo.

Abnormal Involuntary Movement Scale Score. The mean change in score from baseline to last visit was –0.27 in the aripiprazole 20-mg treatment group, –0.5 in the aripiprazole 30-mg group, –0.6 in the risperidone group, and 0.1 in the placebo group. Risperidone produced a statistically significant change compared with placebo ($P=.03$).

The use of benztropine was comparable across the 3 active treatment groups.

Body Weight

Measurements of body weight during the study showed a minimal mean increase from baseline to last study visit in all 3 active treatment groups: aripiprazole 20 mg, 1.2 kg; aripiprazole 30 mg, 0.8 kg; risperidone, 1.5 kg (**Figure 3**). These differences were statistically significant compared with the placebo group, which showed a mean decrease in body weight of –0.3 kg. The incidence of clinically significant weight gain (≥7% increase from baseline) was statistically significant compared with placebo for all active treatments: placebo, 2%; aripiprazole 20 mg, 13% ($P=.004$); aripiprazole 30 mg, 9% ($P=.04$); risperidone 6 mg, 11% ($P=.03$).

Serum Prolactin Levels

Serum prolactin levels decreased from baseline levels in both aripiprazole treatment groups (aripiprazole 20 mg, –6.6 ng/mL; aripiprazole 30 mg, –6.4 ng/mL) and increased in the placebo group (0.1 ng/mL); the changes in the aripiprazole groups were not statistically significant compared with placebo. No patients receiving aripiprazole had a serum prolactin level below the lower limit of the reference range (2 ng/mL). Risperidone produced a 47.9 ng/mL increase in mean prolactin levels, which was significantly greater than the change observed in the placebo group ($P<.001$) (**Figure 4**). The percentage of patients with an increase in serum prolactin level above 23 ng/mL (the upper limit of the reference range defined by the central clinical laboratory) in each group was placebo, 10.3%; aripiprazole 20 mg, 4.1% ($P=.16$ vs placebo); aripiprazole 30 mg, 3.3% ($P=.08$ vs placebo); and risperidone, 90.5% ($P<.001$ vs placebo).

Electrocardiograms

The QT_c interval was calculated using Bazett's formula ($QT_cB = QT/RR^{0.5}$). Mean changes in QT_c interval for each treatment group were aripiprazole 20 mg, 0.97 milliseconds; aripiprazole 30 mg, –2.35 milliseconds; placebo, –2.18 milliseconds; and risperidone, 6.31 milliseconds (**Figure 5**). No patients receiving aripiprazole or placebo experienced a potentially clinically significant increase in QT_c interval. Clinical significance was operationally defined as a QT_c of 450 milliseconds or more and a 10% or greater increase from baseline. In the risperi-

©2003 American Medical Association. All rights reserved.
Downloaded From: http://archpsyc.jamanetwork.com/ by a University of St. Andrews Library User on 05/24/2015

Table 4. Incidence of Treatment-Emergent Adverse Events (≥5% Incidence in Any Treatment Group)*

Body System/Primary Term	Placebo (n = 103)	Aripiprazole 20 mg (n = 101)	Aripiprazole 30 mg (n = 100)	Risperidone 6 mg (n = 99)
Whole body				
Headache	28 (27)	28 (28)	35 (35)	31 (31)
Asthenia	5 (5)	8 (8)	8 (8)	6 (6)
Pain	4 (4)	6 (6)	6 (6)	2 (2)
Pain, extremity	6 (6)	5 (5)	6 (6)	5 (5)
Infection	4 (4)	3 (3)	5 (5)	2 (2)
Pain, back	7 (7)	4 (4)	1 (1)	7 (7)
Accidental injury	7 (7)	5 (5)	0	0
Cardiovascular system				
Tachycardia	1 (1)	4 (4)	3 (3)	15 (15)
Digestive system				
Dyspepsia	22 (21)	16 (16)	16 (16)	12 (12)
Constipation	3 (3)	7 (7)	11 (11)	11 (11)
Vomiting	6 (6)	15 (15)	8 (8)	8 (8)
Dry mouth	3 (3)	6 (6)	6 (6)	7 (7)
Diarrhea	9 (9)	5 (5)	4 (4)	8 (8)
Nausea	10 (10)	13 (13)	4 (4)	12 (12)
Dental disorder	2 (2)	4 (4)	3 (3)	7 (7)
Musculoskeletal system				
Myalgia	3 (3)	6 (6)	6 (6)	2 (2)
Disorder, joint	2 (2)	0	2 (2)	5 (5)
Nervous system				
Agitation	24 (23)	27 (27)	29 (29)	22 (22)
Insomnia	23 (22)	31 (31)	22 (22)	20 (20)
Anxiety	19 (18)	21 (21)	20 (20)	18 (18)
Akathisia	9 (9)	20 (20)	20 (20)	14 (14)
Somnolence	11 (11)	4 (4)	19 (19)	14 (14)
Tremor	5 (5)	7 (7)	12 (12)	2 (2)
Lightheadedness	9 (9)	12 (12)	9 (9)	11 (11)
Psychosis†	10 (10)	15 (15)	7 (7)	11 (11)
Salivation increased	0	2 (2)	6 (6)	3 (3)
Extrapyramidal syndrome‡	0	6 (6)	1 (1)	0
Hypertonia	6 (6)	2 (2)	1 (1)	9 (9)
Dystonia	0	1 (1)	0	5 (5)
Respiratory system				
Upper respiratory infection	2 (2)	3 (3)	8 (8)	8 (8)
Pharyngitis	5 (5)	2 (2)	5 (5)	2 (2)
Rhinitis	1 (1)	4 (4)	4 (4)	12 (12)
Skin and appendages				
Rash	7 (7)	3 (3)	11 (11)	8 (8)
Special senses				
Blurred vision	1 (1)	3 (3)	5 (5)	4 (4)
Urogenital system				
Dysmenorrhea§	2 (7)	1 (4)	2 (6)	0
Vaginitis§	1 (3)	0	2 (6)	1 (4)

*Values are given as the number (percentage) of patients.
†Most psychosis reported was due to exacerbation of the underlying disease and was not related to study medication.
‡Included the following: extrapyramidal side effects (EPS); increased EPS = gross stiffness, drug-induced parkinsonism, and pseudoparkinsonism.
§Women: placebo, n = 30; aripiprazole 20 mg, n = 28; aripiprazole 30 mg, n = 34; and risperidone 6 mg, n = 28.

done group, 3 (3%) of 95 patients had a increase in QT_c interval during the study that met this definition.

Vital Signs and Laboratory Analyses

There were no obvious clinical differences in the vital signs detected in any of the treatment groups, and no patients discontinued from the study due to vital sign abnormalities. Other than serum prolactin levels, there were no clinically meaningful differences between groups in terms of laboratory abnormalities. One patient in the risperidone group discontinued the study due to a mild abnormality in results of liver function tests.

COMMENT

The results of the study indicated that aripiprazole 20 mg/d and 30 mg/d were effective, safe, and well tolerated for the treatment of patients with acute relapse of schizophrenia or schizoaffective disorder. Both dosages of aripiprazole were superior to placebo for treatment of both the positive and negative symptoms of schizophrenia. Statistically significant improvements compared with placebo were demonstrated in both aripiprazole treatment groups across all efficacy measures at the end point (PANSS total score, PANSS positive and negative scores,

©2003 American Medical Association. All rights reserved.
Downloaded From: http://archpsyc.jamanetwork.com/ by a University of St. Andrews Library User on 05/24/2015

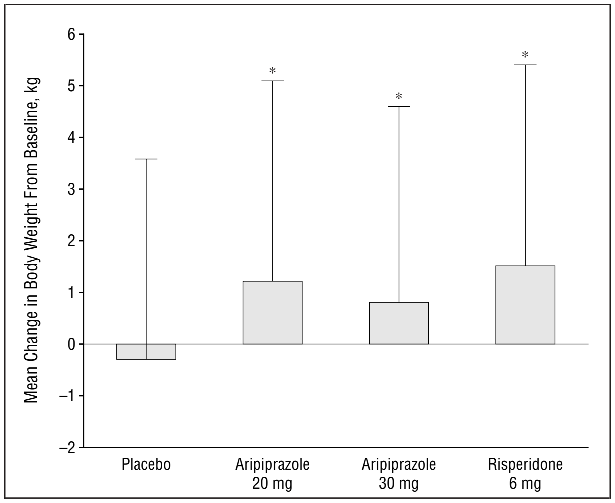

Figure 3. Mean change in weight from baseline. Asterisk indicates $P<.05$ vs placebo; error bars, SD.

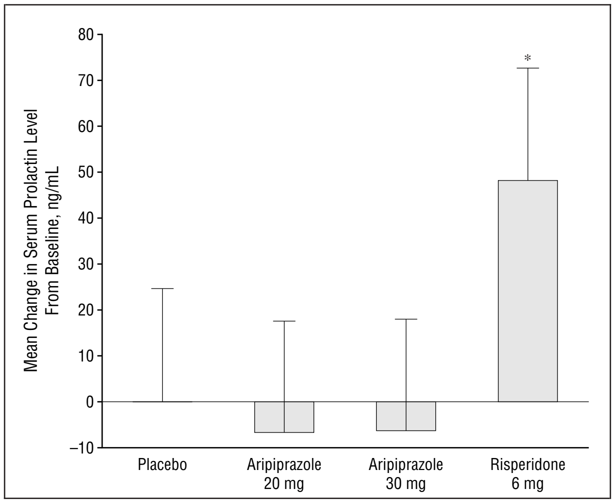

Figure 4. Mean change in prolactin levels from baseline. Asterisk indicates $P<.001$ vs placebo; error bars, SD.

CGI-S score, PANSS-derived BPRS core score, mean CGI-I score, and time to response to therapy). The improvements in symptoms seen with aripiprazole treatment were comparable with those produced by the atypical agent risperidone, which served as an active control in this study. Risperidone improved all primary and secondary efficacy variables significantly more than placebo, confirming the responsiveness of the population to active treatment and establishing the validity of the trial.

Rapid onset of efficacy was demonstrated in both aripiprazole groups. Aripiprazole doses of 20 mg and 30 mg produced statistically significant improvements compared with placebo as early as week 1 (PANSS total, PANSS positive, PANSS negative, and CGI-I scores and PANSS-derived BPRS core scores). These significant improvements were maintained through the end of the 4-week study.

These efficacy data indicate that 20-mg and 30-mg doses of aripiprazole are effective for treatment of patients with acute exacerbations of schizophrenia; a previous study demonstrated the efficacy of the 15-mg dose.[24]

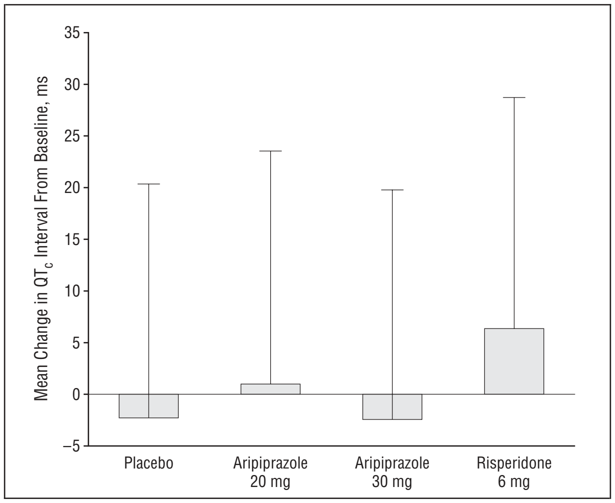

Figure 5. Mean change in corrected QT (QT_c) interval from baseline. Error bars indicate SD.

The persistence of effect throughout the 4-week duration of this study provides evidence that a dopamine-serotonin system stabilizer can produce clinically meaningful and sustained improvements in the symptoms associated with schizophrenia. These results differentiate aripiprazole from previous agents with dopamine partial agonist activity such as preclamol, (−)3-PPP, which had limited clinical utility due to lack of sustained activity.[15] The difference in the duration of effect between these agents may in part be due to differences in the intrinsic activity of each agent at D_2 receptors.[17]

The rates of discontinuation in this study were consistent with other short-term studies of atypical agents which also report higher discontinuation rates with placebo (typically ranging from 49%-80%) than with active treatment (ranging from 39%-68%).[25-30] Treatment with both dosages of aripiprazole was well tolerated. The rate of discontinuations due to adverse events was similar for all 3 active treatment groups but was higher in the placebo group. The adverse event most frequently cited as a reason for discontinuation was worsening of psychotic symptoms, which was similar in all treatment groups and was deemed to be unrelated to study medication. In this study and previous aripiprazole studies,[24,31] there does not appear to be a dose-response relationship in terms of adverse events, with the possible exception of somnolence.

Extrapyramidal symptoms have the potential to limit antipsychotic effectiveness. Neither of the aripiprazole groups showed statistically significant worsening of EPS relative to placebo at the end point on any of the EPS measures: parkinsonism (measured by the SAS), akathisia (measured by the BAS), or dyskinesia (measured by the AIMS). These results are consistent with the pooled data from the overall clinical program with aripiprazole, which indicate that the incidence of EPS with this agent is similar to that observed with placebo.[32] There was a greater incidence of reports of dystonia/hypertonia in the risperidone group. This may reflect risperidone's tendency to produce mild EPS at a 6-mg/d dosage.

One of the major differences observed between study arms in this trial was the direction of change in prolactin

©2003 American Medical Association. All rights reserved.
Downloaded From: http://archpsyc.jamanetwork.com/ by a University of St. Andrews Library User on 05/24/2015

concentrations. Significant increases in prolactin level and increased incidence of hyperprolactinemia were observed in the risperidone group throughout the study. The clinical significance of this finding could not be determined in this short-term trial. In some instances long-term hyperprolactinemia can lead to galactorrhea, amenorrhea, gynecomastia, impotence, and sexual dysfunction.[33] Neither aripiprazole group was associated with an increased rate of clinically significant elevations in serum prolactin levels. Both aripiprazole groups showed a numerical decrease in prolactin levels from baseline. Prolactin levels greater than the upper level of the reference range occurred in 10.3% of patients in the placebo group, 4.1% and 3.3% of those in the aripiprazole 20-mg and 30-mg groups, respectively, and in 90.5% of those treated with risperidone. As dopamine inhibits prolactin release, the numerical decrease in serum prolactin level with aripiprazole administration is consistent with partial agonism at the D_2 receptor and mirrors results from preclinical studies.[18]

Prolongation of the QT_c interval on ECGs has been observed in patients taking certain antipsychotic medications.[34] A prolonged QT_c interval can lead to torsade de pointes, a potentially fatal cardiac arrhythmia. Three patients in the risperidone group had what can be considered to be a clinically significant increase in QT_c interval (QT_c of $\geq$450 milliseconds and a $\geq$10% increase from baseline); the absence of any cases of clinically significant QT_c prolongation in either aripiprazole group supports a low risk for arrhythmic potential.

The issue of weight gain associated with the use of certain antipsychotics has been the focus of recent attention.[35,36] The long-term use of antipsychotics has important health implications. Increases in body weight can increase the risk of conditions such as cardiovascular disease and diabetes mellitus and can lead to decreased compliance. In the current study, the percentage of patients with a clinically significant increase in body weight was approximately the same for the 3 active treatments (9%-13%). The mean weight change for each active treatment group (0.8-1.5 kg) did not appear to be clinically meaningful, although the study was only 4 weeks in duration. Longer follow-up will be necessary to assess possible impacts on body weight more accurately.

In summary, the results of the present study indicate that aripiprazole has considerable potential for the treatment of psychotic disorders such as schizophrenia. Some of the unique clinical findings of this study may be linked to aripiprazole's unique mechanism of action, which includes potent partial agonist activity at D_2 receptors as well as partial agonist activity at $5HT_{1A}$ receptors and antagonist activity at $5HT_{2A}$ receptors. The current study demonstrates that 20-mg and 30-mg doses of aripiprazole are effective for treatment of both positive and negative symptoms. In addition, this study demonstrates that aripiprazole has a rapid onset of action with sustained efficacy during the 4 weeks of treatment. These results suggest that aripiprazole is the first molecule that is not a pure D_2 antagonist to demonstrate substantial and sustained antipsychotic efficacy. The antipsychotic effects of 20-mg and 30-mg doses of aripiprazole were achieved in the absence of adverse effects such as QT_c interval prolongation or a marked potential for EPS or weight gain. The low risk for hyperprolactinemia and EPS with aripiprazole use observed in this and other aripiprazole studies may be explained by its partial agonist profile at D_2 receptors, in contrast to the D_2 receptor blockade in the nigrostriatal and tuberoinfundibular systems produced by currently available antipsychotics. This combination of sustained efficacy with a favorable safety and tolerability profile may lead to increased treatment adherence and decreased relapse rates over the long term. The results of the current study suggest that aripiprazole may represent an important new option for the treatment of schizophrenia and schizoaffective disorder.

Submitted for publication April 4, 2002; final revision received January 15, 2003; accepted January 22, 2003.

From the Brain Imaging Center, University of California, Irvine (Dr Potkin); Otsuka Maryland Research Institute, Rockville, Md (Drs Saha, Ali, and Ingenito); Bristol-Myers Squibb, Princeton, NJ (Dr Kujawa); Otsuka America Pharmaceuticals Inc, Princeton (Dr Carson); Bristol-Myers Squibb, Wallingford, Conn (Dr Stock and Mr Stringfellow); and the Department of Psychiatry and Biobehavioral Sciences, David Geffen School of Medicine, University of California, Los Angeles (Dr Marder). *Dr Potkin has received funding from Arena Pharmaceuticals, Inc (San Diego, Calif), AstraZeneca Pharmaceuticals LP (Wilmington, Del), Bristol-Myers Squibb (New York, NY), Eli Lilly and Company (Indianapolis, Ind), Forest Laboratories, Inc (New York, NY), Janssen Pharmaceutica (Titusville, NJ), Novartis AG (Basel, Switzerland), Organon (Roseland, NJ), Otsuka Pharmaceuticals (Rockville, Md), Pfizer Inc (New York, NY), and Sanofi-Synthélabo (Paris, France); he has served as a consultant for and/or on the advisory board of Acadia Pharmaceuticals Inc (San Diego, Calif), Arena Pharmaceuticals, Inc, AstraZeneca Pharmaceuticals LP, Bristol-Myers Squibb, Janssen Pharmaceutica, Novartis AG, Organon, Otsuka Pharmaceuticals, Pfizer Inc, and Praecis Pharmaceuticals (Waltham, Mass); and he has served on the speakers bureau of AstraZeneca Pharmaceuticals LP, Bristol-Myers Squibb, Novartis AG, and Pfizer Inc.*

This study was supported by Bristol-Myers Squibb and Otsuka Pharmaceuticals.

This study was presented at the Biennial Winter Workshop for Schizophrenia Research; February 26, 2002; Davos, Switzerland.

Corresponding author and reprints: Steven G. Potkin, MD, Brain Imaging Center, University of California, Irvine, Irvine Hall, Room 166, Irvine, CA 92697 (e-mail: sgpotkin@uci.edu).

REFERENCES

1. Meltzer HY. The role of serotonin in antipsychotic drug action. *Neuropsychopharmacology.* 1999;21(2 suppl):106S-115S.
2. Millan MJ. Improving the treatment of schizophrenia: focus on serotonin (5-HT1A) receptors. *J Pharmacol Exp Ther.* 2000;295:853-861.
3. Roth BL, Sheffler D, Potkin SG. Atypical antipsychotic drug action: unitary or multiple mechanisms for "atypicality." *Clin Neurosci.* In press.
4. Kapur S, Seeman P. Antipsychotic agents differ in how fast they come off the dopamine D2 receptors: implications for atypical antipsychotic action. *J Psychiatry Neurosci.* 2000;25:161-166.
5. Kapur S, Seeman P. Does fast dissociation from the dopamine D2 receptor explain the action of atypical antipsychotics? a new hypothesis. *Am J Psychiatry.* 2001;158:360-369.

©2003 American Medical Association. All rights reserved.
Downloaded From: http://archpsyc.jamanetwork.com/ by a University of St. Andrews Library User on 05/24/2015

6. Seeman P, Tallerico T. Antipsychotic drugs which elicit little or no parkinsonism bind more loosely than dopamine to brain D2 receptors, yet occupy high levels of these receptors. *Mol Psychiatry*. 1998;3:123-134.
7. Fleischhacker WW, Meise U, Gunther V, Kurz M. Compliance with antipsychotic drug treatment: influence of side effects. *Acta Psychiatr Scand Suppl*. 1994;382: 11-15.
8. Kurzthaler I, Fleishhacker WW. The clinical implications of weight gain in schizophrenia. *J Clin Psychiatry*. 2001;62(suppl 7):32-37.
9. Meltzer HY, Stahl SM. The dopamine hypothesis of schizophrenia: a review. *Schizophr Bull*. 1976;2:19-76.
10. Stahl SM. *Essential Psychopharmacology*. New York, NY: Cambridge University Press; 1996.
11. Richelson E. Receptor pharmacology of neuroleptics: relation to clinical effects. *J Clin Psychiatry*. 1999;60(suppl 10):5-14.
12. Naber D, Gaussares C, Moeglen JM, Tremmel L, Bailey PE, and the SDZ HDC 912 Collaborative Study Group. Efficacy and tolerability of SDZ HDC 912, a partial dopamine D-2 agonist, in the treatment of schizophrenia. In: Meltzer HY, ed. *New Research Directions in the Development of Atypical and Other Novel Antipsychotic Medications*. New York, NY: Raven Press; 1991.
13. Olbrich R, Schanz H. The effect of the partial dopamine agonist terguride on negative symptoms in schizophrenics. *Pharmacopsychiatry*. 1988;21:389-390.
14. Olbrich R, Schanz H. An evaluation of the partial dopamine agonist terguride regarding positive symptoms reduction in schizophrenics. *J Neural Transm Gen Sect*. 1991;84:233-236.
15. Lahti AC, Weiler MA, Corey PK, Lahti RA, Carlsson A, Tamminga CA. Antipsychotic properties of the partial dopamine agonist (–)-3-(3-hydroxyphenyl)-N-n-propylpiperidine (preclamol) in schizophrenia. *Biol Psychiatry*. 1998;43:2-11.
16. Tamminga CA. Partial dopamine agonists in the treatment of psychosis. *J Neural Transm*. 2002;109:411-420.
17. Burris KD, Molski TF, Xu C, Ryan E, Tottori K, Kikuchi T, Yocca FD, Molinoff PB. Aripiprazole, a novel antipsychotic, is a high affinity partial agonist at human dopamine D2 receptors. *J Pharmacol Exp Ther*. 2002;302:381-389.
18. Inoue T, Domae M, Yamada K, Furukawa T. Effects of the novel antipsychotic agent 7-[4-[(2,3-dichlorophenyl)-1-piperazinyl]butyoxy]-3,4-dihydro-2 (1H)-quinolinone (OPC-14597) on prolactin release from the rat anterior pituitary gland. *J Pharmacol Exp Ther*. 1996;277:137-143.
19. Kikuchi T, Tottori K, Uwahodo Y, Hirose T, Miwa T, Oshiro Y, Morita S. 7-{4-[(2,3-Dichlorophenyl)-1-piperazinyl]butyoxy}-3,4-dihydro-2 (1H)-quinolinone (OPC-14597), a new putative antipsychotic drug with both presynaptic and postsynaptic D2 receptor antagonist activity. *J Pharmacol Exp Ther*. 1995;274:329-336.
20. Jordan S, Koprivica V, Chen R, Tottori K, Kikuchi T, Altar CA. The antipsychotic aripiprazole is a potent, partial agonist at the human 5-HT1A receptor. *Eur J Pharmacol*. 2002;441:137-140.
21. McQuade RD, Burris KD, Jordan S, Tottori K, Kurahashi N, Kikuchi T. Aripiprazole: a dopamine-serotonin system stabilizer [abstract]. *Int J Neuropsychopharmacol*. 2002;5(suppl 1):S176.
22. Leysen JE, Janssen PM, Schotte A, Luyten WH, Megens AA. Interaction of antipsychotic drugs with neurotransmitter receptor sites in vitro and in vivo in relation to pharmacological and clinical effects: role of 5HT2 receptors. *Psychopharmacology (Berl)*. 1993;112(1 suppl):S40-S54.
23. Rao ML, Möller HJ. Biochemical findings of negative symptoms in schizophrenia and their putative relevance to pharmacologic treatment: a review. *Neuropsychobiology*. 1994;30:160-172.
24. Kane JM, Carson WH, Saha AR, McQuade RD, Ingenito GG, Zimbroff DL, Ali MW. Efficacy and safety of aripiprazole and haloperidol versus placebo in patients with schizophrenia and schizoaffective disorder. *J Clin Psychiatry*. 2002;63:763-771.
25. Beasley CM Jr, Sanger T, Satterlee W, Tollefson G, Tran P, Hamilton S. Olanzapine versus placebo: results of a double-blind, fixed-dose olanzapine trial. *Psychopharmacology (Berl)*. 1996;124:159-167.
26. Beasley CM Jr, Tollefson G, Tran P, Satterlee W, Sanger T, Hamilton S. Olanzapine versus placebo and haloperidol: acute phase results of the North American double-blind olanzapine trial. *Neuropsychopharmacology*. 1996;14:111-123.
27. Beasley CM Jr, Hamilton SH, Crawford AM, Deliva MA, Tollefson GD, Tran PV, Beuzen JN. Olanzapine versus haloperidol: acute phase results of the international double-blind trial. *Eur Neuropsychopharmacol*. 1997;7:125-137.
28. Arvanitis LA, Miller BG. Multiple fixed doses of "Seroquel" (quetiapine) in patients with acute exacerbation of schizophrenia: a comparison with haloperidol and placebo: the Seroquel Trial 13 Study Group. *Biol Psychiatry*. 1997;42:233-246.
29. Small JG, Hirsch SR, Arvanitis LA, Miller BG, Link CG. Quetiapine in patients with schizophrenia: a high- and low-dose double-blind comparison with placebo: Seroquel Study Group. *Arch Gen Psychiatry*. 1997;54:549-557.
30. Borison RL, Arvanitis LA, Miller BG. ICI 204,636, an atypical antipsychotic: efficacy and safety in a multicenter, placebo-controlled trial in patients with schizophrenia: US Seroquel Study Group. *J Clin Psychopharmacol*. 1996;16:158-169.
31. Daniel DG, Saha AR, Ingenito G, Carson WH, Dunbar GC. Aripiprazole, a novel antipsychotic: overview of a phase II study result [abstract]. *Int J Neuropsychopharmacol*. 2000;3(suppl 1):S157.
32. Marder SR, McQuade RD, Stock E, Kaplita S, Marcus R, Safferman AZ, Saha A, Ali M, Iwamoto T. Aripripazole in the treatment of schizophrenia: safety and tolerability in short-term, placebo-controlled trials. *Schizophr Res*. 2003;61:123-126.
33. Dickson RA, Glazer WM. Neuroleptic-induced hyperprolactinemia. *Schizophr Res*. 1999;35(suppl):S75-S86.
34. Gury C, Canceil O, Iaria P. Antipsychotic drugs and cardiovascular safety: current studies of prolonged QT interval and risk of ventricular arrhythmia. *Encephale*. 2000;26(6):62-72.
35. Allison DB, Casey DE. Antipsychotic-induced weight gain: a review of the literature. *J Clin Psychiatry*. 2001;62:22-31.
36. Allison DB, Mentore JL, Heo M, Chandler LP, Cappelleri JC, Infante MC, Weiden PJ. Antipsychotic induced weight gain: a comprehensive research synthesis. *Am J Psychiatry*. 1999;156:1686-1696.

©2003 American Medical Association. All rights reserved.
Downloaded From: http://archpsyc.jamanetwork.com/ by a University of St. Andrews Library User on 05/24/2015
