# Supplementary material for: Dose-Dependent Efficacy of Aripiprazole in Treating Patients With Schizophrenia or Schizoaffective Disorder: A Systematic Review and Meta-Analysis of Randomized Controlled Trials
Source: Front Psychiatry. 2021 Aug 11;12:717715. doi: 10.3389/fpsyt.2021.717715 (PMC8385236; doi:10.3389/fpsyt.2021.717715)
Supplement: Supplementary file 1 [file Data_Sheet_1.ZIP › supplementray material-9 RCTs/5 Wen 2014.pdf]

利培酮添加不同剂量的阿立哌唑对稳定期精神分裂症患者认知功能的影响

闻荣海[1] 刘 史[2]

1.河南省南阳市精神病医院药学部,河南南阳 473000；2.河南省南阳市精神病医院六病区,河南南阳 473000

[摘要] 目的 探求阿立哌唑的剂量对服用利培酮的稳定期门诊精神分裂症患者认知功能的影响。方法 将 43 例服用利培酮的稳定期门诊精神分裂症患者随机分为两组,对照组(n=22)添加(20~30) mg/d 阿立哌唑,研究组(n=21)添加(5~15) mg/d 阿立哌唑,治疗时间 12 周。分别用精神分裂症认知功能简明成套测评量表(BACS)中文版和阳性和阴性症状量表(PANSS)评估疗效,用副反应量表(TESS)评价治疗期间发生的不良反应。结果 两组不良反应发生率比较,差异无统计学意义(P>0.05),利培酮添加(5~15) mg/d 阿立哌唑对患者的部分认知功能和阴性症状有明显改善,而添加(20~30) mg/d 阿立哌唑,尽管和前者一样能改善运动速度(MS)和阴性症状,但对工作记忆(WM)和执行功能(EF)有显著损害(P<0.01)。结论 利培酮添加低剂量而非高剂量的阿立哌唑对认知功能改善最佳。这一结果仍需大量和长期的研究加以确定。

[关键词] 阿立哌唑；剂量；认知功能；D2 受体占有率;5HT1A/D2 受体部分激动剂的平衡状态

[中图分类号] R749.3 [文献标识码] B [文章编号] 1673-9701(2014)26-0061-03

Effects of different dosages of aripiprazole on cognitive function in stabilized schizophrenic patients treated with risperidone

WEN Ronghai[1] *LIU Shi*[2]

1.Department of Pharmacy, Psychiartric Hospital of Nanyang City in He'nan Province, Nanyang 473000,China; 2.NO. 6 Ward,Psychiartric Hospital of Nanyang City in He'nan Province, Nanyang 473000,China

[Abstract] Objective To probe the effects of dosages of aripiprazole on cognitive function of stabilized schizophrenic outpatients treated with risperidone. **Methods** A totol of 43 stabilized schizophrenic outpatients received risperidone were randomly divided into two groups. Aripiprazole added to control group (n=22)was taken by (20~30) mg/d and the study group(n=21)did by (5~15) mg/d,respectively, for 12 weeks. Brief Assessment of Cognition in Schizophrenia (BACS, Chinese edition)and Positive and Negative Syndrome Scale (PANSS)were used to evaluate efficacy. Treatment Emergent Symptom Scale (TESS) was used to evaluate adverse effects during this trial. **Results** The occurring rates of adverse effects for the two groups were not significantly different (P>0.05). Risperidone augmentation with aripiprazole by (5~15) mg/d significantly improved partial cognitive function and negative symptoms for these patients. Add-on treatment with aripiprazole by (20~30) mg/d significantly impair working memory(WM) and executive function(EF) (P<0.01), although that significantly improved motor speed (MS) and negative symptoms as the former did. **Conclusion** Risperidone augmentation with a lower rather than higher dosage of aripiprazole would improve cognitive function most favorably. These findings need further substantial and long-term trials to be established .

[Key words] Aripiprazole; Dosage; Cognitive function; D2 receptor occupancy; Balancing status between 5HT1A/D2 partial agonists

认知缺陷是精神分裂症的基本症状之一，适当剂量的经典和非经典抗精神病药在治疗精神症状的同时,对认知缺陷均具有中度改善作用[1],但剂量加大,即多巴胺 D2 受体占有率超过 77%,会对认知功能造成损害[2],阿立哌唑与其他抗精神病药相比具有独特的药理学特性，既是多巴胺 D2 受体部分激动剂,也是 5-羟色胺 5HT1A 受体部分激动剂,同时对 5HT2A 受体也有拮抗作用。这些优点对改善认知和阴性症状均有利,但阿立哌唑的剂量对认知缺陷的影响研究极少,甚至出现不一致[3,4],本研究通过应用不同剂量的阿立哌唑联合利培酮的观察对比,对此问题作进一步探讨。

1 对象与方法

1.1 研究对象

选择 2013 年 3~7 月在我院就诊的稳定期门诊精

(C)1994-2019 China Academic Journal Electronic Publishing House. All rights reserved. http://www.cnki.net

神分裂症患者为研究对象，入组标准：①符合 DSM-IV 精神分裂症诊断标准；②服用利培酮剂量稳定，至少 3 个月；③年龄 19~55 岁；④未联用其他抗精神病药；⑤获患者或家属知情同意。排除标准：①妊娠和哺乳；②酒和药物依赖；③病情不稳或有严重神经系统疾病；④服药期间吸烟；⑤联用抗抑郁药或心境稳定剂。

1.2 方法

收集符合上述条件的稳定期门诊精神分裂症患者 43 例，随机分为两组，对照组(n=22)添加阿立哌唑(阿立哌唑口腔崩解片，规格：5 mg，成都康弘药业集团股份有限公司生产，国药准字：H20060521)(20~30) mg/d，研究组(n=21)添加阿立哌唑(5~15) mg/d，两组利培酮(利培酮片，规格：1mg，江苏恩华药业有限公司生产，国药准字：H20050160)的剂量分别为(5.22±1.02)mg、(5.19±1.12)mg，差异无显著性(P>0.05)，原利培酮剂量不改变，2 周后阿立哌唑添加至稳定剂量[(24.1±4.0)、(8.6±3.2) mg](P<0.01)，去除抗胆碱药，若仍有锥体外系反应用普萘洛尔替换，如有激越、失眠可用阿普唑仑处理。两组人口学基本资料均衡，即在年龄[(34.7±10.0)岁、(35.6±10.2)岁]、病程[(7.5±3.1)年、(7.8±3.5)年]、受教育年限[(10.4±5.0)年、(9.8±5.2)年]和性别[(男 14 例，女 8 例)、(男 11 例，女 10 例)]等方面均无显著差异(P>0.05)。

1.3 疗效及安全性评价

主要效果用精神分裂症认知功能简明成套测评量表中文版[5](Brief Assessment of Cognition in Schizophrenia，BACS)分别于治疗前，治疗后第 12 周测评，由 3 名受过 BACS 严格培训的心理测评师完成(kappa>0.8)。次要效果用阳性和阴性症状量表(PANSS)分别于治疗前，治疗后第 12 周测评由 3 名受过 PANSS 培训并合格的精神科主治医师完成(kappa>0.8)。不良反应用副反应量表(TESS)评定。一次 BACS 测评约需 30 min 完成，它由语言记忆、数字序列、代币运动任务、语言流畅性、字母流畅性、符号编码、伦敦塔 7 个亚表组成，分别对语言记忆(VM)、工作记忆(WM)、运动速度(MS)、语言流畅性(VF)、注意力和加工速度(A/PS)及执行功能(EF)进行测评。PANSS 量表由阳性症状分量表(PANSS-P)，阴性症状量表(PANSS-N)和一般精神病理量表(PANSS-G)，各分量表得分合计为总量表(PANSS-T)得分，共 30 项。

1.4 统计学方法

采用 SPSS 17.0 统计学软件进行数据处理分析，计量资料以均数±标准差($\bar{x}\pm s$)表示，组内比较用配对 t 检验，组间比较用 t 检验，两组人口学基本资料用 t 检验或 χ^2 检验。P<0.05 为差异有统计学意义。

2 结果

研究组有 1 例因不良反应退出本试验，对照组 1 例因不良反应、1 例因病情加剧而退出本试验。

2.1 主要效果和次要效果

治疗前两组 BACS 和 PANSS 各项评分比较均无统计学意义(P>0.05)。研究组添加(5~15) mg/d 的阿立哌唑后第 12 周，在语言记忆、运动速度、警觉和加工速度和 PANSS-N 分量表评分方面有显著改善，而对照组添加(20~30) mg/d 的阿立哌唑后第 12 周，仅在运动速度方面和 PANSS-N 分量表评分方向有明显改善，对工作记忆和执行功能却有显著损害。治疗后两组在语言记忆(t=2.40，P<0.05)、警觉和加工速度(t=2.20，P<0.05)、工作记忆(t=3.84，P<0.01)和执行功能(t=4.08，P< 0.01)等方面评分比较，差异有统计学意义。但运动速度(t=0.19，P>0.05)和 PANSS-N 分量表评分(t=-0.81，P>0.05)方面比较差异无统计学意义。其他如语言流畅性(t=1.47，P>0.05)、PANSS-P(t=-0.08，P>0.05)，PANSS-G(t=0.36，P>0.05)和 PANSS-T(t=-0.30，P>0.05)评分方面比较差异无统计学意义。见表 1。总之，利培酮添加阿立哌唑(低剂量或高剂量)后，对运动速度和阴性症状有明显改善，另外，添加低剂量阿立哌唑还可改善语言记忆、警觉和加工速度，但添加高剂量则对工作记忆和执行功能造成损害。这一结果与某些研究大体上一致[3,6,7]。

表 1 两组 12 周BACS 各区域评分和 PANSS 评分的变化

项目	研究组(n=20)		t 值	对照组(n=20)		t 值
	治疗前	治疗后		治疗前	治疗后	
BACS						
VM	30.6±8.2	37.3±8.5	-3.83**	31.4±8.5	30.2±10.2	0.52
WM	15.0±3.5	15.7±3.6	-0.74	15.3±3.9	11.8±2.7	3.17**
MS	64.0±14.8	76.8±11.0	-3.23**	66.4±15.5	76.2±10.7	-2.30*
VF	31.3±5.7	32.1±5.3	-0.51	31.7±5.9	29.7±5.2	1.49
A/PS	47.3±12.1	53.2±10.7	-2.51*	48.9±12.2	45.1±12.4	1.73
EF	15.6±2.7	16.1±3.0	-0.67	16.2±3.3	12.4±2.7	4.34**
PANSS						
PANSS-P	11.9±3.2	12.4±4.3	-0.61	11.6±3.8	12.5±3.7	-0.83
PANSS-N	16.4±5.7	12.2±4.7	2.62*	16.6±6.4	13.4±5.1	2.63*
PANSS-G	27.4±4.4	28.6±5.1	-0.75	27.1±5.2	28.0±5.3	-0.37
PANSS-T	55.6±10.1	53.1±8.9	0.88	55.6±10.6	53.9±7.1	0.70

注：组内比较，*P<0.05，**P<0.01

2.2 不良反应

对照组主要不良反应有恶心呕吐(14.3%)、失眠(9.5%)、焦虑(9.5%)、静坐不能(19.0%)、激越(4.8%)等，共有 8 例发生 12 例次不良反应，研究组主要不良反应有恶心(13.6%)、失眠(9.0%)、焦虑(9.0%)、静坐不能(22.7%)、激越(9.0%)等，共有 10 例发生 15 例次不良反应。两组不良反应发生率比较，差异无统计学意义(χ^2=0.56，P>0.05)，不良反应多可以处理，有的随治疗时间延长消失或能耐受。

(C)1994-2019 China Academic Journal Electronic Publishing House. All rights reserved. http://www.cnki.net

3 讨论

5HT1A 受体部分激动剂和 D2 受体部分激动剂（或拮抗剂）之间的平衡作用是新一代抗精神病药研究的重点，因为这些药物在治疗精神症状的同时，可以缓解认知缺陷和阴性症状，且较少引起锥体外系不良反应和代谢障碍[8]。低剂量的阿立哌唑对 D2 受体占有率较低，对脑代谢和认知功能的损害较小[3]，由于利培酮对 5HT1A 受体有拮抗作用，其内在活性远比阿立哌唑低[9]，联合低剂量的 5HT1A 受体部分激动剂阿立哌唑后，可提高 5HT1A 受体的内在活性。此时，相对于 D2 受体，5HT1A 受体的激动作用占主导，有利于认知功能的改善。其次，利培酮超过 5 mg/d 对认知有损害[10]，本研究收集对象 60%以上剂量达 6 mg/d。阿立哌唑对 D2 受体的亲合力最强，与利培酮联用时可以竞争性地占有 D2 受体，因此，联合低剂量的阿立哌唑后可能改善了原 5HT1A/D2 受体部分激动剂之间的平衡，缓解了利培酮剂量高导致的锥体外系反应和由此造成对语言记忆，运动速度、注意力和加工速度的影响[4]。相反高剂量的阿立哌唑对 D2 受体占有率很高，对脑代谢和认知功能可能有损害[3]。D1 受体激动剂可改善认知功能，阿立哌唑对 D1 受体亲和力很低[7]，故联合高剂量的阿立哌唑可能扭转了 5HT1A/D2 受体部分激动剂之间的最佳平衡，对部分认知功能造成了损害，但运动速度的改善并未受剂量大小的影响。

本研究结果对优化药物治疗方案有益。已经证明，阿立哌唑有明显的量效关系，最佳剂量为 10 mg/d，超过此剂量疗效不增加，也无其他益处[11]。因此使用低剂量的阿立哌唑，既可改善精神症状和认知功能，也可避免认知功能的损伤。

本研究与一些研究的结果不一致，有研究显示阿立哌唑与认知功能的改变没有剂量依赖关系，这可能与实验设计不同有关，其研究对象有许多吸烟患者[4]，吸烟对精神分裂症患者的认知缺陷有自我治疗的影响[12]。也有研究显示阿立哌唑对慢性精神分裂症患者的认知无改善作用，这可能是因为其研究对象是长期住院患者，病程超过 20 年，病程过长，患者对认知功能的改善不明显[13]，且研究期间允许使用抗胆碱药，抗胆碱药对认知功能有损害。而本研究患者病程较短，未超过 8 年，抗胆碱药在研究初期被去除。

由于研究样本较小，没有设计安慰剂对照，研究对象的范围较窄，研究时间也较短等，因此，本研究仍需大量、多方面和更为严格的研究加以确定。

[参考文献]

[1] Davidson M, Galderisi S, Weiser M, et al. Cognitive effects of antipsychotic drugs in first-episode schizophrenia and schizophreniform disorder:a randomized, open-label clinical trial(EUFEST)[J]. Am J Psychiatry, 2009, 166(6):675-682.

[2] Sakurai H, Bies RR, Stroup ST, et al. Dopamine D2 receptor occupancy and cognition in schizophrenia: Analysis of the CATIE data[J]. Schizophr Bull, 2013, 39(3):564-574.

[3] Kim E, Howes OD, Turkheimer FE, et al. The relationship between antipsychotic D2 occupancy and change in frontal metabolism and working memory:A dual [(11)C]raclopride and [(18)F]FDG imaging study with aripiprazole[J]. Psychopharmacology(Berl), 2013, 227(2):221-229.

[4] Hori H, Yoshimura R, Katsuki A, et al. The cognitive profile of aripiprazole differs from that of other atypical antipsychotics in schizophrenia patients[J]. J Psychiatr Res, 2012, 46(6):757-761.

[5] 郝世胜，王惠玲，张静静，等. 精神分裂症认知功能简明成套测评量表（中文版，BACS）信度、效度研究[J]. 精神医学杂志，2009，22(3)：170-173.

[6] Bervoets C, Morrens M, Vansteelandt K, et al. Effect of aripiprazole on verbal memory and fluency in schizophrenic patients: results from the ESCAPE study[J]. CNS Drugs, 2012, 26(11):975-982.

[7] Yasui-Furukori N, Kaneda A, Sugawara N, et al. Effect of adjunctive treatment with aripiprazole to atypical antipsychotics on cognitive function in schizophrenia patients[J]. J Psychopharmacol, 2012, 26(6):806-812.

[8] Newman-Tancredi A. The importance of 5-HT1A receptor agonism in antipsychotic drug action: Rationale and perspectives[J]. Curr Opin Investig Drugs, 2010, 11(7):802-812.

[9] Lerond J, Lothe A, Ryvlin P, et al. Effects of aripiprazole, risperidone, and olanzapine on 5-HT1A receptors in patients with schizophrenia[J]. J Clin Psychopharmacol, 2013, 33(1):84-89.

[10] Elie D, Poirier M, Chianetta J, et al. Cognitive effects of antipsychotic dosage and polypharmacy:a study with the BACS in patients with schizophrenia and schizoaffective disorder[J]. J Psychopharmacol, 2010, 24(7):1037-1044.

[11] Sparshatt A, Taylor D, Patel MX, et al. A systematic review of aripiprazole-dose, plasma concentration, receptor occupancy, and response implications for therapeutic drug monitoring[J]. J Clin Psychiatry, 2010, 71(11):1447-1456.

[12] Winterer G. Why do patients with schizophrenia smoke? [J]. Curr Opin Psychiatry, 2010, 23(2):112-119.

[13] Lee BJ, Lee SJ, Kim MK, et al. Effect of aripiprazole on cognitive function and hyperprolactinemia in patients with schizophrenia treated with risperidone[J]. Clin Psychopharmacol Neurosci, 2013, 11(2):60-66.

（收稿日期：2014-05-23）

(C)1994-2019 China Academic Journal Electronic Publishing House. All rights reserved. http://www.cnki.net
